# Eighth Annual Conference of inVIVO Planetary Health: From Challenges to Opportunities

**DOI:** 10.3390/ijerph16214302

**Published:** 2019-11-05

**Authors:** Susan L. Prescott, Trevor Hancock, Jeffrey Bland, Matilda van den Bosch, Janet K. Jansson, Christine C. Johnson, Michelle Kondo, David Katz, Remco Kort, Anita Kozyrskyj, Alan C. Logan, Christopher A. Lowry, Ralph Nanan, Blake Poland, Jake Robinson, Nicholas Schroeck, Aki Sinkkonen, Marco Springmann, Robert O. Wright, Ganesa Wegienka

**Affiliations:** 1 The ORIGINS Project, Telethon Kids Institute, University of Western Australia, Perth Childrens Hospital, Nedlands, WA 6009, Australia; 2 inVIVO Planetary Health of the Worldwide Universities Network (WUN), West New York, NJ 10704, USA; alanxlogan@gmail.com; 3 School of Public Health and Social Policy (retired), University of Victoria, Victoria, BC V8W 2Y2, Canada; thancock@uvic.ca; 4 Personalized Lifestyle Medicine Institute, Tacoma, WA 98443, USA; jeffbland@plminstitute.org; 5 Department of Forest and Conservation Sciences, University of British Columbia, Vancouver, BC V6T 1Z4, Canada; matilda.vandenbosch@ubc.ca; 6 Pacific Northwest National Laboratory, Biological Sciences Division, Richland, WA 99352, USA; janet.jansson@pnnl.gov; 7 Henry Ford Health System and Center for Urban Responses to Environmental Stressors (CURES), Wayne State University Detroit, MI 48202, USA; CJOHNSO1@hfhs.org (C.C.J.); gwegien1@hfhs.org (G.W.); 8 USDA Forest Service, Northern Research Station, Philadelphia, PA 19103, USA; michelle.c.kondo@usda.gov; 9 Yale-Griffin Prevention Research Center, Yale University, Derby, CT 06418, USA; davkatz7@gmail.com; 10 Department of Molecular Cell Biology, VU University Amsterdam (VUA), 1081 HV Amsterdam, The Netherlands; r.kort@vu.nl; 11 Department of Pediatrics, University of Alberta, Edmonton, AB T6G 2R3, Canada; kozyrsky@ualberta.ca; 12 Department of Integrative Physiology, University of Colorado Boulder, Boulder, CO 80309, USA; christopher.lowry@colorado.edu; 13 Charles Perkins Centre Nepean, University of Sydney, Camperdown, NSW 2006, Australia; ralph.nanan@sydney.edu.au; 14 Dalla Lana School of Public Health, University of Toronto, Toronto, ON M5T, Canada; blake.poland@utoronto.ca; 15 Department of Landscape, University of Sheffield, Sheffield S10 2TN, UK; jmrobinson3@sheffield.ac.uk; 16 School of Law Detroit, University of Detroit Mercy, MI 48226, USA; schroenj@udmercy.edu; 17 Ecosystems and Environment Research Program, University of Helsinki, 15140 Lahti, Finland; aki.sinkkonen@helsinki.fi; 18 Nuffield Department of Population Health, University of Oxford, Oxford OX3 7LF, UK; marco.springmann@dph.ox.ac.uk; 19 Icahn School of Medicine at Mount Sinai, New York, NY 10029, USA; robert.wright@mssm.edu

**Keywords:** planetary health, biodiversity, microbiome, rewilding, dysbiotic drift, mental health, green space, climate change, green prescriptions, nature relatedness, solastalgia, food systems, birth cohorts, social justice, inflammation, NCDs, positive emotions, mindsets, personalized medicine, narrative medicine, stress, allergy, obesity, health equity, cultural competency, indigenous health, environmental health, ecology, extinction of experience, DOHaD, art and creativity, biophilosophy, legal perspectives, health promotion

## Abstract

inVIVO Planetary Health (inVIVO) is a progressive scientific movement providing evidence, advocacy, and inspiration to align the interests and vitality of people, place, and planet. Our goal is to transform personal and planetary health through awareness, attitudes, and actions, and a deeper understanding of how all systems are interconnected and interdependent. Here, we present the abstracts and proceedings of our 8th annual conference, held in Detroit, Michigan in May 2019, themed “From Challenges, to Opportunities”. Our far-ranging discussions addressed the complex interdependent ecological challenges of advancing global urbanization, including the biopsychosocial interactions in our living environment on physical, mental, and spiritual wellbeing, together with the wider community and societal factors that govern these. We had a strong solutions focus, with diverse strategies spanning from urban-greening and renewal, nature-relatedness, nutritional ecology, planetary diets, and microbiome rewilding, through to initiatives for promoting resilience, positive emotional assets, traditional cultural narratives, creativity, art projects for personal and community health, and exploring ways of positively shifting mindsets and value systems. Our cross-sectoral agenda underscored the importance and global impact of local initiatives everywhere by contributing to new normative values as part of a global interconnected grass-roots movement for planetary health.

## 1. Introduction

**Figure 1:**
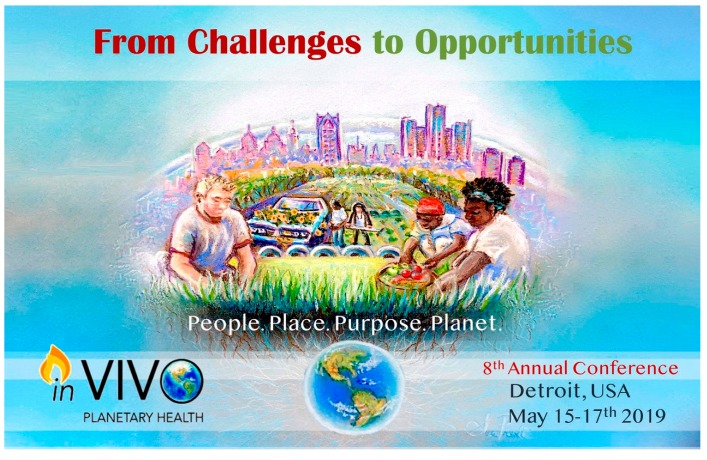
The theme of the 8th annual conference of inVIVO Planetary Health, held in Detroit, Michigan from 15–17 May, 2019 was “From Challenges, to Opportunities”.

The 8th annual conference of inVIVO Planetary Health (inVIVO), was held in Detroit, Michigan from 15 to 17 May, 2019. Our theme, “From Challenges, to Opportunities”, addressed the complex interdependent ecological challenges of advancing global urbanization and the impact on personal, environmental, economic, and societal health alike (Figure 1). Broadly, we discussed the multi-faceted dimensions of global ‘dysbiotic drift’ (life in distress) [1] on every level and explored strategies to overcome this.

The theme of the meeting reflected the ongoing inVIVO agenda of understanding and improving the complex relationships that underpin global health in all systems and on all scales (Box 1, below and also on our website at www.invivoplanet.com). The human health crisis cannot be separated from the erosion of our physical, emotional, social, economic, and political environments, underscoring the imperative for integrated multilateral solutions.

To address this, our program brought together diverse international contributors to provide perspectives from many disciplines and many geographical regions. Specific discussions included the effect of eco-biological interactions in our living environment (including urbanization, food systems, education, social inequity, climate change, biodiversity loss, and microbial ecology) on physical, mental, and spiritual wellbeing, together with the wider community and societal forces that govern these.

With a solutions-orientated focus, we explored strategies to improve the health of “people, place, purpose, and planet” across multiple domains. These domains ranged from urban-greening and renewal, nature-relatedness, nutritional ecology, planetary diets, and microbiome rewilding, through to initiatives for promoting resilience, positive emotional assets, traditional cultural narratives, creativity, art projects for personal and community health, and exploring ways of positively shifting normative value systems. In particular, we focused on exploring the interconnections between these diverse perspectives, recognizing the biology of complexity and seeking “opportunities through connectivity”.

As always, we provided a strong developmental “life-course framework” that recognizes the critical need for long range vision and disease prevention, across all ages, but with a particular focus on early life and development when the foundation of all aspects of health and resilience are laid.

Box 1 **The purpose and goals of inVIVO Planetary Health**

Our purpose: we are a progressive scientific movement providing evidence, advocacy, and inspiration to align the interests and vitality of people, place, and planet.

Our goal: to transform personal and planetary health through awareness, attitudes and actions, and a deeper understanding of how all systems are interconnected and interdependent.

Detroit—a rejuvenating city, transforming after social, environmental, and economic devastation—provided a fitting backdrop for these discussions. Connections to the local area included case studies from inspirational local efforts to restore hope, purpose, and reduce health and social disparities through community youth activities, community-led art projects, environmental remediation, advocacy through legal action, free inner-city food farms, and sensory play gardens for children to play in. These examples, and many others, underscore the importance of local projects and community initiatives everywhere as part of a global interconnected grass-roots movement for social, racial, and environmental justice and planetary health.

Through our meetings, collaborations, and publications, we aim to create a global community for change-making and creativity, a forum to bring together diverse perspectives and expertise, and opportunities to influence and transform diverse global conversations, as well as promote the principles and practice of planetary health (Box 2) [2].

Box 2 **The inVIVO principles of Planetary Health**

We recognize that improving the health of all natural and anthropogenic systems depends on mutualistic values; planetary consciousness;advocacy; unity of purpose; recognition of biopsychosocial interdependence emotional bonds between people and the land; efforts to counter elitism, social dominance, and marginalization; meaningful cross-sectoral and cross-cultural narrative; self-awareness; and a personal commitment to shaping new normative attitudes and behaviors. This is the basis of the 10 inVIVO Principles for Planetary Health:
1. Sustainable vitality for all systems.6. Nature Relatedness.2. Values and Purpose.7. Biopsychosocial interdependence.3. Integration and Unity.8. Advocacy.4. Narrative Health.9. Planetary Consciousness.5. Countering elitism, social dominance, and marginalization.10. Personal commitment to shaping new normative behaviors.(From Prescott et al. *The Canmore Declaration*: Challenges
2018, 9(2), 31) [2].

**Figure 2:**
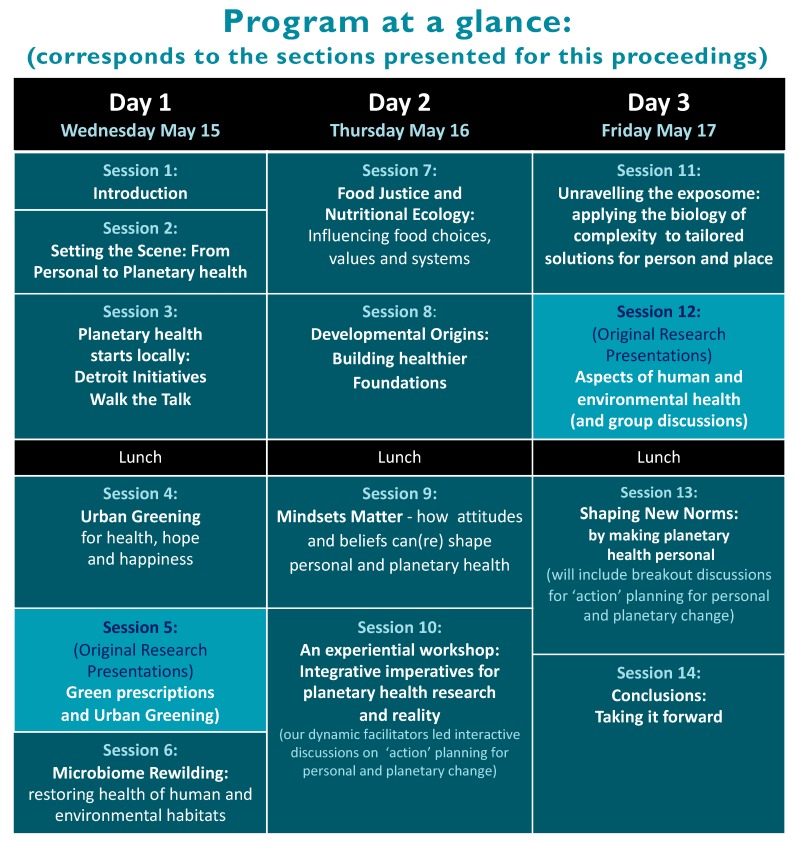
The Conference Program Overview: This shows the overarching structure of the meeting, demonstrating the flow and evolution of interdependent concepts as the meeting evolved. This structure is reflected in the order of the proceedings below (abstracts are presented from each of these sessions to provide further detail of the content).

Each of the following sections reflects the main sessions of the meeting, presented in order of proceedings, and includes the summaries and/or abstracts of each presenter for each topic discussed. Accordingly, the structure of this report reflects the meeting agenda as it evolved, divided into the sessions outlined in Figure 2.

## 2. Setting the Scene: From Personal to Planetary Health (Session Summary and Abstracts)

This session provided a series of platform talks to set the scene and contextualize the conference, framing the health of person, place, and planet with unifying concepts.

### 2.1. KEYNOTE: Beyond Science and Technology: Creating Planetary Health Needs Less ‘Head Stuff’, More Social Engagement and More ‘Heart, Gut and Spirit’ Stuff:

Trevor Hancock (Public Health Physician; retired Professor, School of Public Health and Social Policy (retired), University of Victoria, Victoria BC V8W 2Y2 Canada; First leader, Green Party of Canada. WHO consultant, co-creator of “Healthy Cities” program).

I have been involved in studying and working within what is now called the Anthropocene for almost 50 years, and in all that time, not only have we failed to make much progress, but the state of the Earth’s ecosystems has generally worsened. Yet somehow we must create a world in which everyone on Earth has good health and a good quality of life—a matter of social justice—while living within the physical and ecological constraints of the one small planet that is our home; this is the focus of the new field of planetary health. Our worsening situation is not due to lack of knowledge, science, and technology; in broad terms, we knew most of the challenges and many of the needed solutions back in the 1970s. Instead, the challenges we face are social, rooted in cultural values, political ideologies, legal and economic systems, ethical principles, and spiritual/religious beliefs. Therefore, we have to move beyond science and technology and address these broader socio-cultural issues by engaging in economic, legal, and political work, complementing and supplementing ‘head stuff’ with ‘heart, gut and spirit stuff’, and working from the grass roots up.

**Suggested reading** (for full paper on this topic): Trevor Hancock. Beyond Science and Technology: Creating Planetary Health Needs Not Just ‘Head Stuff’, but Social Engagement and ‘Heart, Gut and Spirit’ Stuff. Challenges 2019, 10(1), 31

### 2.2. KEYNOTE: Exiting the Anthropocene: Nature Based Solutions for Personal and Planetary Health

Matilda van den Bosch (University of British Columbia, Vancouver, BC V6T 1Z4, Canada; Editor, The Oxford Textbook of Nature and Public Health. Oxford University Press. Board member of the International Society of Doctors for the Environment)

Of late, the recognition of nature as a healthy living space has received increasing attention. A number of studies confirm that urban natural environments promote health and prevent disease. In order to incorporate such findings in sustainable city planning, the concept of Nature Based Solutions can be useful. This refers to actions that integrate human health benefits with environmental benefits—a necessary approach in times where we urgently need to realize that our health is totally dependent on the health of nature and ecosystems.

In research around the human health effects of nature exposure, several pathways have been analyzed. Studies suggest that neighborhood nature promotes physical activity and social connections and reduces stress. This is of particular importance since we know that physical inactivity, loneliness, and chronic stress are major risk factors contributing to the burden of non-communicable diseases.

Possibly even more importantly is that nature contributes to human health through ecosystem services (ESS). These are so called services that the natural environment provides to human beings by, for example, providing food, regulating the climate, and creating spaces for aesthetic and spiritual experiences. Our mere survival is dependent on these services. Given that biodiversity is the fundament of functional ESS, we need to start asking ourselves what will happen to the human species in the age of the Anthropocene, when the speed of biodiversity loss is constantly highlighted in news and through scientific studies.

Addressing this issue through a positive language, such as with the nature-based solutions concept, may be a way to start translating science into action. As researchers, we have often failed to communicate our findings, including those of the health benefits to be gained from nature. As human beings, we are prone to cognitive bias, and since the concept of ESS and nature based solutions is not yet part of the common agenda and mindset of policy makers, it is hard to make an impact. We also speak very different languages in different sectors, and the messages have a tendency to be unbalanced where we often stress risks associated with nature, such as pollen and vector-borne diseases. While these threats are indeed real, it is important to communicate that the benefits with nature far outweigh the risks, and it is human interference with nature that has set the system out of order and increased the occurrence of allergies and pathogens. To do this, we need to start using a bold and affirmative language that clearly articulates that the benefits of nature and that without it we simply have no chance of surviving on this planet. We must underscore that urban natural spaces benefit health by cultural and regulating ecosystem services. Evidence of health impact must be sufficiently implemented in urban planning, with more transdisciplinary actions to bridge the science policy gap.

**Suggested reading**: van den Bosch & Nieuwenhuijsen, 2016. No time to lose—Green the cities now. *Environment International.* 99:343–350

### 2.3. KEYNOTE: Microbiomes in transition: Impact of Climate Change on Microbial Systems in the Environment

Janet Jansson (Chief Scientist for Biology, US Dept Energy; Biological Sciences Division, Pacific Northwest National Laboratory, 902 Battelle Boulevard, Richland, WA, 99352, USA)

We live on a microbial planet. For example, each teaspoon of soil has billions of microbial cells, and most of these (>90%) have never been cultivated and their properties are unknown. This microbial universe (Earth’s Dark Matter) is responsible for many fundamental life processes on a global scale, and it is vital to understand how planetary changes impact the microbial functions that are essential for life. Examples of key ecosystem services carried out by soil microbial communities (microbiomes) that enable life on Earth include degrading organic carbon and pollutants, cycling nitrogen and other essential nutrients, and sustaining growth of plants. They also provide a rich resource of biodiversity and are a source of new antibiotics and other drugs.

These is growing evidence that Earth’s soil microbiomes are being impacted by climate change. Permafrost is an example of a vulnerable soil ecosystem that is thawing and contributing greenhouse gases to the atmosphere due to climate warming. Climate change is also resulting in shifts in precipitation patterns in otherwise highly productive agricultural soils, with increased flooding in some areas and drought in others. Drought in particular is predicted to become more prevalent in the future across large areas of the continental USA, with potentially negative implications for organic carbon cycling and facilitation of crop production through soil microbiomes.

These scenarios raise serious overarching questions: How does climate change impact soil microbiomes and the functions they carry out? What are the metabolic signatures of microbiomes in transition? How can knowledge of soil microorganisms be used to improve climate model predictions?

To answer these questions, we need to move beyond sequencing towards a more “functional” understanding. By sequencing we can obtain microbial genomes from soil and use this information to determine which microbes are present and their potential functions. However, in order to reveal what they are actually “doing” we need to move beyond genome-based understanding to phenotype information. The phenotype of a community, or "metaphenome", is the collective function of the system that is determined by the genetic potential in the community genomes and the environmental conditions that determine which genes are expressed. Currently the expressed genes, proteins, and metabolites involved in complex biogeochemical pathways carried out by soil microorganisms are largely unknown. The long-term goal and opportunity is to address this knowledge gap through use of advanced multi-omics, imaging, and other emerging technologies that will allow us to not only better understand the specific ecosystem services provided by soil microorganisms, but also equip us to use this knowledge to predict and control microbial metabolic routes for targeted endpoints.

Our next challenges include 1) better understanding of soil microbiomes and how they are impacted by local and planetary changes, 2) incorporation of process-scale knowledge of soil microbial functions to improve climate change predictions, and 3) use of this knowledge for novel solutions to mitigate negative consequences of climate change.

**Suggested reading**: JK Jansson, KS Hofmockel. The soil microbiome-from metagenomics to metaphenomics. Curr Opin Microbiol. 2018 Jun;43: 162–168.

National, Academies of Sciences, Engineering and Medicine 2019. Science Breakthroughs to Advance Food and Agricultural Research by 2030. Washington, DC: The National Academies Press. https://doi.org/10.17226/25059

JK Jansson, KS Hofmockel. Soil microbiomes and climate change. Nature Reviews Microbiol. 2019 (in press)

### 2.4. KEYNOTE: Systems Biology and the “Omics” Revolution: Connecting Personalized Lifestyle Medicine to Planetary Health

Jeffrey Bland (Founder and President of the Personalized Lifestyle Medicine Institute, Tacoma, WA 98443, USA; Founder and Chairman Emeritus of The Institute for Functional Medicine Tacoma, WA 98003)

In 1977 Richard Dawkins authored his now famous book The Selfish Gene. In this book, he advanced the concept that the purpose of the genetic code was to replicate itself. In this view of the gene, it has no interest in the organism beyond that of being a vehicle for its reproduction. The “selfish gene” concept, coupled with the genetic determinism of Mendel’s laws, both of which we presumably have little control over, has contributed to the development of the disease-centric health care system. In this system, the individual is disconnected from the environmental influences on how their genes are translated into how they look, act, and feel, and becomes the “patient” who is either genetically at risk of, or has, a specific disease.

The development of the “omics era” of the early 21st century has now exposed this conceptual framework of the health care system to be limiting in how to address the dominant health problems that are complex, such as chronic disorders including dysfunctions of the immune, neurological, gastrointestinal, endocrine, cardiovascular, and musculoskeletal systems that derive much of their origin from the interaction of the individual with their environment and lifestyle. The medical management of these conditions now constitutes more than 70% of the present health care expenditures in industrialized countries. According to the Center for Health Metrics and Evaluation, these same chronic health issues have now surpassed infectious diseases as the greatest cause of death and loss of quality life years.

Understanding the nature of this personal interface between an individual’s genes and their environment and lifestyle represents the future of medicine and the birth of precise, personalized health care. The early adoption of the concept of genomic-based care is in the field of personalized cancer therapy, which treats cancer as a cellular disease associated with a particular alteration in the genetic structure of the individual who requires individualized therapy. This form of cancer treatment is less focused on where the cancer is located in the body and more focused on what the nature of the unique genetic alteration is and the role of the tumor microenvironment of the individual, which gives rise to a personalized approach to therapy. We are witnessing this concept moving from the field of oncology to that of the prevention and treatment of complex, chronic diseases whose origin is rooted in the unique connection between the individual’s genes and their lifestyle.

The application of this concept to health care brings into focus the importance of the health of the individual being directly linked to systems health within the global environment.

## 3. Planetary Health Starts Locally: Detroit Initiatives Walk the Talk (Session Summary and Abstracts)

This session under-scored the importance of interconnected grass-roots efforts for planetary change, using many inspiring examples from the local Detroit community.

### 3.1. The Center for Urban Responses to Environmental Stressors (CURES)

Melissa Runge-Morris (Director CURES for cleaner and healthier living and working environments in Detroit; Institute of Environmental Health Sciences Wayne State University, Detroit, Michigan, 48202, USA)

The Center for Urban Responses to Environmental Stressors (CURES) (P30 ES020957) is an National Institute of Environmental Health Sciences (NIEHS)-funded environmental health sciences core center that is headquartered at Wayne State University in the heart of Detroit, Michigan. In partnership with academic researchers, clinicians, population scientists, educators, and members of the urban community, the CURES team works together to catalyze innovative research focused on the complex health impact of environmental exposure to chemical and non-chemical stressors throughout the human life-course. CURES programs are dynamic and integrative (Figure 3). Our ultimate goal is to create a healthy Detroit and to achieve environmental disease prevention through public policy. We understand that urban environmental chemical exposures are complicated and also dynamic. Environmental chemical exposures include complex mixtures as well as legacy and emerging contaminants such as lead contamination, persistent organic pollutants, phthalates, and airborne pollutants. We realize that our vulnerability to environmental chemicals may be different at different life-stages, and health consequences are also subject to non-chemical stressors such as socioeconomic drivers of adversity, violence, inequity, lack of green spaces, and other consequences of the built environment.

The CURES team recognizes the need to adopt a “big picture” approach to understanding of the diverse biological pathways in each of us that have the potential to lead either to disease or health resilience. In CURES, we share a commitment to perspectives that are central to the “developmental origins of health and disease (DOHaD)” concept. This is because DOHaD underscores the vital importance of a healthier start if we are to realize long term health. Our approach to disease prevention addresses these challenges on multiple levels including:

(a) **Biological pathways:** genetic, epigenetic, molecular, neuro-endocrine, detoxification, and inflammatory processes;
(b) **Cognitive pathways:** cognition and motivation, self-efficacy, cognitive development, and capacity;
(c) **Behavioral pathways:** physical activity, addictive substances, and nutrition.

Our cross-cutting approach is founded on bi-directional community communication, and includes basic and applied research, capacity building and career development for next generation leaders, research translation, and advocacy in policy that places an emphasis on prevention.

### 3.2. Community Partnerships for Co-Creating Change: Serving, Informing and Learning from Our Community

Brian D. Smith (Wayne State University, lifelong Detroiter and Army veteran, Community Relations Specialist for CURES, Wayne State University, Detroit, Michigan, 48202, USA).

The goal of the CURES Community Engagement initiative is disease prevention through awareness and public policy change. We foster collaborations and co-learning experiences for environmental health advocates, stakeholders, leaders, and researchers, serve as a resource for environmental health information and expertise, and promote environmental health awareness. We also advance the science of community engagement.

Fostering bi-directional communication is key to making meaningful change at the grass roots where it is more impactful for communities. This means dismantling the traditional academic approach of research largely led by institutions/scientists in which the community at-large is minimally engaged or not present at all. CURES embraces a new framework of participation in which community stakeholders fully participate and include the populations most impacted by the issues.

We take a more comprehensive and tailored approach to information dissemination, which varies according to the audience. We recognize that engagement is key to initiating successful interventions and policy change.

All our research and advocacy processes start with an environmental health issue defined by Detroiters. To this end, the CURES Community Engagement Core (CEC) works with Detroit residents through its Community Advisory Board (CAB) to evaluate and inform topics of relevance. Scientists, CEC, and CAB then collaborate and plan to determine information and resource needs for events, activities, and research. Information is disseminated through community-based events, video recorded, edited, disseminated in smaller venues, and supporting printed materials are created together.

Our future directions are towards further advancing science as a tool for advocacy to a) inform both communities and decision makers, b) support environmental justice movements with data and capacity (mini-grants), and c) train scientists and students. In all our efforts, we recognize that we must stay grounded in the needs of our community to succeed.

### 3.3. Creating a Sustainable, Fair, and Healthy Food System (Detroit Food Policy Council)

Winona Bynum (Executive Director, Detroit Food Policy Council, Detroit, MI 48226, USA).

Food Policy Councils (FPC) convene citizens and government officials for the purpose of providing a comprehensive examination of a state or local food system. This unique, non-partisan form of civic engagement brings together a diverse array of food system stakeholders to develop food and agriculture policy recommendations. In essence, FPCs provide important social infrastructure.

The Mission of the Detroit FPC is to influence policy which ensures the development and maintenance of a sustainable and equitable food system, resulting in a food-secure City of Detroit in which all of its residents are hunger-free, healthy, and benefiting from a robust food system.

Our vision is that the residents of the City of Detroit are educated about healthy food choices and understand their relationship to, and benefit from, policies that promote food security, food justice, and food sovereignty. We envision a city of Detroit that has a healthy, vibrant, hunger-free populace with easy access to affordable fresh produce and other healthy food choices. We see that urban agriculture and composting can operate sustainably and contribute to the City’s social and economic vitality.

Our values are those of justice, respect, integrity, inclusion, and transparency. We believe that residents, workers, and visitors should be treated with respect, justice, and dignity by those from whom they provide and obtain food.

We see many opportunities in the future including more land-based projects, greater community advocacy, more support for beginning new food initiatives, youth work, greater connection of people to resources, and food system education.

### 3.4. Make Food Not Waste

Danielle Todd (Director, Make Food Not Waste, Detroit, MI 48226, USA).

Make Food Not Waste is a community not-for-profit organization dedicated to reducing the amount of food that goes to waste in Southeast Michigan. Through public events, education outreach and community presentations, we give people the tools they need to waste less where they live and work. We recognize that food waste is a major environmental, financial, and social issue that has far reaching effects on all aspects of society.

In the U.S., we waste 40% of the food we grow and most of that happens at home. This has direct costs to households, with estimates that families of four can save anywhere from $1600–$2200 a year by not throwing as much food away. Furthermore, it has costs for our environment, not only through lost productivity and wasted resources, but also because food rotting in landfills adds to the burden of greenhouse gases.

Food waste reduction can be addressed at multiple levels, beginning where it is most effective and most desirable—reducing the volume of surplus food produced. Downstream strategies include redistributing extra food to people (soup kitchens and shelters), feeding animals, industrial uses, and composting to avoid the least desirable (but often default) outcome of excess food going to landfill or incineration.

Initiatives like Make Food Not Waste promote community awareness and practical strategies through food management education, food recovery support and composting program promotion, and many local events that inspire food waste prevention. This includes an annual community feast at Detroit’s Eastern Market. We encourage the public message that reducing food waste is “better for your budget, better for our community, and better for our planet” and provide the tools to do this.

### 3.5. Addressing Vacant Land in Detroit: Lessons for Health Equity in Legacy Cities

Natalie Sampson (University of Michigan-Dearborn, Dearborn, MI 48128, USA. Co-chair of American Public Health Association [APHA]’s Environmental Justice Subcommittee).

Many cities worldwide exhibit depopulation and disinvestment. In several U.S. cities, for instance, surges in residential vacancy have resulted largely from mortgage and property tax foreclosures related to historically discriminatory policy processes. As a result of diverse sociopolitical and economic forces, many legacy cities are characterized by neighborhoods with a high level of vacant structures and lots. A vacant property, which typically lacks maintenance, often contributes to negative mental health effects by eroding neighborhood social networks, reducing property values, and degrading nearby built environments, with increases in crime. To support human wellbeing in such neighborhoods, there is a need to understand implications of landscape change for residents, as well as their experiences and preferences. Further, decision-makers in legacy cities must also confront major issues including aging infrastructure, changing climates, and health inequities in their land use planning. This presentation will share lessons from various studies in Detroit, including a photovoice project that visually documented residents’ perceptions of vacant lots as they relate to health and safety; a qualitative study of residents experiencing health concerns from recurrent household flooding associated with disinvestment in infrastructure; and an overview of ‘NEW-GI’, a recent vacant lot greening effort to address stormwater management. In combination, these studies provide diverse methods to capture residents’ experiences and perceptions that are often overlooked or underappreciated in land use and infrastructure decision-making. Well-intentioned programs, plans, and policies may perpetuate disparities in health or safety when those most vulnerable are excluded from decision-making. These findings may encourage community leaders, planners, and policymakers across Detroit and other legacy cities to fully understand the nuanced opportunities and challenges of residential vacant land in ways that promote health equity.

### 3.6. Urban Planning for Transformation: At the Convergence of Empowerment, Creativity and Resources

Khalil Ligon (Lead Urban Planner, City of Detroit Planning and Development Department, Detroit, MI 48226, USA)

Through its neighborhood framework planning, the City of Detroit is pursuing an urban redevelopment strategy unlike any implemented in America. Community members and city officials collaborate to identify and prioritize needed improvements using innovative approaches to transform vacant parcels of land into community assets.

Acts of land stewardship and visible cues to care contribute to changes in physical and social environments and are aligned with various aspects of health. Both theoretical and practical applications of residents’ perspectives on landscape care have implications for wellbeing and neighborhood stability. Community redevelopment, blight remediation, and other landscape planning efforts may benefit from supporting acts of care and cues to care that promote health and neighborhood stability.

**Suggested reading:** Sampson, N., Nassauer, J., Schulz, A., Hurd, K., Dorman, C., & Ligon, K. (2017). Landscape care of urban vacant properties and implications for health and safety: Lessons from photovoice. *Health and Place,* 46, 219–228.

## 4. Urban Greening for Health, Hope and Happiness (Session Summary and Abstracts)

This session focused on the importance of nature-based interventions to restore urban habitats for multifaceted health benefits for individuals, environments, and societies on all levels.

### 4.1. KEYNOTE: Nature-Based Interventions for Disease, Violence and Injury Prevention in Urban Areas

Michelle Kondo (USDA Forest Service, Northern Research Station, Philadelphia, PA 19103, USA; acclaimed Scientist in urban health. She is renown for her place-based and nature-based interventions in low-resource urban communities).

Place matters. Nature is part of the “place” of where we live, and our “ZIP Code” may be as important as our genetic code in determining our long-term health and resilience. The qualities of the physical environment (such as quality of food and recreational resources, “grey spaces” versus natural “green spaces”, housing, and services) interact with the neighborhood social environment (safety, violence, social connections, social institutions, and normative values) to determine levels of stress, patterns of behavior, and, ultimately, the long term health of individuals and communities.

Many of these environmental determinants of health segregate by race and socioeconomic status, with obvious resource inequalities. Improving urban environments is therefore a public health priority.

Health outcomes are closely tied to urban nature exposure, and urban place-based and nature-based interventions have been shown to improve both public health and public safety. Collectively, studies have shown these strategies reduce all-cause mortality, post-operative recovery, biological markers of stress, mental health disorders, crime, and violence, and improve mood and attention. Increasing access to green space has also been shown to reduce the health disparities of social inequality.

Neighborhood interventions are therefore a growing strategy for improving health and social cohesion, including remediation of vacant properties which are associated with trash dumping, rodents, pathogens, illicit activity, and other adverse consequences including fear, anxiety, stress, and depression.

In Youngstown, Ohio we examined the impact of both contractor greening (“clean and green”) and community-led greening (“community reuse” initiatives such as community gardens) of vacant lots in a quasi-experimental design. We found significant reductions in burglaries and overall robberies, although there was an increase in motor vehicle theft. We also saw reductions in assaults and violent felonies, and spill-over crime-reduction effects into neighboring areas, especially with community reuse lots.

We extended this to a citywide cluster randomized trial in Philadelphia to restore blighted vacant land and examine the effects on violence, crime, and fear. In total, 541 vacant lots were randomly assigned to treatment and control. After a 38 month study period crime and violence data were examined in 445 participants. Significant reductions in crime overall (−13%), gun violence (−29%), burglary (−22%), and nuisances (−30%) were seen in neighborhoods below the poverty line. There were also significant reductions in perceptions of crime, vandalism, and safety concerns, and significantly increased use of outside spaces for relaxing and socializing.

Greening vacant land also had benefits on mental health outcomes. In total, 442 participants were surveyed before and after intervention. There was a significant decrease in depression and feeling of worthlessness for participants living near treatment lots, but no change in feeling nervous, hopeless, or restless. We have also observed vacant-lot clean and green impact biological markers of stress in nearby residents, including a significant drop in heart rate (marker of acute stress) when walking in view of newly greened vacant lots.

Collectively, these findings indicate that everyday environments can influence health and safety, and that interventions to improve environments provide a significant return on investment. Vacant lots typically cost $1600, $180/year maintenance, with $26 in net benefits to taxpayers and $333 to society at large, for every dollar invested. On the other hand, failing to remediate environments has long-term adverse effects. Toxic neighborhood environments independently predict intergenerational social mobility of black and white children. “Neighborhood toxicity” is a stronger predictor than poverty of lower income mobility, and higher rates of teenage birth and incarceration as an adult. This is linked with high rates of violence, incarceration, and lead exposure. Black children are disproportionately affected. Thus, improving urban environments should have far greater emphasis in public health policy.

**Suggested reading**: Kondo, Fluehr, McKeon, Branas (2018). Urban Green Space and its Impact on Human Health. *International Journal of Environmental Research and Public Health* 15(3): 445.

Kondo, Andreyeva, South, MacDonald, Branas (2018). Neighborhood Interventions to Reduce Violence. *Annual Reviews of Public Health* 39, 253–271.

Kondo, Hohl, Han, Branas (2016). Effects of Greening and Community Reuse of Vacant Lots on Crime. *Urban Studies* 53(15): 3279–3295.

Branas, South, Kondo, Hohl, Bourgois, Wiebe, MacDonald (2018). Citywide cluster randomized trial to restore blighted vacant land and its effects on violence, crime and fear. *Proceedings of the National Academies of Science* 115(12): 2946–2951.

South, Hohl, Kondo, MacDonald, & Branas (2018). Effect of Greening Vacant Land on Mental Health of Community-Dwelling Adults: A Cluster Randomized Trial. *JAMA Network Open, 1*(3), e180298–e180298.

### 4.2. Nature Relatedness as a Basic Human Psychological Need

Daniel E. Baxter (School of Psychology, University of Ottawa, Ottawa ON, Canada K1N 6N5; investigates antecedents, motivation and consequences of pro-environmental behaviours, social dilemmas and resource sharing, nature relatedness, environmental meaning and age-friendliness)

Modern concepts of Nature Relatedness and “biophilia” were originally built on the fact that the evolution of human sensory, cognitive, and emotional apparatuses all would have occurred in the context of natural settings, and as such, they should be more readily attuned to natural stimuli from such environments. This has given rise to several other theories including the “Stress Reduction Theory” which has proposed that landscapes and environments that would have been optimal for human survival would have elicited a psychophysiological response, with diminished physiological arousal (lower heart rate, blood pressure, cortisol), reduced negative mood, and increased positive emotions and thoughts (feelings of vitality, optimism, and wellbeing). Others proposed the “Attention Restoration Theory”, that being in nature restores our capacity for directed attention, because humans find natural stimuli intrinsically interesting, and so by not having to evoke directed attentional capacity, we allow such capacity to become ‘recharged’.

More recently, we defined nature relatedness as “a basic human psychological need to feel a secure and pleasant experiential connection to nature in a cognitive, emotional and physical sense” (Baxter and Pelletier, 2019, p. 22; see suggested reading), and explored this in a two-process model examining:

- “Needs as motives”—built on earlier drive theories that considered a need to be a motivation toward a certain incentive or goal, directed by a deficit, behavior meant to satisfy the need, and then satiation;
- “Needs as requirements”—built on humanistic tradition, views a psychological need as an essential experiential nutriment to the optimal growth and wellbeing.

We then examined the evidence which demonstrate that nature relatedness meets the criteria constituting a basic human psychological need as described by Beaumeister and Leary, 1995 (see suggested reading). Full references to support these points are available in Baxter and Pelletier 2019 (below):

**First, there are affective consequences.** To be considered a basic need, there should be positive affective consequences when satisfied, and negative affective consequences when thwarted. In support of this there is good evidence that taking a walk in a natural area, or even simply viewing scenes of nature, has been consistently associated with increased positive affect compared to urban environments. Exposure to nature has been consistently associated with decreased negative affect over and above what simple relaxation can account for. In contrast, walking in urban environments can increase such negative affect. Evidence also suggests that the relationship between immersion in nature and changes in affect can be attributed to individuals’ level of perceived connection to the natural world.

**Second, there are benefits to health and wellbeing.** A basic psychological need also demands that health, adjustment, or wellbeing requires its satisfaction. Accordingly, being in nature is associated with lowered diastolic blood pressure, increased parasympathetic activity, decreased sympathetic arousal, and lower heart rate. Patients recovering from surgery whose window faced onto nature recovered faster, needed less pain management, had less complaints than identical patients whose window faced a brick wall. People who are stressed will recover faster when viewing nature scenes compared to urban ones, experience less autonomic arousal during a stressful video after viewing nature scenes compared to urban ones and are better insulated to further stressors. People who walk in nature (compared to urban areas), and people who feel more connected to nature report increased happiness, life-satisfaction, vitality, and psychological wellbeing and feel more capable of dealing with life stressors and dilemmas.

**Third, there are consequences when thwarted.** Failure to satisfy a need should result in maladaptive or pathological behavioral, psychological, and health outcomes. This is partly evidenced by nature walk vs. urban walk studies already discussed in previous criteria (affective consequences, promotion of health). Further to this, children raised in urban as opposed to rural environments tend to have higher prevalence of somatic complaints, internalizing problems, social withdrawal, delinquency, aggression, mood and emotional disorders, self-harming, dysfunctional communication and thought processes, adjustment disorder, reactive attachment problems, and ADHD. Adults living in proximity to even just 10% less green space within 1km have higher prevalence of coronary heart disease, depression, anxiety disorders, upper respiratory tract infections, asthma, infectious diseases of the intestinal canal, diabetes, and mortality rates for overall mortality and circulatory disease.

**Fourth, we can demonstrate universality.** The proposed need should not be culturally dependent. Accordingly, research findings have been found in a variety of cultures/countries.

**Fifth, this is not a derivate of other needs.** A new basic psychological need should not be derivative of another known psychological need. Accordingly, nature relatedness has been shown to predict wellbeing above and beyond overall feelings of connectedness, such as connection to family, country, culture, and general interpersonal connectedness. Exposure to nature accounts for positive benefits above and beyond what social contact predicts. In fact, people prefer walking alone in nature and will predict themselves to receive less restorative effect from nature if company is present but prefer to walk with company in urban environments. Being in nature accounts for wellbeing effects above and beyond the effect of physical activity.

**Sixth, this directs cognition.** A proposed need should direct cognitive activity, such as being categorized separately from other environments, processing information about nature differently, and blurring lines between nature and one’s self-concept. Accordingly, people very easily categorize natural stimuli separate from man-made stimuli, and that this actually happens even before people identify what specific environment it is, such as mountains vs. a lake vs. a forest. People attribute different qualities to nature, hold different attitudes toward nature, and have differential behavioral and psychological expectations for nature compared to other environments. Finally, people also internalize nature into the self-concept.

**Seventh, this affects a variety of behaviors.** A basic psychological need should affect a wide variety of behaviors in order to be considered important enough to qualify as a need. Accordingly, nature relatedness is predictive of pet ownership, choice of food purchasing, involvement in environmental organizations, eating habits (e.g., vegetarianism), and participating in nature activities. Including nature in one’s self-concept predicts how one uses energy and treats their waste. Exposure to nature scenes causes people to be more cooperative over finite resources, and to show greater altruism and generosity towards others.

**Eighth, it occurs in a variety of settings**. A basic psychological need should not be restricted to just certain settings. Studies have used many different types of natural stimuli such as window views, photographs of nature, open-concept virtual reality, and actual walks through nature. Stimuli depict a variety of different environments, such as simple view of trees, a tree-lined footpath alongside a river, an arboretum, a nature preserve, fields and lowlands in a canyon valley, brushy mixed boreal forest, a hike in low mountains, and various scenes of lakes, rivers, hills and forests. A variety of urban settings as comparisons, such as a view of a brick wall, street-side views along a river, mixed residential and commercial areas, downtown centers, and large cityscape vistas was also used. Even when stimuli are matched in terms of how aesthetically pleasing they are, there is more restoration following a nature walk.

**Finally, it elicits goal-directed behavior.** A need should elicit goal-directed behavior that is meant to satisfy the need and is then subject to satiation. At least in part, these criteria can be evidenced from the activities that people pay attention to and engage in to fulfill the need, as discussed throughout all the previous criteria. Office workers in Norway who do not have a window view are five times more likely to personalize their office with plants and three times more likely to have pictures of nature. Attentionally fatigued participants have an increased positive attitude toward walking in nature, and a decreased positive attitude towards walking in urban environments.

In summary, establishing nature relatedness as a need puts it into a comprehensive theoretical framework by which future research can have a much more guided approach, and from which we can derive specific hypotheses. It highlights the importance of including as much nature as possible in urban design. It also highlights the importance of preserving as much pristine natural areas as possible with high biodiversity. It also dramatically helps to put into perspective that human beings are not separate from the natural world. To harm our natural environmental surroundings is as much a destruction of ourselves as it is of the physical world around us.

**Suggested Reading:** D. Baxter and L. Pelletier. (2019). Is nature relatedness a basic psychological need? A critical examination of the extant literature. *Canadian Psychology*, *60*, 21–34.

R. Baumeister, M. Leary (1995). The need to belong: desire for interpersonal attachments as a fundamental human motivation. *Psychological Bulletin*, *117*, 497–529.

### 4.3. Urban Greening and the Pursuit of Health Equity and Social Cohesion

Viniece Jennings (Senior Fellow in the Environmental Leadership Program. She has led work on urban green space, health disparities and social determinants of health. In 2017, she was selected as a “Hidden Treasure’ for Women of Excellence in Science by a foundation in metro Atlanta)

Urban green spaces can enhance the social environment and health of “people, place, purpose and planet”. While different aspects of social cohesion have been discussed in the literature, this presentation offers a new conceptual framework to illustrate the inextricable linked between the presence of green spaces, cultural ecosystem services and social dimensions of health and wellbeing, which all converge to influence health equity and social disparities. This required a holistic definition of health, which reconsiders the health of people within the contextual conditions of the places where we live, work, and play (i.e., social determinants of health), and encourages a place-based approach to health promotion. This approach also recognizes the value of residential satisfaction, access to other amenities that support a favorable quality of life and pathways to civic engagement for greater personal and community wellbeing. For example, the value of volunteer activity was also highlighted.

Giving greater focus and value on social cohesion (values and norms that support trust and wellbeing) and social capital (resources obtained from social relationships), the role of mediators that may lead to positive outcomes were discussed, including:

(a) sense of place,
(b) sense of purpose,
(c) social support and belonging, and
(d) empowerment through engagement and feeling of value.

At the personal level this may translate into physical, behavioral, and psychological responses that support increased physical activity, reduced stress, immune function, and individual subjective wellbeing. Opportunities can arise to potentially increase collective wellbeing at the community level. For example, planting programs that have a social mission, community oriented, and support local capacity to maintain trees can help address inequities more effectively. At the planetary scale this encourages us to reimagine how we characterize ecosystem services more generally and understand the overlap between ecological and sociological systems.

**Suggested reading:** Jennings, V., & Bamkole, O. (2019). The Relationship between Social Cohesion and Urban Green Space: An Avenue for Health Promotion. *International journal of environmental research and public health*, 16(3),

Jennings, V., Larson, L., & Yun, J. (2016). Advancing sustainability through urban green space: cultural ecosystem services, equity, and social determinants of health. *International Journal of environmental research and public health*, 13(2), 196.

Smiley, K. T., Sharma, T., Steinberg, A., Hodges-Copple, S., Jacobson, E., & Matveeva, L. (2016). More inclusive parks planning: Park quality and preferences for park access and amenities. *Environmental Justice*, 9(1), 1-7.

### 4.4. The Environment–Microbiome–Health Axis in Landscape Research: Disciplinary Crossovers and New Opportunities

RobinsonJake^1^,^2^,^3^,^4^(graduate student already with an international reputation for nature-based research; early career researcher on the inVIVO Board of Directors).1Department of Landscape, University of Sheffield, Sheffield S10 2TN, UK2Improving Wellbeing through Urban Nature (IWUN) Research Group, Sheffield S10 2TN, UK3In VIVO Planetary Health, Worldwide Universities Network (WUN), West New York, NJ 10704, USA4Healthy Urban Microbiome Initiative (HUMI), Adelaide, SA 5005, Australia

Humans are spending less time in biodiverse environments and according to the Old Friends and Biodiversity hypotheses, this has led to fewer interactions with diverse, immunoregulatory microorganisms or “old friends”. Furthermore, noncommunicable diseases are on the rise, which may be attributed in part to the breakdown of this evolutionary relationship between humans and environmental microbiota.

Building upon the Old Friends and Biodiversity hypotheses, there is growing interest in the environment–health–microbiome axis as a mechanism to explain some of the health benefits linked to spending time in natural environments. This provides a platform for proposing a new, holistic, and transdisciplinary approach to public and environmental health. Numerous disciplines including the constantly-evolving discipline of landscape research (in response to emerging socio-ecological issues) can make a significant contribution towards this approach, and both transdisciplinarity and innovation will play important roles in this process.

With recent advances in technology including more efficient DNA sequencing and state of the art remote sensing/3D modelling technology, there is an important opportunity to use this disciplinary crossover to gain important insights into the environment–microbiome–health axis. By joining disciplines and combining technologies we can better understand and visualize the structure, distribution, and functional roles and relationships of microbial communities within and across different landscapes and between hosts, and focus on their importance for people, place, and nature.

We now have an opportunity to pioneer a new field of study recently termed the Microbioscape.

Microbioscape research can add an important dimension to landscape literacy—the ability to ‘read’ and interpret landscape functions and characteristics. An example of a new Microbioscape project is 4D Microbial Cartography. In this project we are using unmanned aerial vehicles, also known as ‘drones’, to create 3D models of English oak trees *Quercus robur*, and then sampling their microbiomes across space and time (4D) to map and visualize various spatiotemporal dynamics. The interactive models will provide ecologists and public health professionals with an innovative tool to enable the integration of future microbiome research within designs for urban greening. There are many other ways in which these disciplines can combine to generate important benefits for planetary health. For example, in nature-based interventions, innovation green infrastructural designs, and creative practices for science communication—these will be highlighted and explored in this talk.

**Suggested Reading:** Jake M. Robinson, Jacob G. and Martin F. Breed. Walking Ecosystems in Microbiome-Inspired Green Infrastructure: An Ecological Perspective on Enhancing Personal and Planetary Health Challenges 2018, 9(2), 40

Kapono, C.A., Morton, J.T., Bouslimani, A., Melnik, A.V., Orlinsky, K., Knaan, T.L., Garg, N., Vázquez-Baeza, Y., Protsyuk, I., Janssen, S. and Zhu, Q., 2018. Creating a 3D microbial and chemical snapshot of a human habitat. *Scientific reports*, 8(1), p. 3669.

### 4.5. The Human Postmortem Microbiome, Neighborhood Blight and ‘Greening’ in Detroit

PearsonAmber^1^,^2^,^3^(health geographer with research interests in social justice and understanding the unexpected tenacity, adaptability and resilience of the underprivileged)PechalJennifer L.^4^SchmidtCarl J.^5^,^6^JordanHeather R.^7^BenbowM. Eric^4^,^8^1Department of Geography, Environment and Spatial Sciences, Michigan State University, East Lansing, Michigan, USA2Department of Public Health, University of Otago, Wellington, New Zealand3Environmental Science and Policy Program, Michigan State University, Michigan, USA4Department of Entomology, Michigan State University, East Lansing, Michigan, USA5Wayne County Medical Examiner’s Office, Detroit, Michigan, USA6Department of Pathology, University of Michigan, Ann Arbor, Michigan, USA7Department of Biology, Mississippi State University, Mississippi State, Mississippi, USA8Department of Osteopathic Medical Specialties, Michigan State University, East Lansing, Michigan, USA

The microbiome is important in human health, yet its connection to the broader environment remains understudied. We know little about how neighborhood conditions might influence the bacterial and archaea communities which live in and on the human body, or the microbiome. Taking advantage of a unique opportunity to collaborate with the Wayne County’s Medical Examiner’s Office, we tested relationships between features of the urban blight (e.g., abandoned buildings) and ‘greening’ efforts (e.g., tree plantings) using parcel data in Detroit, MI, and then compared neighborhood conditions to the composition and diversity of the human postmortem microbiome for five anatomical regions (ears, eyes, nose, mouth, and rectum). We observed significant clustering of microbial composition by blight high versus low neighborhood blight. Microbial biodiversity was significantly, positively correlated with neighborhood greening efforts while negatively correlated with blight. These results provide initial evidence of a relationship between both the composition and diversity of the human microbiome and neighborhood conditions, establishing the foundation for novel research into the pathways through which greening efforts and urban blight may influence health.

## 5. Green Prescriptions and Urban Greening (Original Research Presentations) 

In this session researchers presented their original research papers on this topic, followed by group discussions. This was an important opportunity for many students and early career researchers to share their work.

### 5.1. Nature Exposure, Connectedness with Nature, and Pro-Environmental Behaviour: How are They Related?

ClitherowTheodore J.^1^WhiteMathew P.^1^CartwrightBenjamin D. S.^1^HuntAnne D.^2^LeyshonCatherine^3^1European Centre for Environment and Human Health (ECEHH), University of Exeter Medical School, Knowledge Spa, Royal Cornwall Hospital Treliske, Truro, Cornwall, TR1 3HD, UK2Natural Solutions (c/o A D Hunt Ltd), Swindon, Wiltshire, SN26 7BQ, UK3Centre for Geography, Environment, and Society, University of Exeter, Penryn Campus, Treliever Road, Penryn, Cornwall, TR10 9FE, UK

A small body of research suggests that: a) exposure to natural environments; and b) connectedness with nature (CWN), are both positively associated with pro-environmental behaviors, but it is unclear how these processes interact. The current study used survey data from 360 adults in England to explore how CWN might mediate and moderate the relationship between nature exposure and pro-environmental behavior. First, results showed that whilst nature exposure and CWN were independent predictors of greater pro-environmental behavior, CWN was the strongest of these measures. The relationship between recalled childhood nature exposure and pro-environmental behavior in adulthood was significantly mediated by CWN. Additionally, CWN significantly moderated the relationship between “nearby” (neighborhood) nature exposure in adulthood and childhood nature exposure, and pro-environmental behavior. In contrast, CWN did not moderate the association between nature visit frequency in the last week and pro-environmental behavior. Results support and extend previous work by suggesting that people with higher levels of CWN tend to act more pro-environmentally regardless of their exposure to nature in childhood or their exposure to nearby nature in adulthood, whereas the pro-environmental behavior of those with lower CWN appears more sensitive to these types of nature exposure. These findings highlight the need to better understand both nature exposure and CWN in order to more fully appreciate their implications for policy and practice in supporting pro-environmental behavior.

### 5.2. Visual Stimulation with Natural Scenes is Relaxing, but Only in Mindful People: A Randomized, Crossover Trial and Brain Imaging Study with fNIRS

ChenChongTakaoAkiyoNakagawaErikaKobayashiAyumiFujiiYukoHirataKeikoMatsubaraToshioHagiwaraKousukeHashimotoAkikoHaradaKenichiroYamagataHirotakaSasakiJunHiguchiFumihiroNakagawaShinDivision of Neuropsychiatry, Department of Neuroscience, Yamaguchi University Graduate School of Medicine, Yamaguchi 755-8505, Japan

Due to increasing mental health issues and the compromised efficacy of pharmacological treatment, the development of complementary/alternative and preventive strategies is becoming a focus of interest. One such strategy is nature therapy. Contact with nature or merely viewing natural scenes has been reported to be healing and benefit the mind and brain. Yet, on one hand, whether nature therapy is effective for all people remains inconclusive, on the other hand, the underlying neurobiological mechanism of the therapeutic effect remains unclear. Therefore, in the present study, we tested the relaxing effect of viewing natural scenes with a randomized, crossover trial and explored its moderating factors as well as the underlying neural substrates using functional near-infrared spectroscopy (fNIRS).

The study was approved by the Institutional Review Board of Yamaguchi University Hospital and written informed consent was obtained from all subjects. Thirty healthy volunteers were randomly assigned to view pictures of nature or cities on two different days in a counter-balanced order. While viewing the pictures, their brain activities were monitored by fNIRS. Before and after the intervention, they were asked to report their feelings of relaxation, calmness, and stress on a visual analog scale (VAS).

We found that viewing pictures of nature but not cities had a significant relaxing (paired *t*-test, *p* < 0.05) and calming (*p* < 0.05) effect. Using repeated measures ANOVA controlling covariates including gender, age, profession, working memory, mindfulness, and the level of depressive and anxious symptoms, we found that the main effect of viewing pictures of nature on relaxation and calmness disappeared, while its interaction with mindfulness remained significant for relaxation (*p* < 0.05). Further analysis confirmed that the relaxing effect of viewing pictures of nature was significant only in subjects with high than average level of mindfulness (*n* = 13, *p* < 0.05). Meanwhile, partial correlational analysis controlling covariates indicated a significant correlation between mindfulness and the increase in feelings of relaxation after viewing pictures of nature (r = 0.562, *p* < 0.05). Probing the underlying neural substrates, we found that as subjects’ level of mindfulness became higher, their brain activation in response to nature scenes became lower in the right dorsolateral prefrontal cortex (dlPFC, r = 0.731, *p* < 0.05) and lateral orbitofrontal cortex (lOFC, r = 0.534, *p* < 0.05).

These results suggest that viewing pictures of natural scenes is relaxing only in mindful people and the dlPFC and lOFC may be involved in this moderating effect. As patients with depression typically show hyperactivation in dlPFC and lOFC at rest due to their ruminative tendency, nature therapy may be a promising treatment for depression. However, as suggested by the results here, mindfulness training should be prescribed together in order to achieve therapeutic effects.

### 5.3. Greening the Grey: How Do Front Gardens Impact Health and Wellbeing?

Chalmin-PuiLauriane Suyin^1^GriffithsAlistair^2^RoeJenny^3^CameronRoss^1^1University of Sheffield, Sheffield S10 2TN, UK2Royal Horticultural Society, London SW1P 2PE, UK3University of Virginia, Charlottesville, VA 22904, USA

While evidence grows for the biopsychosocial interdependence of green spaces and human wellbeing, front gardens in the UK are increasingly being paved over. This research investigates whether introducing plants to impermeable front gardens improves residents’ health and wellbeing. Although small in size and often publicly inaccessible, domestic gardens represent over 25% of UK urban green space and are the most readily accessible green spaces for residents. Front gardens bridge domestic and public realms, playing a role in shaping both outward and inward sense of place. Front gardens hereby become physically demarcated places through which to explore the relationship between human and planetary health.

Through a front garden greening intervention in a street of a deprived suburb in Northern England, we build on “Attention Restoration Theory” and “Stress Reduction Theory” to provide a basis on which to value these liminal spaces of urban nature in terms of their sociocultural impacts. The interdisciplinary approach includes a horticultural intervention alongside pre/post-intervention testing using questionnaires, in-depth interviews, and physiological measures of salivary cortisol. Participants’ (n = 42) front gardens were planted with *Petunia Surfinia* Sky Blue (petunia), Viola ‘Sorbet series’ (viola), *Rosmarinus officinalis Prostratus* (rosemary), *Lavandula angustifolia* ‘Hidcote’ (lavender), Rhododendron ‘Wombat’ (azalea), *Clematis alpina* ‘Jackmanii’ and ‘Ville de Lyon’ (clematis), *Galanthus nivalis* ‘Flore Pleno’ (snowdrop), Narcissus ‘Tête-à-tête’ (daffodil), *Crocus sativus* (crocus), *Amelanchier canadensis* ‘Glenn Form’ (serviceberry), and *Juniperus scopulorum* ‘Blue Arrow’ (juniper). Residents’ wellbeing was monitored over the course of a year following the planting.

Results indicate that perceived stress decreased following the introduction of colourful, planted containers and small trees in front gardens. Participants also reported heightened moods, motivation, relaxation, and pride of place. The intervention has not had any negative consequences for residents nor the area. Finally, we discuss the merits and challenges of fieldwork with a disadvantaged community, as well as potential opportunities for this type of low-cost, small-scale, modular, urban green infrastructure to be replicated elsewhere.

### 5.4. Developing Urban Greenness Exposure Metrics for Healthy Cities

NesbittLorien^1^AndreaniMatteo^1^JarvisIngrid^1^LiXiaojiang^2^RattiCarlo^2^SeiferlingIan^2^VilleneuvePaul^3^van den BoschMatilda^1^1University of British Columbia, Vancouver, BC V6T 1Z4, Canada2Massachusetts Institute of Technology, Cambridge, MA 02139, USA3Carleton University, Ottawa, ON K1S 5B6, Canada

**Introduction:** Exposure to urban vegetation has several health benefits, yet interpretation is limited by uncertainty about the accuracy of greenness exposure metrics, often derived from different remotely-sensed data sources and using different spatial methods. This research assesses the strengths and weaknesses of multiple greenness data sources and spatial metrics for application in environmental health research, particularly related to climate change and extreme heat, in order to develop an alternative greenness exposure metric that can be applied worldwide.

**Methods:** We analyzed 4 data sources: Landsat 8, Sentinel-2, RapidEye, and the Green View Index (GVI), derived from Google Street View. These data sets span various resolutions and costs and represent aerial and novel perspective views. We compared the Normalized Difference Vegetation Index (NDVI) using different imagery resolutions and the GVI for multiple buffer distances around postal codes, and examined sensitivity to spatial metrics and data sources, using Pearson’s correlations and linear and spatial regression models.

**Results:** NDVI was highly correlated among satellite types, although small-scale geographical variations were observed. GVI showed lower correlations with all other data types, suggesting an important new source of greenness data. Greenness measures were spatially sensitive, and GVI showed different spatial relationships with temperature than NDVI measures, highlighting the importance of accurately estimating spatial exposures.

**Conclusions:** This research is the first comparison of multiple remote sensing data sources and metrics, including both aerial and novel perspective views. It presents alternative greenness exposure metrics and informs climate adaptation approaches using freely accessible data sources that may be applied internationally.

### 5.5. The Urban Community Agriculture Practices in Chinese Cities: A Case Studies in Nanjing

WangYifanZhangJ. R.ZhouZ.Q.LiuJ. P.XuQ. X.XuZ.Nanjing Forestry University, Xuanwu Qu, Nanjing Shi 210037, Jiangsu Sheng, China

**Introduction:** Nurturing plants and reaping the harvest is an intuitive yet civilized activity, yet seemed unpractical and inconclusive for metropolis dwellers in China, especially from the 1990s to the 2000s. However, urban farmers are changing the public life in a formal and informal way with food as the medium as well as the catalyst over the last decade in China. This paper aims to unravel the context and highlight the potential value of urban agriculture.

**Methods:** With questionnaire and ethnographic observation, the authors investigated three groups of people: green community activists, pioneering urban gardeners, and community agriculture charities who are committed to building a more socially and economically sustainable system.

**Results:** The remodeling of public life has become a public issue, with the efforts of green community activists attracting media and environmentalist attention. They are the forerunners of urban agriculture; pioneering urban gardeners play the role of practitioners. As long as there is vacant land, they will always grow vegetables spontaneously. Community agriculture charities set up fields in the community, allowing residents to plant for free. They provide the underlying platform for urban agriculture. These three groups resort to NGOs, volunteers, and institutions to collaborate with government departments to streamline the procedure of public participation in shaping the neighborhoods. Such efforts are expected to provide nostrum for urban resident. Currently, Chinese people are confronted degrading traditional family and neighborhood relationships, as well as an alien natural environment. Therefore, rebuilding the community and public space has become a critical issue in China.

**Discussions:** Along with a different economy to western countries, China has a different land property system and community management, which generally hinders development of community garden. Since the 2010s, some new measures have been taken in certain neighborhoods to enhance public involvement. Urban agriculture is integral to urban development strategy in China. The investigations are contextualized in such policy vicissitudes to give hints of complexities and uncertainties of food ideals.

### 5.6. Noticing Urban Nature, Mental Health and University Students: Does 7 Days Make a Difference?

BoydFrancesca^1^,^2^1Department of Landscape, University of Sheffield, Sheffield S10 2TN, UK2Improving Wellbeing through Urban Nature (IWUN) Research Group, Sheffield S10 2TN, UK

University student support services have experienced continued increase demand for mental health services. A higher percentage of students report experiencing a mental health issue than the general public (Universities UK, 2018). Are there preventative measures which could be implemented to support this population? With the known benefit of nature on health, wellbeing, and pro-environmental attitudes, are there simple and effective ways to influence students’ connection to nature? Can a 7 day green intervention make a difference?

This presentation offers the findings of my PhD research, exploring the impact of two 7 day interventions for 18–24 year old university students. The research compares two interventions: a specially designed mobile app (Shmapped) and a group walk. This presentation gives an overview to the Improving Wellbeing through Urban Nature project, before delving into the details of my personal research within the context of the current challenges experienced by some university students in the UK. It then evaluates possible opportunities to implement preventative measures and intervention known as green prescriptions.

To finish, the results from this research are presented as a holistic overview. Quantitative analysis is entwined with personal stories to provide answers to questions of nature connection intervention effectiveness, the influence of ‘7 days’ on wellbeing and the role of tailoring interventions to the individual. Statistical analysis and focus group findings in this research offers an insight on the influence of urban green spaces on university campuses and young adults’ daily nature interactions.

### 5.7. To Biophilia and Beyond: Role of Biodiversity and Urban Nature and Walkability for Ageing-in-Place in Belfas 

MacCarthyDanielleEllisG.AdlakhaD.Queen’s University, Belfast BT7 1NN, UK

The impact of industrial 19th and 20th century urbanization has left contemporary cities with diminishing sites of urban nature, habitat fragmentation, biodiversity loss, and a deep fissure in the human–nature relationship. Furthermore, while prioritizing the place for nature within our cities is well-evidenced to benefit both human and planetary health, normative health models in urban planning continue to place emphases on built characteristics with little mention of the role of biodiversity for human health or planetary co-benefits. This paper explores findings from the first phase of a mixed method research design which takes normative urban planning models of walkability—neighborhood built environment characteristics, higher residential density, land use mix, street connectivity, aesthetics, and safety shown to affect more minutes of physical activity and better mental wellbeing—to establish a basis to explore the inclusion of a biodiversity indices within the walkability index framework.

Research on the influence of the biodiversity neighborhood environment within the ageing-in-place discourse, in particular, is limited. We analyze data from 150 older age adults obtained from the HULAP study (Healthy Urban Living Ageing Places) including GPS pedometer data, GIS data, and mental health survey data across high and low walkable neighborhoods against a biodiversity index and report on the statistical results of exposure and time spent in richer biodiverse neighborhoods on physical activity and mental wellbeing.

This study constitutes the quantitative phase as part of a larger study examining the role of biodiversity and urban nature for ageing-in-place strategies, with implications for both human and non-human ecosystems, seeing the interconnectedness as vital and critical.

### 5.8. Urban Greening: Geographical Differences in Aeroallergen Concentrations

Sierra-HerediaCecilia^1^AllenRyan^1^HendersonSarah B.^2^,^3^CoatesFrances^4^BrubacherJordan^1^LavigneÉric^5^BrookJeffrey R.^6^,^7^TakaroTim K.^1^1Faculty of Health Sciences, Simon Fraser University, Burnaby BC V5A 1S6, Canada2BC Centre for Disease Control, Vancouver BC V5Z 4R4, Canada3School of Population and Public Health, University of British Columbia, Vancouver BC V6T 1Z3, Canada4Aerobiology Research Laboratories, Ottawa ON K2E 7Y5, Canada5School of Epidemiology and Public Health, University of Ottawa, Ottawa ON K1N 6N5, Canada6Air Quality Research Division, Environment Canada, Toronto ON M3H 5T4, Canada7Dalla Lana School of Public Health, University of Toronto, Toronto ON M5T 3M7, Canada

Climate-related changes in the local ecosystem’s conditions will present specific threats for their human population because it has impacts in nearly every area of human interactions, and many of these impacts are interrelated. All of these threats and impacts will, according to the IPCC (Intergovernmental Panel on Climate Change) result in “greater likelihood of injury, disease, and death”.

Among the areas of human life affected by climate change, health is an immediate and prominent one. The incidence, range and seasonality of many existing disorders will certainly be altered due to their connection with the fluctuations in the average climate conditions, the climatic variability, and other noninfectious drivers of health.

Over the past 70 years, the prevalence of allergic conditions has increased in high-income countries to the point of becoming a worldwide public health concern. Environmental exposures to aeroallergens and air pollutants have been hypothesized as potential contributors to this steep increase, as these exposures have also changed during a similar window of time in particular regions. Pollen grains from anemophilous plants and fungal spores represent common groups of aeroallergens in ambient air.

In this project, we examined the geographical differences between four Canadian cities: Vancouver, Edmonton, Winnipeg, and Toronto, in 2008 through to 2012. The cities and years were chosen to cover the antenatal and perinatal windows of sensitization for the participants in the Canadian Healthy Infant Longitudinal Development (CHILD) birth cohort.

Results with the 5 year average for aeroallergen concentrations and season lengths duration will be presented. Differences in concentration of aeroallergens were found and, in line with previous findings are considered to be representative of the differences in the phytogeographical and climatic maps of the country.

The geographical differences found shows variation that may impact the particular needs of atopy prone and sensitized individuals in each city, and the need to account for local contexts when designing public health initiatives related to aeroallergens, green spaces, and climate change.

### 5.9. Green Prescriptions and Microbiome-Inspired Green Infrastructure (MIGI): Integrative Strategies to Enhance Personal and Planetary Health

RobinsonJake1Department of Landscape, University of Sheffield, Sheffield S10 2TN, UK2Sheffield and Rotherham Wildlife Trust, Sheffield S2 2SF, UK

Spending time in natural environments and connecting with nature both physically and psychologically bring a number of health and wellbeing benefits. One potential pathway to explain some of these health benefits is the exchange of microorganisms and other biogenic compounds between humans and the environment. It is thought that spending time in biodiverse environments and interacting with environmental microbiota could boost and regulate the immune system and help to sustain a healthy human ecosystem.

‘Green prescriptions’, also known as prescribed nature-based health interventions, have the potential to generate important health benefits through systematic prescribing and monitoring of interactions with natural environments, which includes the environmental microbiome. Building on the Microbiome Rewilding hypothesis, another integrative approach recently proposed is microbiome-inspired green infrastructure (MIGI), that is, designed multifunctional green spaces with the potential to generate health-regulating microbial interactions in urban environments.

Environmental health issues, such as biodiversity loss and ecosystem resilience, may appear to run concurrently to public health issues such as noncommunicable diseases (NCDs), however, they are often deeply interconnected. This has been acknowledged by recent calls to seek integrative approaches that address challenges associated with environmental and human health. Importantly, the above prospective strategies respond to these calls and have potential co-benefits including social integration, environmental stewardship, and ecological resilience, whilst forming part of a holistic, symbiotic, and ecological approach to the way we live. My interdisciplinary PhD research initially aims to investigate the current status of green prescribing in the UK—this includes answering questions such as who is providing green prescriptions, what activities are being delivered, where and how they are being delivered, and what are the co-benefits and constraints? Alongside this, I am conducting a review of what is known about the environment-microbiome-health axis with an exploration of the potential for MIGI as an integrative strategy. To bring these two research areas together, I am working with several general practitioners (GPs) in Sheffield, UK to establish an ambitious green prescribing service. As part of this project I will conduct an experiment on human-environment microbial exchange using ‘pocket foraging gardens’ in the premises of GP practices. This will then feed into a broader evaluation of the feasibility of green prescribing, providing a platform for future investigations and policy development.

**Suggested reading:** Jake M. Robinson and Martin F. Breed. Perspective Green Prescriptions and Their Co-Benefits: Integrative Strategies for Public and Environmental Health. Challenges 2019, 10(1), 9.

### 5.10. Wild at Heart—A Green Prescribing Project to Reduce Social Isolation

KingJenny^1^,^2^1Sheffield and Rotherham Wildlife Trust, Sheffield S2 2SF, UK2University of Sheffield, Sheffield S10 2TN, UK

**Background:** There is currently a public health crisis in the UK, and the situation is similar in many other countries across the planet. This is partially attributed to the effects of increased social isolation, reduced physical exercise and dietary deficiency. This, combined with financial insecurity and poor access to quality housing and surrounding natural environments, contributes to many people dying younger and living with preventable heath conditions as they age.

Social isolation is a significant risk factor for mortality and has been compared to smoking 15 cigarettes per day. People can become socially isolated for a variety of reasons, such as ageing, complex family and workplace issues, disability, illness and poor access to community and natural resources, for example, quality green spaces designed with inclusivity in mind.

**Sheffield and Rotherham Wildlife Trust** (SRWT) is a charitable organization in the city of Sheffield (UK), working closely with the local communities and the University of Sheffield. As part of SRWT’s strategic plan, we work to inspire people to engage with wildlife, and experience the health and wellbeing benefits of connecting with nature. We are helping to address various factors that contribute to a healthy lifestyle throughout the life-course and work alongside various stakeholders to promote a ‘walk the talk’ ethos.

**Wild at Heart** is a SRWT-led and National Lottery-funded project, working with local communities. Using a green prescribing model, we run weekly nature-based activity groups to reduce loneliness, increase social networks, improve wellbeing, and build personal resilience. When we feel valued and part of a community, we can be more resilient to life’s many complex challenges. We are able to reflect the interests of our participants, who are often from very deprived backgrounds, and make the project relevant and useful to them. We use the Warwick–Edinburgh Mental Wellbeing Scale (WEMWBS) to monitor participants every six weeks during their involvement with the group. This helps to build an evidence base to support the cause. In 2018, we received continuation funding for another 3 years, and have been commissioned by Rotherham Clinical Commissioning Group to provide a weekly nature-based activity session for their social prescribing service.

### 5.11. Contextualizing Health Promotion within Watersheds to Advancing SDG Goals

OnabolaChristiana O.ParkesMargot W.School of Health Sciences, University of Northern British Columbia, Prince George, BC V2N 4Z9, Canada

The mainstays of sustainable development are intricately linked to the determinants of health. Underscoring both domains are the triple bottom sustainability factors of social, environmental, and economic conditions, which are also closely linked to the complexity of socio-ecological interactions within a watershed. A watershed, as a complex network of nested ecosystems, includes land, water, and marine interactions, with inter-dependencies, and feedback loops, and a range of implications for social-ecological underpinnings of health and the Sustainable Development Goals across scales. A focus on watersheds as units of analysis underscores the importance of the interface between conservation, health, and development. Informed by the potential to use watersheds as ecologically coherent scales to examine both health and the SDG goals, this paper seeks to (a) identify gaps in understanding of health and SDGs in watersheds, and (b) examine tools and processes that can help understand and respond to the interactions among the SDGs within watersheds, including potential conflicts, opportunities, and implications for health and wellbeing.

Our paper will present preliminary findings that explore why, specifically, the stakeholders of SDG-3 (good health and wellbeing), SDG-14 (life below water), and SDG-15 (life on land) could benefit from actively prioritizing health and its socio-ecological underpinnings, using watersheds as settings, and units of analysis, to promote health and sustainability. The preliminary findings will focus on the use of geospatial tools, within the context of the Nechako watershed in British Columbia, Canada, to explore how SDGs can be ‘mapped’ within watersheds, identifying potential conflicts and examining how health and wellbeing considerations may factor into these conflicts. The use of maps also highlights pathways of health and wellbeing within the nested systems and cross-scale dynamics of watersheds, and identifies potential strategies that can be employed to accelerate achievements on targets for SDGs 3, 14, and 15. Discussion will focus on the potential of contextualizing health promotion within watershed settings in order to advance specific SDG goals, foregrounding ways in which scalar contexts and nested nature of watersheds could help inform how we see relationships across SDGs and create synergies that could be harnessed as pathways to promoting health, equity, and wellbeing.

## 6. Microbiome Rewilding: Restoring Health of Human and Environmental Habitats (Session Summary and Abstracts)

This session focused on the importance of microbes as the foundations of all ecosystems (including both human and environmental microbial systems) and addressing the impact of micro-scale ecological disruptions that occur as a result of dysbiotic drift.

### 6.1. KEYNOTE: Soil and Plant Contact Improve Diversity of Human Skin and Gut Microbiota: Nature-Based Immunomodulatory Interventions

Aki Sinkkonen ^1^ as the main Principal Investigator of the ADELE team (Mira Grönroos, Nan Hui, Olli H. Laitinen, Raul Kalvo, Noora Nurminen, Sami Oikarinen, Anirudra Parajuli, Riikka Puhakka, Marja I. Roslund, Laura Soininen, Heli K. Vari, Guoyong Yan, Juho Rajaniemi, Heikki Hyöty, Aki Sinkkonen) ^1,2^

The ADELE team studies effects of everyday living environments on health, including microbial communities and plant growth, soil remediation, and ecology, utilizing cross-disciplinary metadata to reveal novel associations

^1^  Ecosystems and Environment Research Program, University of Helsinki, 15140 Lahti, Finland
^2^  Faculty of Medicine and Health Technology, and Faculty of Built Environment Tampere University, 33100 Tampere, Finland

Progressive lifestyle changes and “cleaner environments” have been associated with significant increases in inflammation and chronic diseases. This is exemplified by comparisons of genetically similar populations of Finland and Karelia which experienced different rates of environmental change. Finland, which has shown more rapid environmental changes towards built environments, has higher rates of immune diseases (allergy, thyroid disease, celiac disease, and type 1 diabetes) than Karelia. Declining contact with microbes and microbial products has been implicated, with extensive studies in animal models demonstrating the adverse effects on immune development and disease risk.

Our studies demonstrate the built environment reduces transfer of microbes indoors. Bacterial diversity is lower in urban areas, and this can be measured by reduces doormat diversity in built areas.

We have also shown that “greening” interventions can improve diversity of both the environment and the humans active in those environments. For example, yard greening at daycare centers increases the soil microbial diversity, and it has a potential to change microbiota of children assessed after 30 days. We have also seen preliminary evidence of immunomodulation. Daycare personnel reported with differences in behavior, including increases in physical activity, unstructured play (playing without toys), and positive impact on mood and inspiration. This shows the benefits of contact with nature, soil, and gardening materials on health.

Extending this, we have explored the role of developing specific materials with these qualities, derived from soils and plants to create ‘biodiversity powders’. These are not intended to substitute for natural contact, but for benefit to people who have restricted access to nature. We have developed several materials that show similar diversity to samples collected from the forest floor (and more diverse than commercial sand in children’s sand boxes). Short-term experiments show that these novel materials increase skin microbial diversity directly, or when added to sand. We then performed an extended 2-week exposure trial which showed that “exposed” adult volunteers showed increased stool and skin alpha diversity compared to controls 3 weeks after the end of the exposure period, suggesting a sustained effect. Significantly, the stool and skin diversity correlated to TGF-β levels in the exposure group (*p* = 0.001).

We are now examining this in children to determine the effects of adding these microbial communities to soil in sandboxes on child immune function and health. Preliminary findings are in line with the results of exposure trials made with adults, but this work is ongoing. Our goal is to find practical, noninvasive solutions to safely increase environmental microbial exposure for optimal immune function and long term health.

**Suggested Reading:** Grönroos M, Parajuli A, Laitinen OH, Roslund M, Vari H, Hyöty H, Puhakka R, Sinkkonen A. 2019. Short-term direct contact with soil and plant materials leads to an immediate increase in diversity of skin microbiota. MicrobiologyOpen 8: e645. DOI: 10.1002/mbo3.645

Hui N, Mira Grönroos, Marja I. Roslund, Anirudra Parajuli, Heli K. Vari, Laura Soininen, Olli H. Laitinen, Aki Sinkkonen and The ADELE Research Group. Diverse Environmental Microbiota as a Tool to Augment Biodiversity in Urban Landscaping Materials. Frontiers in Microbiology 2019; 10: 536. DOI: 10.3389/fmicb.2019.00536

Hui N, Parajuli A, Puhakka R, Grönroos M, Roslund M, Vari HK, Selonen VAO, Siter N, Nurminen N, Oikarinen S, Yan G, Laitinen OH, Rajaniemi J, Hyöty H, Sinkkonen A. 2019. Temporal variation in indoor transfer of dirt-associated environmental bacteria in agricultural and urban areas. Environment International 132 (11): 105069. DOI: https://doi.org/10.1016/j.envint.2019.105069

Nurminen, N., J. Lin, M. Gronroos, R. Puhakka, L. Kramna, H. K. Vari, H. Viskari, S. Oikarinen, M. Roslund, A. Parajuli, I. Tyni, O. Cinek, O. Laitinen, H. Hyoty and A. Sinkkonen (2018). "Nature-derived microbiota exposure as a novel immunomodulatory approach." Future Microbiol 13: 737–744. DOI: 10.2217/fmb-2017-0286

### 6.2. Urban Habitat Restoration: Rewilding of Urban Microbiota through Native Revegetation of Urban Green Spaces

MillsJacob G.^1^,^2^GellieNicholas J.C.^1^BissettAndrew^3^LoweAndrew J.^1^BreedMartin F.^1^,^2^1Environment Institute and School of Biological Sciences, The University of Adelaide, Adelaide, SA 5005, Australia2Healthy Urban Microbiome Initiative (HUMI), Adelaide, SA 5005, Australia3CSIRO (Commonwealth Scientific and Industrial Research Organisation), Adelaide, SA 5000, Australia

Humans, like all multi-cellular life, evolved in a microbial world where coevolution with symbiotic microbiota was, and still is, unavoidable. This coevolution has created a symphony of biological processes involved in human development, such as signaling for the development of mature organs and immune functions. Therefore, the inhibited colonization of humans by microbiota from an unnatural human habitat, such as an industrialized city, leads to negative health outcomes. Epidemic rates of non-communicable diseases (e.g., chronic inflammatory and autoimmune diseases) continue to rise in industrialized urban societies. It is hypothesized that this trend will continue until lifestyle factors that improve the state of the symbiotic microbiota are adopted. These factors include diversifying diets, reducing antibiotic use, and increasing exposure to biodiversity. Plants, the primary producers in biodiverse environments, form their own complex microbial symbioses. Previous work has shown that restoring plant communities in wild settings changes the environmental microbiome. This occurs through genetic and environmental factors selecting for symbionts, and the attraction of other animals which bring their own microbiota. Therefore, restoration can be used to shape the environmental microbiome, a key source of symbiotic human microbiota. We have previously proposed that Microbiome Rewilding, the restoration of urban biodiversity to improve the interaction between urban residents and environmental microbial diversity, can be a public health intervention by restoring the microbial exposure of urban residents to a more natural state. Here, I will discuss the evidence for biodiversity acting as a mediator of public health through microbial ecology, some knowledge gaps of Microbiome Rewilding, and some of our own findings showing the relationships between urban vegetation diversity and soil microbiome structure.

### 6.3. Urban Pollution Alters Environmental and Kindergarten Children’s Commensal Microbiota—Implications for Health

RoslundMarja^1^OikarinenS.^2^PuhakkaR.^1^NurminenN.^2^RantalaS.^1^HuiN.^1^RantalainenA.-L.^1^LaitinenO.H.^2^HyötyH.^2^SinkkonenAki^1^1Faculty of Biological and Environmental Sciences, Ecosystems and Environment Research Program, University of Helsinki, 15140 Lahti, Finland2Faculty of Medicine and Health Technology, Tampere University, 33100 Tampere, Finland

Today, more than half of the human population lives in urban areas with limited contact to natural biodiversity. Over 80% of urbanites are exposed to air quality levels exceeding the World Health Organization limits. Biodiversity loss and urban pollution both affect the microbial communities in urban environment. Altered environmental microbiota in living environment is associated with increased prevalence of immune-mediated diseases.

Polycyclic aromatic hydrocarbons (PAHs) have been classified as priority environmental pollutants that accumulate in urban soils. Our previous results indicate that PAH contamination may change the abundance and diversity of bacteria that are associated with human health and immune system. In addition, PAHs are known to cause direct health deficits, such as cancer. For these reasons, we investigated the associations between PAH pollution, and environmental and human commensal microbiota in kindergartens. Additionally, we designed biodiverse landscaping materials for kindergartens yards to mitigate the effect of PAH pollution, and to induce beneficial effects on human immune system.

We measured PAHs from soil and air of eleven urban kindergartens in Finland. We analyzed kindergartens’ soil and children’s commensal (gut and skin) bacteria communities with 16S rRNA gene metabarcoding and used KEGG (Kyoto Encyclopaedia of Genes and Genomes) database to categorize functional pathways. We used passive SPMD (SemiPermeable Membrane Device) sampler to measure the concentrations of PAHs in the ambient air. SPMD sampler absorbs gaseous PAHs and simulates the process of bioconcentration in the fatty tissue. To assess the PAH hazard to children’s health, we used the toxicity benzo(a)pyrene total potency equivalent (BaPtpe) and compared the obtained BaPtpe levels to the incremental lifetime cancer risk for BaP.

Concentrations of fluoranthene in soil exceeded environmental threshold value in two kindergartens. In one soil sample BaPtpe was over two times higher than the allowed threshold for incremental lifetime cancer risk. Total PAHs were above background levels in several yards but below environmental and safety threshold values. PAH concentrations were associated with several bacterial taxa, including taxa associated with human health and immune mediated diseases. Predictive functional analysis revealed associations between KEGG functional pathways and PAHs levels, particularly chrysene level in the air and PPAR (Peroxisome proliferator-activated receptors) pathway in endocrine system. The results and nature-based solutions will be discussed from the viewpoint of environmental and child health.

**Suggested reading**: Roslund, M. I. et al. Half-lives of PAHs and temporal microbiota changes in commonly used urban landscaping materials. PeerJ 6, e4508 (2018).

Parajuli, A. et al. The abundance of health-associated bacteria is altered in PAH polluted soils—Implications for health in urban areas? PLoS One 12, 1–18 (2017).

Parajuli, A. et al. Urbanization Reduces Transfer of Diverse Environmental Microbiota Indoors. Front. Microbiol. 9, 1–13 (2018).

Hanski, I. et al. Environmental biodiversity, human microbiota, and allergy are interrelated. Proc. Natl. Acad. Sci. 109, 8334–8339 (2012).

Kondrashova, A., Seiskari, T., Ilonen, J., Knip, M. & Hyöty, H. The ‘Hygiene hypothesis’ and the sharp gradient in the incidence of autoimmune and allergic diseases between Russian Karelia and Finland. Apmis 121, 478–493 (2013).

Stein, M. M. et al. Innate Immunity and Asthma Risk in Amish and Hutterite Farm Children. N. Engl. J. Med. 375, 411–421 (2016).

### 6.4. Microbes Modulate the Aging Process: Immune Resilience, Accelerated Healing, and Curtailed Inflammation

Susan Erdman (Head, Erdman Lab, Division of Comparative Medicine, Massachusetts Institute of Technology, Cambridge, MA 02139, USA)

Dimensions of “good health” span from physical, mental, social, and spiritual health and are all interdependent. We are increasingly understanding the biological pathways that connect these elements to promote resilience, including our sense of spiritual wellbeing. Surprisingly, microbes are emerging at the center of these relationships with implications for the aging process.

Healthful longevity is increasingly recognized as a function of optimally regulated immune function, which is highly dependent on the microbiota in and around our bodies. In particular the gut microbiome, which plays a critical role in promoting the immune regulatory pathways that prevent systemic inflammation and chronic disease. Optimizing microbial exposure, particularly in early life, therefore, has major multi-generational public health implications.

In health, the microbiome plays a key role in the healthy function of virtually all systems. Based on a multitude of data from animal models, gut microbes appear to suppress inflammation both locally and systemically, attenuating the oxidative stress associated with the aging process. Moreover, there is increased tissue repair, which has clear implication for aging processes. The transfer of immune cells recreates beneficial effects in untreated animals, confirming immune-mediated pathways. These microbial immunomodulatory events also influence the brain with effects on mood and behavior in many studies.

We have shown that adding specific probiotics such as *Lactobacillus reuteri* from human milk to mouse drinking water can have “anti-aging” effects. This includes ameliorating the adverse effects of a “fast food” diet on aging process in mice. In addition to the multitude of anti-inflammatory effects and increased survival, we consistently observed accelerated wound healing and more luxuriant new hair growth and a “glow of health” in the probiotic treated group. We also see transgenerational effects. Wound healing is improved, and obesity is prevented in subsequent generations (on a western diet) when their “grandma” was supplemented with *Lactobacillus reuteri*.

Interestingly, many of these effects appear to be mediated through a shift in stress responses—we saw increased oxytocin levels (the “love hormone”), and reduced plasma corticosterone levels with effects on psychological wellbeing and social behavior. Collectively, these studies provide new perspectives on the role of microbes in mediating many of the protective pathways that ameliorate the aging process not only the physical benefits (anti-inflammatory effects and tissue repair), but also the psychosocial effects that are important in the aging process.

**Suggested reading:** Erdman SE, Poutahidis T. Probiotic ‘glow of health’: it’s more than skin deep. *Benef. Microbes* 1 June 2014; 5(2): 109–119.

### 6.5. Immunization with a Soil-Derived Bacterium, Mycobacterium vaccae, Prevents Stress-Induced Anxiety, Neuroinflammation, and Microglial Priming

LowryChristopher A.^1^,^2^,^3^,^4^,^5^1Department of Integrative Physiology, Center for Neuroscience, and Center for Microbial Exploration, University of Colorado Boulder, Boulder, CO 80309, USA2Departments of Psychiatry, Neurology, and Physical Medicine and Rehabilitation, University of Colorado Anschutz Medical Campus, Aurora, CO 80045, USA3Rocky Mountain Mental Illness Research Education and Clinical Center (MIRECC), Rocky Mountain Regional Veterans Affairs Medical Center (RMRVAMC), Aurora, CO 80045, USA4Military and Veteran Microbiome: Consortium for Research and Education (MVM-CoRE), Denver, CO 80220, USA5Senior Fellow, inVIVO Planetary Health, of the Worldwide Universities Network (WUN), West New York, NJ 07093, USA

Novel prevention and treatment strategies are needed to reduce the burden of anxiety disorders, trauma and stressor-related psychiatric disorders such as posttraumatic stress disorder (PTSD), and affective disorders. Both preclinical and clinical studies suggest that inflammation increases vulnerability to development of psychiatric disorders. Consequently, immunoregulatory strategies to decrease inflammation have potential for the prevention and treatment of these disorders. Using a murine model of chronic psychosocial stress, the chronic subordinate colony housing (CSC) model, we found that psychosocial stress induces gut dysbiosis, characterized by proliferation of pathobionts and host inflammation. Immunization with a heat-killed preparation of the soil-derived bacterium, *Mycobacterium vaccae* NCTC 11659, a bioimmunomodulatory agent previously shown to activate regulatory T cells (Treg) and to increase production of anti-inflammatory cytokines including interleukin 10 (IL-10) and transforming growth factor beta (TGFβ), prevented stress-induced exaggeration of anxiety and development of a PTSD-like syndrome. Analysis suggests that the protective effects of *M. vaccae* immunization are due to protection from a stress-induced proinflammatory gut microbial community. In other studies, we have shown that immunization with *M. vaccae* induces an anti-inflammatory immunophenotype in the brain, characterized by increased expression of the anti-inflammatory cytokine, interleukin 4 (IL-4), prevents stress-induced anxiety-like defensive behavioral responses, and prevents stress-induced hippocampal microglial priming. Together, these data support the use of bacteria with anti-inflammatory and immunoregulatory properties for prevention and treatment of anxiety disorders and trauma- and stressor-related disorders such as PTSD.

**Suggested reading:** Langgartner D, Peterlik D, Foertsch S et al. Individual differences in stress vulnerability: The role of gut pathobionts in stress-induced colitis. Brain Behav Immun 2017;64:23–32.

Reber SO, Siebler PH, Donner NC et al. Immunization with a heat-killed preparation of the environmental bacterium *Mycobacterium vaccae* promotes stress resilience in mice. Proc Natl Acad Sci U S A 2016;113:E3130–E3139.

Reber SO, Langgartner D, Foertsch S et al. Chronic subordinate colony housing paradigm: A mouse model for mechanisms of PTSD vulnerability, targeted prevention, and treatment-2016 Curt Richter Award Paper. Psychoneuroendocrinology 2016;74:221–230.

Eraly SA, Nievergelt CM, Maihofer AX et al. Assessment of plasma C-reactive protein as a biomarker of posttraumatic stress disorder risk. JAMA Psychiatry 2014;71:423–431.

Lowry CA, Smith DG, Siebler PH et al. The microbiota, immunoregulation, and mental health: Implications for public health. Curr Environ Health Rep 2016;3:270–286.

Zuany-Amorim C, Sawicka E, Manlius C et al. Suppression of airway eosinophilia by killed *Mycobacterium vaccae*-induced allergen-specific regulatory T-cells. Nat Med 2002;8:625–629.

Frank MG, Fonken LK, Dolzani SD et al. Immunization with *Mycobacterium vaccae* induces an anti-inflammatory milieu in the CNS: Attenuation of stress-induced microglial priming, alarmins and anxiety-like behavior. Brain Behav Immun 2018;73:352–363.

### 6.6. Diet and Microbiome Rewilding: Lessons from Lifestyle Changes in Migrating Populations

Abby Johnson (Postdoctoral associate, computational nutritionist and registered dietitian with the Knights Lab studying the personalized interactions between diet and the microbiome; University of Minnesota, Biotechnology Institute Falcon Heights, MN 55108, USA)

Epidemiological studies have shown that US immigration leads to weight gain and while the causes are likely multifactorial, environmental changes have been implicated in changes in human-associated microbiomes in ways that adversely affect health and metabolism. Understanding the causes and consequences of modern dysbiosis is key to understanding the health paradox of worsened health outcomes after migration to relatively more “privileged” westernized environments.

We have been working in partnership with the Hmong and Karen refugee and immigrant communities in Minnesota to explore the effects of migration on the microbiome, and found that years spent in US explains a large amount of the variation in gut microbiome composition. Gut biodiversity decreases with both US residence and with obesity. Immigrants lose key microbes and acquire new bacteria prevalent in the American population and this is compounded across generations. Specifically, *Bacteroides* strains displace *Prevotella* strains across generations in the United States. In addition to changes in composition, immigration was associated with bacterial communities without the genes needed to build specific enzymes for plant fiber degradation.

This raises the key question: how much do dietary changes contribute to alterations in the microbiome with migration?

To address this we modeled differences in dietary intake using a hierarchical dietary “tree” that accounts for food groups and food relatedness in statistical analysis. This model allows for analysis of new foods and detailed analysis of how microbiome composition pairs with food intake, providing a more sophisticated dietary profile than possible from traditional nutrient data from dietary records. Using this tree-based analysis we observed clear patterns of dietary acculturation associated with US residence. However, dietary variation only partly explained microbiome variation across individuals.

Our findings raise important questions around how we restore diversity in western environments and protect diversity within migrating populations. This requires a better understanding of the impact of western processed diets on the microbiome and the other environmental factors contributing to biodiversity loss at the macro and micro scales.

**Suggested reading:** P. Vangay, A. J. Johnson, T. L. Ward, K. A. Culhane-Pera and D. Knights (2018). US Immigration Westernizes the Human Gut Microbiome. Cell 175(4): 962–972 e910.

A.J. Johnson, P. Vangay G.A. Al Ghalith..., R. Menon, K. Koecher and D. Knights (2019). Daily Sampling Reveals Personalized Diet-Microbiome Associations in Humans. Cell Host & Microbe 25(6):789–802.e5.

## 7. Food Justice and Nutritional Ecology: Influencing Food Choices, Values and Systems

This session examined the role of food systems in health at all scales, including the adverse effects of unhealthy food systems (including ultra-processed foods on individuals, societies and the environment) and the importance of ‘planetary diets’ which consider the optimal food systems for health on *all* of these scales.

### 7.1. KEYNOTE: Food for Our Future: Taking Responsibility at the Cross-Roads of Personal and Planetary Health

David L. Katz, Founder/President of the True Health Initiative; Founding Director of Yale University’s Yale-Griffin Prevention Research Center, Griffin Hospital, Derby, CT 06418, USA, Past-President of the American College of Lifestyle Medicine

Humans, like all (wild) animal species, once knew how to eat on the basis of native adaptations, experience, empiricism, and the constraints of a natural food supply. Whereas science should only have served to populate gaps in established knowledge, it has instead been occasionally corrupted in its conduct, and far more often distorted in its communication. The result is that with ever more information about diet and health, there is also ever more misinformation, and resultant discord, debate, and confusion, and perhaps a modicum of collusion as well. Despite this semblance of doubt, the fundamental truths of diet for the health of people and planet alike—predicated on a massive aggregation of remarkably consistent science, an application of sense, and the global consensus of experts—is hiding in plain sight, like that proverbial elephant in the room. Diets of minimally processed foods, predominantly plants (i.e., vegetables, fruits, legumes/beans, nuts, seeds, whole grains), and plain water preferentially for thirst are consistently associated with more years in life, more life in years. Fortuitously, the same dietary priorities redound to the benefit of the climate, aquifers, and biodiversity as well. Many industries profit from the propagation of pseudo-confusion about food, from Big Food which engineers willfully addictive junk foods, to Big Pharma which sells products to treat diseases people never needed to get. This status quo has persisted for decades, squandering to date an opportunity to eliminate some 80% of premature death and chronic disease in the modern world, and safeguard the imperiled resources of our natural world Humans are constitutional omnivores, with dietary choices. The information on which to base salutary choices is abundantly available. Such knowledge, however, is only true power when applied. Finding new ways to elevate the signal of such truth above the constant, noisy discard, and to convert what we have long known about food and health into what we do at last is a signature challenge of our time. There are ample ways to meet it if we have the collective will.

### 7.2. KEYNOTE: The EAT-Lancet Commission on Food, Planet, Health

Macro Springmann (Oxford Martin Programme on the Future of Food, Nuffield Department of Population Health, University of Oxford, Oxford OX3 7LF, UK)

The current food system is environmentally unsustainable. It is a major driver of climate change, land-use change and biodiversity loss, freshwater use, and a major polluter of terrestrial and aquatic systems through fertilizer runoff. Current diets are also not healthy either. Global prevalence of overweight increased over a third, and obesity rates doubled over last 30 years. Dietary risks are leading risk factors for chronic disease globally and in most regions.

Against this backdrop, the EAT-Lancet Commission on Healthy Diets from Sustainable Food Systems was formed to describe a sustainable food system that can deliver healthy diets for a growing population. The Commission included 19 commissioners and 18 co-authors from 16 countries and various fields, including human health, agriculture, political science, and environmental sustainability. Our aims were to (a) define a healthy reference diet, (b) define the planetary boundaries of the food system, (c) analyze diets and food system changes to stay within planetary boundaries, and (d) outline strategies to achieve healthy diets from sustainable food systems by 2050.

We conducted a comprehensive review of the literature on healthy eating to define a healthy reference diet that can be adapted to local context. The reference diet we developed, based in large part on epidemiological evidence, is predominantly plant-based. It contains generous amounts of vegetables, fruits, legumes, nuts and seeds, and whole grains, moderate amounts of fish, poultry, and dairy, and low amounts of red meat, such as beef and pork. Several dietary patterns that have been and are consumed by several population groups are compatible with the reference diet, including predominantly plant-based varieties of flexitarian diets, such as Mediterranean, pescatarian diets, vegetarian diets, and vegan diets.

For the Commission, we analyzed these dietary patterns with respect to their nutrient content, impacts on chronic-disease mortality, and extend of environmental resource use and pollution. Our analyses showed that most of the current potential nutrient shortfalls could be addressed with dietary changes towards the healthy reference diets, that premature mortality from chronic diseases could be reduced by about 20%, and that environmental resource use and greenhouse gas emissions can be reduced sufficiently to allow the food system to stay within planetary boundaries when combined other food-system changes. These changes included improvements in technology and management at the farm level, reductions in food loss and waste, and positive socio-economic developments. Whilst all measures contributed to the reductions in resource use that were needed to stay within environmental limits, it was particularly the dietary changes that were necessary to reduce the greenhouse gas emissions of the food system in line with limiting global warming to below two degrees Celsius.

A great transformation of the food system will be needed to build a sustainable food system that can feed a growing population healthily. The policy measures that can contribute to this transformation include farm-level incentives and support to change what foods are grown, and how they are grown; better environmental regulation, for example of water use and quality, biodiversity, and climate change, to create an environmentally sustainable operating space for farmers and businesses; investments in public infrastructure, in particular in low and middle-income countries, to reduce food loss and waste and improve agricultural yields; and multilevel incentives for consumers to adopt healthier and more sustainable diets, including informational, fiscal, and other regulatory measures. Good starting points would be to include sustainability considerations in national dietary guidelines, and to align agricultural incentives with health and environmental goals. Action will be needed along the whole food chain, from farmers over businesses to policymakers and citizens.

**Suggested reading (including country-level data):** Willett et al, 2019, Food in the Anthropocene: the EAT-Lancet Commission on healthy diets from sustainable food systems, The Lancet 392:10270, 447–492.

Springmann et al, 2018, Options for keeping the food system within environmental limits, Nature 562, 519–525.

Springmann et al, 2018, Health and nutritional aspects of sustainable diet strategies and their association with environmental impacts: a global modelling analysis with country-level detail, Lancet Planetary Health 2, e451–e461

### 7.3. The Impact of Ultra-Processed Foods on Person, Place and Planet—and What We Do about it

Jean-Claude Moubarac (Department of Nutrition, University of Montréal Medical School, Montreal, QC H3T 1J4, Canada.)

The twentieth century saw a massive shift in food systems towards ultraprocessed food (UPF) and drink (UPD) formulations and this has continued with an associated shift in food consumption towards more snacking on ready to consume food and drink products. Since 2000, the increase in UPF volume sales was highest for South and Southeast Asia (67.3%) and North Africa and the Middle East (57.6%), while the increase in UPD (mostly carbonated drinks) was highest for South and Southeast Asia (120.0%) and Africa (70.7%). We examined the associations between the volume of sales/capita and adult BMI trajectories, controlling for confounding factors and observed that the increased volume of sales/capita were positively associated with population-level BMI trajectories.

Collectively, population-based, cross-sectional and cohort studies provide good evidence that UFP are detrimental to health. A new in-patient randomized controlled trial also shows that diets high in ultra-processed foods cause rapid increases in dietary energy intake and body weight and fat.

There is also evidence that these foods may be associated adverse changes in the microbiome, with significant implications for inflammation and metabolism. For instance, both advanced glycation end products created by high-temperature extrusion and cosmetic additives such as emulsifiers and artificial sweeteners cause changes in the composition and metabolic behavior of the gut microbiota that lead to diverse forms of inflammatory-related disease.

Thus, while some food processing is necessary and important, ultra-processed foods are often high energy and low nutrient foods with very little if any whole food. They contain high amounts of sugar, salt, oils, and fats, and are often derived from industrial process not traditionally used in domestic settings as high-temperature extrusion and molding. The often include of flavors, colors, emulsifiers, and other cosmetic additives designed to mask their basic ingredients and to make the final product attractive and more palatable. Many are seldom if ever used in culinary preparation, contributing to declining knowledge and practice of traditional domestic food preparation. While these foods are produced at relatively low cost to industry, they can have high personal, social, and environmental costs.

The NOVA food classification was created to increase awareness and identification of these foods in attempt to influence both policy and practice towards more fresh and unprocessed foods and culinary practices that are both healthy and more sustainable.

- NOVA group 1: raw foods, unprocessed or minimally processed foods.
- NOVA group 2: processed culinary ingredients from group 1 foods.
- NOVA group 3: Products made by adding salt, oil, sugar, or other group 2 ingredients to group 1 foods, using preservation methods such as canning and bottling, and, in the case of breads and cheeses, using non-alcoholic fermentation.
- NOVA group 4: Formulations of ingredients, mostly of exclusive industrial use, that result from a series of industrial processes (hence ‘ultra-processed’), many requiring sophisticated equipment and technology.

It is our hope that this delineation and the growing evidence of adverse health impacts of UFP and UFD should generate policies and actions that further protect public health as well as culture, livelihoods, rational food systems, and the living and physical world for the health of people, place, and planet.

**Suggested reading:** Monteiro CA, Cannon G, Moubarac JC, Levy RB, Louzada MLC, Jaime PC. Freshly Prepared Meals and Not Ultra-Processed Foods. Cell Metab. 2019 Jul 2;30(1):5–6

Vandevijvere S, Jaacks LM, Monteiro CA, Moubarac JC, Girling-Butcher M, Lee AC, Pan A, Bentham J, Swinburn B. Global trends in ultraprocessed food and drink product sales and their association with adult body mass index trajectories. Obes Rev. 2019 May 17. (ePub ahead of press)

Moubarac JC, Parra DC, Cannon G, Monteiro CA. Food Classification Systems Based on Food Processing: Significance and Implications for Policies and Actions: A Systematic Literature Review and Assessment. Curr Obes Rep. 2014 Jun;3(2):256–272.

Monteiro C.A., Cannon G., Moubarac J.-C., Levy R.B., Louzada M.L.C., Jaime P.C. The UN Decade of Nutrition, the NOVA food classification and the trouble with ultra-processing Public Health Nutr., 21 (2018), pp. 5–17

### 7.4. Poverty, Inequity and Caloric Burden—How Both Social Reality and Perception Drive Unhealthy Food Consumption

Michelle Cardel (Assistant Professor, Department of Health Outcomes and Biomedical Informatics; Director, Obesity Research Alliance; Associate Director, Center for Integrative Cardiovascular and Metabolic Diseases, University of Florida College of Medicine, Gainesville, FL 32611, USA)

There has been a staggering increase in obesity in much of the developed and developing world. While this is multifactorial, many of the risk factors (physical, environmental, behavioral, psychological, and social) associate with low socioeconomic status (SES).

There are two consistent themes in the literature. The first focuses on individual characteristics of persons with low SES, and attributes individual characteristics to development of obesity including the levels of education, stress, discrimination, and resources available. The second theme focuses on the obesogenic built environment in low SES neighborhoods—environments that promote the consumption of energy dense foods and dissuade physical activity—such as fast food access to recreational facilities, walkability, healthful foods, and safety.

But do either of these models fully explain the SES effect? New interventions in low SES women challenge this notion and reveal that providing education and resources is not enough to make a positive impact on weight, and in some individuals may even lead to weight gain.

In fact, there is emerging evidence that simply feeling of low status may alter behavior in ways that increase obesity. In a multitude of animal models, lower social status (subordinate status) is related to a variety of physiological and behavioral changes including effects on cortisol (various primates), blood pressure (rats, rabbits, baboons, macaques), heart rate (various primates) and accumulation of visceral fat (rats). This also influences energy-dense food consumption (socially-housed monkeys, rats), cardiovascular disease (mice), and shortened lifespan (mice).

In other words, perceptions of being “lower on the totem pole” could be driving individuals to consume excessive energy intake. Perceiving yourself to be in a lower social status position leaves you in a position to feel your environment is insecure, leading you to consume more calories—as corroborated by the large body of animal literature—physiologically and behaviorally driving positive energy balance and obesity.

A social experiment that illustrates this is the difference in food consumptions in fans depending on whether their teams win or lose. Saturated fat and energy intake are documented to increase in cities with losing teams and decreased in cities with winning teams compared to no change in levels in cities without a team or one that did not play. This shows the acute behavioral effects of even transient experiences of losing—being placed in a lower social status position is enough to drive excess calorie consumption.

Based on this we have proposed a third theme that may be contributing to the SES influence on obesity: being in a low social status position may influence your eating behavior and risk for obesity. This is adding the important additional dimension of self-perception of social status in addition to objective external measures. This assumes additional factors beyond access to material resources must be playing a role in the relationship between social status and obesity and influencing physiological drive to consume excess calories when placed in a lower social status position. While observational studies support this, experimental models are required to help identify causal mechanisms underlying low social status as a pathway for obesity.

To address this, we initially undertook a feasibility study using a manipulated game of Monopoly. Our aim was to investigate the effect of experimentally manipulated social status on ad libitum acute energy intakes and eating behavior. We used a randomized crossover design to place participants in experimental high and low social status conditions. Our hypothesis was that under the low social status condition, individuals would consume a greater number of calories, fat, sodium, and sugar when compared to the high social status condition. We studied 19–25 year old Hispanics (n = 9; mean age 22.3, 67% female, Body Mass Index ≥18.5 kg/m^2^ and ≤30 kg/m^2^). When participants returned for their second study visit, the protocol was identical, but they were placed in the opposite social status condition. “Losing” was associated with reduced measures of pride and powerfulness, and with increased consumption of calories, % of calorie needs, and saturated fat. This was the first study experimental manipulation to demonstrate that low social status position may influence your eating behavior and risk for obesity. These findings have been corroborated by recent publications demonstrating that when individuals are randomized and primed to a “rich” or “poor” condition, they ate significantly more calories. The mere experience of being put in a low social status condition resulted in an increase in ghrelin and caloric intake.

Currently confirming findings in a randomized controlled trial, the publication is in process (n = 133, 15–21 year old Hispanics). This randomized controlled trial builds on the previous feasibility study to be significantly powered to detect differences in energy balance. Preliminary findings suggest that women and individuals with food insecurity consume excess calories in low social status conditions. These results appear to be independent of stress, suggesting that the mere experience of being put in a low social status condition is enough to induce excess caloric intake among women and food-insecure individuals. Future directions for this research include how low social status, scarcity, food insecurity, deprivation, and social inequality may serve as an obesogenic environment.

As we move forward with personalized medicine, it is important to consider that psychosocial components play a role in the development of obesity—your ZIP Code may be just as important as your genetic code.

**Suggested reading:** Cardel MI, Johnson SL, Beck J, Keita AD, Tomczik AC, Pavela G, Huo T, Janicke DM, Muller K, Piff PK, Peters JC, Hill JO, Allison DB. The effects of experimentally manipulated social status on acute eating behavior: A randomized, crossover pilot study. Physiology & Behavior. 2016. 162:93–101.

### 7.5. Corporate Responsibility: Can B Corp “B” the Change?

Johan Garssen (Director Center of Excellence at Danone-Nutricia Research; Head of the Pharmacology division, Full Professor Immunopharmacology, Utrecht University, 3512 JE Utrecht, Netherlands

Society’s most challenging problems cannot be solved by government and nonprofits alone. B Corps form a community of leaders and drive a global movement of people using business as a force for good. B Corps are accelerating a global culture shift to redefine success in business and build a more inclusive and sustainable economy. These businesses meet the highest standards of verified social and environmental performance, public transparency, and legal accountability to balance profit and purpose. The key elements in becoming B corp include:

- **Transparency**—B Corps must publish their assessment and any major legal disclosures on the B Corp website.
- **Continuous Improvement**—The B Corp philosophy is one of continuous improvement.
- **Universal commitment**—Being a B Corp is a entire corporation effort and achievement, all teams and employees should be involved as part of the movement.
- **Recertification**—B Corps must recertify every 2 to 3 years.

Together the B Corp community works to change attitudes and behaviors toward reduced inequality, lower levels of poverty, a healthier environment, stronger communities, and the creation of more high quality jobs with dignity and purpose. By harnessing the power of business, B Corps use profits and growth as a means to a greater end: positive impact for their employees, communities, and the environment.

## 8. Developmental Origins: Building Healthier Foundations (Session Summary and Abstracts)

The health of our future depends on the health of the environment from the first moments of life. This session underscored the importance of the ecology of the early environment for life-long health, including the promotion of healthy behaviors and value systems in childhood.

### 8.1. Newborn Gut Microbiome Predicts Later Allergy and Asthma

JohnsonChristine C.^1^WegienkaGanesa^1^SitarikAlex^1^JonesKyra^1^OwnbyDennis^1^ZorattiEdward^1^FujimuraKei^2^LynchSusan^2^1Department of Public Health Sciences, Henry Ford Health System; Center for Urban Responses to Environmental Stressors (CURES), Wayne State University Detroit, MI 48202, USA, Detroit, MI 48202, USA2Divison of Gastroenterology, Department of Medicine, University of California San Francisco (UCSF), San Francisco, CA 94143, USA

Microbes are the most abundant form of life on our planet and are critical to the health of all ecosystems including our own. They comprise more than half the cells in the human body and contribute to over 99% of ‘our’ genetic material. Disruption of microbial systems are implicated in changes in physiology, metabolism, and immune function, and can lead to increases in human disease. These effects are greatest in early life when these systems and responses are programmed. The rising rates of pediatric allergic disease is a prime example.

We hypothesize that changes in the infant gut microbiome are implicated in the epidemic rise in allergic disorders. This is based on the rationale that the rapid rise in allergic conditions points to environmental rather than genetic causes, and that environmental factors may alter early life intestinal microbiota composition, resulting in improper immune development. We propose that antenatal (maternal behaviors, SES, pregnancy microbiota), perinatal (mode of delivery, breast milk and diet) and early postnatal (infections, antibiotics, exposure to pets, other children, pollutants, allergens, stress) factors interact with the host microbiome and host genotypes to alter immune development and disease risk.

To explore this conceptual model, we examined the associations between early life gut microbiota composition and allergic conditions in a longitudinal birth cohort—the Wayne County Health, Environment, Allergy and Asthma Longitudinal Study (WHEALS). This is a socio-economically and racially diverse (60% minority) population-based birth cohort (enrolled between 2003 and 2007). We analyzed 298 infant stool samples collected between 0 and 11 months, targeting neonates (median age 1.2 months; *n* = 130) and infants (median age 6.6 months; *n* = 168). The gut microbiota composition was examined using 16S V4 and ITS2 for both bacterial and fungal taxa.

First, we demonstrated gut microbiota change with age with overall shifts from Enterobacteriaceae to Lachnospiraceae (increases in 13 families of anaerobes and decreases in 8 families of facultative anaerobes).

Next, we examined whether early life gut microbiota influence allergic asthma at age 10–12 years. We observed that gut bacterial diversification started as more diversified but subsequently development was slower in the first year of life in children who had allergic asthma at 10 years of age. There was a strong interaction by gender making this trajectory difference only evident in females. Both males and females whose gut bacterial communities were more mature at 1 month of age and immature at 6 months of age had increased odds of allergic asthma at 10 years of age. Greater fungal maturity at 1 month of age was significantly associated with increased odds of allergic asthma at 10 years of age in females only. We have also shown that that neonatal gut microbiome dysbiosis might promote CD4+ T cell dysfunction associated with childhood atopy.

This adds further evidence that the early microbial environment is important for normal immune development, and that biodiversity losses (on all scales) have implications for long term immune health and the risk of chronic inflammatory diseases.

**Suggested Reading:** Fujimura KE, Sitarik AR, Havstad S, Lin DL, Levan S, Fadrosh D, Panzer AR, LaMere B, Rackaityte E, Lukacs NW, Wegienka G, Boushey HA, Ownby DR, Zoratti EM, Levin AM, Johnson CC, Lynch SV. Neonatal gut microbiota associates with childhood multisensitized atopy and T cell differentiation. Nat Med. 2016 Oct;22(10):1187–1191.

### 8.2. Infant Gut Microbiome and Variations in Functional Brain Activity—Implications for Development and Behaviour

KnickmeyerRebeccaDepartment of Pediatrics and Human Development, Michigan State University, Bioengineering Building, Room 2114, 775 Woodlot Dr., East Lansing, MI 48824, USA

Subtle differences in the colonization of the infant gut microbiome may have lifelong consequences for human mental health. Recent studies of the microbiome-gut-brain-axis in rodents show clear evidence for critical windows in early life. In humans, infancy is the foundational period for establishment of the gut microbiome and the most dynamic phase of postnatal brain development, marked by exuberant synaptogenesis, cortical expansion, and the assembly of functional brain networks.

Our studies examine whether the microbiome affects neurodevelopment in humans during this critical period. To do this we examined 89 Infants who had fecal samples, structural (and/or functional) MRIs, and cognitive assessments at 1 and 2 years of age. We assessed relationships between features of the gut microbiome and cognitive outcomes, brain structure, and functional connectivity. Cognitive performance at 2 years was negatively associated with alpha diversity at 1 year, although there was minimal influence of microbiome on brain volumes. Functional connectivity between the supplemental motor area (SMA) and the inferior parietal lobule (IPL) correlated with alpha diversity at 1 year and cognitive performance at 2 years, suggesting that connectivity between the sensorimotor and default mode networks may be an important pathway linking the gut microbiome to cognitive outcomes in infancy. Functional connectivity between the amygdala and the thalamus and between the anterior cingulate and right anterior insula also correlated with alpha diversity in 1 year olds.

These findings suggest that modulation of gut microbiota may be a tractable strategy for supporting cognitive development and reducing risk for psychiatric disorders. Future studies are needed to determine if the observed associations between the gut microbiome and cognition/functional connectivity are causal and if microbiome-related alterations in connectivity of the amygdala and anterior insula result in altered fear-reactivity. We also need to understand what signaling pathways mediate between gut and brain in human infants, and how we might promote optimal development of the gut microbiome.

**Suggested reading:** Gao W, Salzwedel AP, Carlson AL, Xia K, Azcarate-Peril MA, Styner MA, Thompson AL, Geng X, Goldman BD, Gilmore JH, Knickmeyer RC. Gut microbiome and brain functional connectivity in infants—A preliminary study focusing on the amygdala. Psychopharmacology (Berl). 2019 May;236(5):1641–1651.

### 8.3. Nature-Relatedness and the Infant Microbiome Data from the CHILD Study

Anita Kozyrskyj (Director, inVIVO Planetary Health; PI of the SyMBIOTA project on environmental shaping of the infant gut microbiome, and development of childhood overweight and atopic disease in the CHILD cohort study, University of Alberta, Edmonton AB T6G 2R3, Canada).

The Canadian Healthy Infant Longitudinal Development (CHILD) Study is a major initiative examining how genes and the environment influence child health and development. As part of this, the SymBIOTA program specifically examines the environmental determinants of the infant microbiome in the maturation of the immune system which plays a crucial role in predisposition to multiple diseases in childhood and later life.

Using metadata and infant fecal samples from CHILD we have examined impact of the home environment on infant gut microbiota, including use of cleaning products and pet keeping. For example, we have found that exposure to household furry pets influences the gut microbiota of infant at 3–4 months. We demonstrated a dose response between extent of detergent use and abundance of gut Lachnospiraceae, and that Lachnospiraceae mediates an association between heavy use of home disinfectants and child overweight. This is significant because 80% of Canadian households use a multi-surface cleaner at least weekly, and usage often increases with the birth of a new baby.

More recently we examined the role of proximity to green space and the infant microbiome of CHILD study participants in the Edmonton site using data from the Urban Primary Land and Vegetation Inventory (uPLVI) of Alberta that provides proportion (Focal Statistics) of vegetation. We studied 356 children with 3 month stool samples and postcode-based mapping data and examined associations between gut microbiota composition and natural vegetation close to household residence. We found an increased gut microbial species richness in infants if their families live close to a wetland and increased lactic acid bacteria abundance in the infant gut when families live close to any natural vegetation.

Certainly, how you are born, what you are fed and your exposure to antibiotics shapes your microbiome as an infant. In addition, avoiding heavy-duty disinfectant use, owning a pet, and living close to a green space appear to have favorable effects on the microbiome, which may be a pathway to explain disease protective effects observed in epidemiological studies.

**Suggested Reading:** Tun et al. Exposure to household furry pets influences the gut microbiota of infant at 3–4 months following various birth scenarios Microbiome (2017) 5:40

Azad et al. Impact of maternal intrapartum antibiotics, method of birth and breastfeeding on gut microbiota during the first year of life: a prospective cohort study. BJOG 2016;123

Tun et al. Postnatal exposure to household disinfectants, infant gut microbiota and subsequent risk of overweight. CMAJ 2018;190(37):E1097–E1107

Tun et al. Exposure to household furry pets influences the gut microbiota of infant at 3–4 months following various birth scenarios Microbiome (2017) 5:40

### 8.4. What’s in the Water? Chlorine Levels in Public Drinking Water and Effects on the Assembly of the Infant Gut Microbiome

MartinoDavid^1^ChristophersonClaus^2^PalmerDebbie^1^PrescottSusan L.^1^SilvaDesiree^1^,^3^1The ORIGINS Project, Telethon Kids Institute and University of Western Australia, Nedlands WA 6009, Australia2Edith Cowan University, Joondalup, WA 6027, Australia3Joondalup Health Campus, Joondalup, WA 6027, Australia

This presentation discusses the potential impact of chlorinated public drinking water on the assembly of the intestinal microbiome in infancy. The addition of chlorine or hypochlorite to metropolitan drinking water is routinely used worldwide as a sanitizer because of its potent anti-microbial properties. It is one of the most effective means of delivering safe drinkable water because it produces a residual disinfectant that persists within the distribution system. Levels of chlorine used to treat metropolitan water are considered safe for the individual, based on toxicity studies. However, to our knowledge there have been no studies examining whether levels of persistent chlorine exposure from tap water are also safe for the ecosystem of microorganisms that colonize the gastrointestinal tract. Given the importance of the microbiome in health, persistent exposure to low levels of chlorine may be a hitherto unrecognized risk factor for gut dysbiosis, which has now been linked to virtually every chronic non-communicable disease of the modern era. Although effects may be subtle, young children and infants are more susceptible to ecological disturbance, given that the microbiome is highly influenced by environmental factors during this period.

Here we discuss the “TUMS“ study (waTer qUality and the Microbiome Study), a randomized controlled trial investigating the impact of chlorinated drinking water on the spatio-temporal colonization of the gut bacteria in early childhood.

**Aims:** To recruit 200 unselected families with 6 month old infants from the Joondalup Health Campus catchment area in Western Australia. The study is nested within the ORIGINS birth cohort.

**Methods:** The intervention consists of installing a reverse osmosis carbon block water filter into homes to remove residual chlorine from the public water supply. The control group will receive sham filters according to a 1:1 randomization design. Baseline stool samples will be collected at 6 months and follow up samples collected at 12 and 18 months. Metagenomic analysis will be used to examine changes in the gut microbiome over this period. These data will be examined in the context of water quality measures, lifestyle and environmental variables, and clinical outcomes related to early atopy, eczema, and early wheezing phenotypes.

**Conclusions:** Water source may be an underappreciated determinant of microbiome signatures in early life. This study will provide valuable baseline data regarding the potential effects of chlorine-based disinfectants on developing microbiomes. The findings are likely to be of broad interest to communities, governments, and municipalities alike.

### 8.5. Microbiome Transplants from Asthmatic Versus Non-Asthmatic Children Modulate House Dust Mite Responses in Animal Models

MansfieldLinda S.^1^Moya-UribeIvon^1^TerauchiHinako^1^BellJulia A.^1^ArshadS. Hasan^2^,^3^EwartSusan L.^1^1Michigan State University, East Lansing, MI 48824, USA2University of Southampton, Southampton, UK3David Hide Asthma and Allergy Research Centre, St Mary’s, Hospital, Parkhurst Road, Newport, Isle of Wight, UK

**Aims:** We expect that gut microbiota impacts risk of childhood allergic disease, either directly or indirectly through allergic sensitization. We hypothesized that mice carrying a diverse young adult human microbiota and infected with *Campylobacter jejuni* 260.94 or 11168 that induce an allergy associated, Th2-biased immune response will show exacerbated allergic responses to a known human allergen.

**Methods:** To test this hypothesis, we transplanted germ free mice with human fecal microbiota from young adults by oral gavage. Mice were successfully transplanted with few adverse effects and transferred microbiota vertically to their offspring. Offspring mice with human microbiota (Humice) and mice with mouse microbiota (Momice) were administered *C. jejuni* or sham gavage on ~day −30 and house dust mite allergen (HDM) intranasally on days 0, 2, 5, 7, 9, and 12. On day 14 lung function was measured and blood, bronchoalveolar lavage (BAL), lung, feces, and gastrointestinal tissues collected. Lung inflammation, lung function parameters, and immune responses were assessed.

**Results:** In Humice, either Humicrobiota or *C. jejuni* alone was sufficient to increase plasma Immunoglobulin (Ig)E, while Momice required both HDM and *C. jejuni* to increase IgE. The greatest lung function declines occurred in HDM-sensitized Humice given *C. jejuni*. Respiratory resistance (Rrs) and tissue resistance (G) were significantly increased in HDM exposed, uninfected mice carrying both microbiotas, while Rrs, compliance Crs, and G were affected in *C. jejuni* infected Humice regardless of HDM sensitization. Respiratory elastance (Ers) and tissue elastance (H) were only affected in Humice with HDM and *C. jejuni*. G was affected in *C. jejuni* infected Momice regardless of HDM sensitization, while Crs and Ers were affected only in Momice exposed to both HDM and C. jejuni. Multiple interactions occurred among factors.

**Conclusions.** Human microbiota, with or without concurrent *C. jejuni* infection, enhanced allergic responses to house dust mite in mice. The enteric pathogen, *C. jejuni* to which children are commonly exposed, works together with background microbiota to increase IgE, BAL cells, and airway hyperresponsiveness at physiologic extremes.

### 8.6. Effects of Diet on the Childhood Gut Microbiome and Its Implications for Atopic Dermatitis

Maha Mahdavinia (Allergy/Immunology Division, Internal Medicine Department, Rush University Medical Center, Chicago, IL 60612, USA; her research interest includes pathogenesis of allergic disease, racial differences and the microbiome).

Microbes are protective against allergic diseases. The protective effect of living on a farm against the development of allergy and asthma has been known for decades. This protective role has been suggested to occur at least partially via exposure to a more diverse group of microorganisms in the environment. Development of a healthy microbiome is dependent on the individual’s exposure to a surrounding environment rich in microbes.

Bacteria from farms reduce allergic reaction. In mouse models, certain bacteria, including ones that were isolated from farm environments, show a significant reduction in the allergic inflammatory reaction in lungs. These bacteria include *Acinetobacter lwoffii, Lactococcus lactis, Staphylococcus sciuri,* and *Bacillus licheniformis.*

Gut microbiome change is proposed as a possible contributor to the increase in allergy. Diversity of microbial exposure is inversely correlated with development of allergic and inflammatory conditions such as asthma and allergic rhinitis. An environmentally induced alteration of the commensal microbiota in gut might be driving the rapidly increasing prevalence of allergic responses to food in Western societies, but what is the effect of diet in the microbiome change?

We examined this in an African population. Although the prevalence of atopic diseases is lower in Africa, individuals of African origin who live in Western countries are at a significantly higher risk for atopic conditions. This possibly reflects an enhanced genetic predisposition to allergy that is kept in check by their ancestral environment. In certain areas of Africa, people still live in close daily contact with animals and plants in natural settings. Therefore, determining how their natural environment or diet protects these potentially at-risk populations should shed light on causes of the global rise in allergic conditions.

Patients and controls were recruited from rural areas of Cape in South Africa. This includes Black South African (Xhosa) children from the remote rural Mqanduli district of the Eastern Cape. Patients with atopic dermatitis (AD) were recruited from the dermatology department of the Nelson Mandela in the Eastern Cape. Control (non-allergic, non-food-sensitized) subjects were recruited from the areas surrounding 10 district community health clinics. Eighty-three children were recruited; 36 with AD and 47 controls without AD. All patients were tested for a panel of food and aeroallergens. AD was significantly associated with food sensitization, but not with allergic rhinitis or wheezing.

Rural children had a significantly more diverse microbiota that clustered separately from urban children, and the relative abundance of *Prevotella copri* is significantly higher in rural children. This was in genetically identical groups. We found that sugar and fat content in early life diet were associated with shifts in the gut microbiome. High sugar intake was associated with decreased diversity of gut microbiome. High fat intake was associated with decreased relative abundance of *P. copri*. Toddlers with AD had a significantly higher daily consumption of total sugar and saturated fat than controls, and associated differences in beta diversity of gut microbiome.

In conclusion, while there are many reasons for lower rates of allergic disease in rural South African children this may be in part due to effects of rural diets, which have significantly less fat and sugar, on the microbiome.

### 8.7. Investigation of External Microbial Exposures and Child Cognition and Behaviour at 2 Years of Age

SennElizabeth^1^,^2^SymeonidesChristos^1^,^3^VuillerminPeter^1^,^2^,^4^PonsonbyAnne-Louise^1^,^3^the the Barwon Infant Study Investigator Group^5^1Murdoch Children’s Research Institute, Flemington Rd, Parkville, Victoria, 3052, Australia2Deakin University, Geelong, Victoria 3220, Australia3Department of Paediatrics, University of Melbourne, Grattan St Parkville, Victoria, 3052, Australia4Barwon Health, University Hospital, Bellerine Street, Geelong, Victoria 3220, Australia5John Carlin, Mimi Tang, Katie Allen, Fiona Collier, Terry Dwyer, Sarath Ranganathan and David Burgner

**Background:** Epidemiological studies have identified a range of environmental factors associated with higher microbial exposure in early life and reduced risk of allergic disease. Several of these exposures impact the microbial composition of the infant gut. Despite intense interest in the influence of gut microbiome on early cognition and behaviour, the relationship between these external microbial exposures and cognition and behaviour has not been well characterised.

**Method:** The Barwon Infant Study is a birth cohort (*n* = 1074) in Victoria, Australia. Comprehensive questionnaire, clinical and biological measures were collected at multiple time-points. Multiple linear regression was used to evaluate associations between 56 external microbial exposures and three outcomes; cognition (Bayley Scales of Infant and Toddler Development (BAYLEY-III)) (*n* = 667, mean (SD) age = 2.45 (0.14) years), internalising, and externalising behaviour (Child Behaviour Checklist (CBCL)) (*n* = 666, mean (SD) age = 2.45 (0.14) years).

**Results:** Overall, there were no consistent patterns or dose response found within an outcome nor across all three outcomes, although there was some evidence for individual associations. Breastfeeding and child care were associated with higher cognitive scores (adj. mean diff. (95% CI) = 3.20 (0.23, 6.17) and 0.68 (0.12, 1.24) respectively), and increasing sibling number was associated with lower internalising behaviour (adj. mean diff. (95% CI) = −4.13 (–6.34, −1.91)).

**Conclusion:** In contrast to allergic disease, there was an absence of epidemiological evidence to support the association between these external microbial exposures and cognition and behaviour. Further studies are required to investigate a broader sweep of exposures which influence gut-microbial composition and cognition and behaviour.

## 9. Mindsets Matter: How Attitudes and Beliefs Can (Re)Shape Personal and Planetary Health

This session addressed the importance of positive emotional assets for physical and emotional health. In addition to the role of personal mindsets, these discussions also underscored the importance of promoting collective ‘planetary mindsets’ to advance concepts of planetary health.

### 9.1. The Magic of Hope: Hope Mediates the Relationship between Socioeconomic Status and Opportunity in the Next Generation

Dante D. Dixson (Educational Psychologist, Wayne State University, Detroit, MI 48202, USA; Principal Investigator of the Hope Laboratory; expertise in the role of hope and positive psychology in the educational and psychological functioning of at-risk children and adolescents).

“Looking towards the future with positive expectations is a powerful force on the present”

―Dr. Dante Dixson

Hope has enormous potential as a “game changer” in schools and life in general. Researchers should study it. Schools should leverage it. Everyone should believe in it!

Perceptions are powerful and greatly determine performance and success. Twin studies demonstrate that socioeconomic status modifies heritability of IQ in young children. In impoverished families, 60%–80% of the variance in IQ is accounted for by the shared environment, and the contribution of genes is close to zero, whereas the opposite is true in affluent families. Perceived social position is associated with higher achievement, high self-efficacy, high school belonging, college rates, and lower dropout rates. “Psychosocial trajectory” has a powerful influence on student outcomes in the short and longer term. Importantly, this can be modified by simple, cheap interventions. Social-psychological interventions have been tested in educational settings. These target students’ perception of their environment, reinforcing that even when environments cannot be easily changed, perceptions can. These have repeating effects, and can be highly effective and very little cost. Even a one hour lab intervention on college campuses resulted in higher GPAs from sophomore through senior year, fewer absences, and fewer behavioral referrals. Three years later, these students were happier and healthier.

The Hope Laboratory was established to inspire students to envision and believe in a better tomorrow and to build an evidentiary framework around this. A core belief of our lab is that many students have an inequitable chance at academic success due to factors beyond their level of control (e.g., poverty, stereotypes, racism). As a response to this belief, the mission of this lab has three parts. The first is to research and better understand how psychosocial factors (i.e., students’ thoughts, attitudes, and beliefs) are associated with improving the achievement of minority and disadvantaged youth. The second is to use the best research available on the relationship between psychosocial factors and achievement to inform quick and effective perception-based interventions that can be implemented universally in schools to give more students at chance at academic success. The third is to study and better understand the factors associated with the identification of minority and disadvantaged students for gifted and talented programs (GATE) so that those students have a more equitable opportunity to develop their gifted abilities and live up to their academic potential.

Our main focus is hope, the ability to believe in a better tomorrow, irrespective of current circumstances, as well as belief, and the corresponding motivation to get there. Hope has been found to be both a powerful concept and influential predictor for children and adolescent populations, with research indicating that hope is meaningfully related to improvements in happiness, health, and academic success. The Hope Laboratory researches the various factors that lead to increasing hope in students, better understanding how hope leads to increased academic success in students, and quick (<90 minutes) hope-based interventions that can be implemented universally in schools.

An additional focus of The Hope Laboratory is gifted minority and disadvantaged students. Although the focus of a lot of scholarship, most studies indicate that minority and disadvantaged youth are vastly underrepresented in GATE programs compared to their European American and Asian counterparts, indicating that many minority and disadvantaged young people are not getting the opportunity to live up to academic potential. In response, The Hope Laboratory researches how psychosocial factors relate to the likelihood of minority and disadvantaged youth being identified to GATE programs as well as what actions that can be carried out by potentially gifted students and parents that increase the likelihood of gifted minority and disadvantaged students being identified and gaining entry into GATE programs.

The final focus of The Hope Laboratory is psychosocial factors and the achievement gap. Although the primary focus of the lab is hope, the lab also examines how many additional, related psychosocial factors can both increase the achievement of minority and disadvantaged youth and close the achievement gap. This research sheds light on how student perception plays a role in the achievement gap and more importantly, how that perception can be altered via interventions to close the achievement gap.

**Suggested Reading:** Walton GM1, Cohen GL. A brief social-belonging intervention improves academic and health outcomes of minority students. Science. 2011 Mar 18;331(6023):1447–1451

Dixson, D. D. Is grit worth the investment: How grit compares to other psychosocial factors in predicting achievement. Current Psychology (in press).

Dixson, D. D., Anderson, C., & Keltner, D. (2019). Measuring positive emotions: An examination of the reliability and structural validity of scores on the Seven Dispositional Positive Emotions Scales. Journal of Well-Being Assessment. *2018, 2, 3;* 115–133

Dixson, D. D. & Stevens, D. (2018). A potential avenue for academic success: Hope predicts an achievement-oriented psychosocial profile in African American adolescents. Journal of Black Psychology 44, 532–561.

### 9.2. Beyond Fear: The Mental Health Impacts of Climate Change and Ecological Change and What It Suggests as a Path Forward

Blake Poland (Dalla Lana School of Public Health, University of Toronto, Toronto, ON M5T 3M7, Canada; his work investigates critical social theory for healthy cities and communities including social movement as agents of social change, dialogical methods, and arts-informed research for community resilience).

The mental health impacts of climate change and ecological degradation have only recently been more fully appreciated for their capacity to overwhelm an already stretched and underfunded mental health ‘system’. A level of media coverage of climate change and environment that many consider inadequate, an almost-daily ‘sounding of the alarms’, and our own deep interconnectedness with nature, is resulting in epic levels of anxiety, depression, and despair in response to the mounting devastation of our natural home, in addition of course to bearing some fruit in response to the ‘call to action’ that ‘sounding the alarm’ was meant to convey. An improvement in our capacity to understand and respond to the mental health impacts of climate change and ecological degradation is therefore called for. This paper explores the possibilities for meaningful response on three fronts. First is the need to more fulsomely incorporate mental health considerations into existing and future climate change and health vulnerability and adaptation assessments. Here we present recently published work that offers a framework for doing so. Second, we suggest that a key, and hitherto largely overlooked, tool for community resilience building on these issues is the formation of small-scale place-based dialogue circles akin to what Lerner calls ‘Circles of Compassion’ or what the Transition Town movement has spawned as ‘Heart and Soul of Transition’ groups, as part of a larger civil society and social movement response that complements rather than displaces more formal sector services and programs. Third, given the importance of the stories, we tell ourselves and others about who and where we are, and where we are headed, and the capacity of these stories to become self-fulfilling prophecies, we advocate for the culture-change work of crafting a new story to live by that attunes us to the powerful potential we all embody to co-create a more beautiful, just, and sustainable world.

**Suggested Reading:** Hayes K, Poland B. Addressing mental health in a changing climate: Incorporating mental health indicators into climate change and health vulnerability and adaptation assessments. Int J Environ Res Public Health. 2018;15(9).

Patrick R, Dooris M, Poland B. Healthy Cities and the Transition movement: converging towards ecological well-being? Glob Health Promot. 2016 Mar;23(1 Suppl):90–93.

Benatar S, Poland B. Lessons for Health from Insights into Environmental Crises. Int J Health Serv. 2016 Oct;46(4):825–842.

### 9.3. The Importance of Art in Personal and Collective Mindsets: Benefits for Community Building, Activism and Outcomes

Holly Feen-Calligan (College of Education Art Therapy Program, Wayne State University, Detroit, MI 48202, USA. Her interests include the value of art in personal and community health. She received the 2015 American Art Therapy Association’s Distinguished Service Award).

Sketching, doodling, and making handcrafts or fine art help people to connect with a side of themselves—a spiritual, creative side, that is essential for self-knowledge and wellness. Similarly, working in close proximity with others while painting a mural or weeding a community garden allows for conversation and discovery of common and diverse interests.

This presentation focuses on ArtsCorpsDetroit, a community engagement program of Wayne State University in which students and community members participate together in curricular service-learning experiences and/or as volunteers working on neighborhood art projects—both serving to enliven public spaces and build skills and relationships. ArtsCorpsDetroit’s “Lots of Art” initiatives have contributed to rebuilding the City of Detroit following its emergence from bankruptcy in 2014, through supporting urban garden initiatives, and rehabilitation of former residential or business properties, now empty. When neighborhood groups request assistance with reclaiming empty properties, ArtsCorpsDetroit helps transform empty lots into “Lots of Art”: community gardens, sculptures, performance stages, or murals.

The presentation highlights specific service-learning and volunteer community art projects of ArtsCorpsDertoit, demonstrating the benefits of collective, creative action to personal and collective health. For example, data collected from university service-learning courses indicate benefits to constituents: students gain inter- and intra-personal skills and self-determination for social responsibility, agencies increase their programming capacities, and the university’s relationships with its neighbors are strengthened (as per the suggested reading below).

Community gardening and art-making foster personal and social identity, and social relationships and responsibilities to one another and to the environment become stronger. Where people have experienced the collective trauma of poverty, unemployment, business closures, etc., opportunities to reconstruct their histories and to dialog together about an imagined better future is health promoting. Community mural projects allow for the expression of histories and dreams.

**Suggested Reading:** Boggs, G. L. (2011). The next American revolution: Sustainable activism for the twenty-first century. Berkeley, CA: University of California Press.

Feen-Calligan, H. & Matthews, M. (2016). Pre-professional arts-based service-learning in music education and art therapy. International Journal of Education and the Arts, 17(17).

Lawton, P. H. (2010, November). Hand-in hand, building community on common ground. Art Education, 63(6), 6–12.

Watkins, M. & Shulman, H. (2008). Toward psychologies of liberation. New York, NY: Palgrave MacMillan. ISBN-13: 978-0230537699

### 9.4. Microbes and Mindsets: Empathy and Altruism Associated with Beneficial Microbes—More Societal Implications of ‘Dysbiotic Drift’?

Susan Erdman (Head, Erdman Lab, Division of Comparative Medicine, Massachusetts Institute of Technology, Cambridge, MA 02139, USA; her research investigates how bacteria and inflammation contribute to systemic health and diseases).

This presentation explores the links between microbes, altruism, purposeful lives, and the central role of oxytocin in the biological pathways that promote healthful longevity through social interactions. Oxytocin is best known as the “love hormone”. It builds love and trust, bonds families, enhances sociability, improves wound healing, and relieves stress—all factors associated with both healthy beginnings and healthy aging.

With growing evidence that the commensal gut microbiota promotes healthy aging, there are mounting concerns about the broader impact of changing microbiota (with western environments and western diets) on all aspects of wellbeing. This is already implicated in the epidemic increase of inflammatory disorders later in life. Is it also implicated in the documented decreases in empathy and social cohesion? Has this “changed what it means to be human”?

Dietary microbes have been shown to alter behavior in animal models. In mice, probiotics promote skin healing and bias stress responses with significant increases in plasma oxytocin levels, and reciprocal reductions in plasma corticosterone. Probiotics also increase oxytocin positive cells in the brain using immunohistochemistry.

This raises the question of if replacing ‘missing’ microbiota, in addition to other effects, may also stimulate empathy and altruism as part of adaptive personal and social responses to environmental challenges? We propose that adding novel oxytocin (OXT) stimulating bacteria at birth stimulate oxytocin release and promote impulse control, altruism, empathy, and even spirituality.

We are particularly interested in *Lactobacillus reuteri* in this context. The rationale is that this organism is becoming depleted in modern societies. It is a human breast milk-source microbe and a model microbial symbiont with mammalian evolutionary relevancy. It has a history of clinical efficacy in humans and is easy to cultivate and aerotolerant. It also ferments a wide range of substrates.

New human randomized controlled trials are currently assessing effects of maternal probiotics on novel outcomes in such as emotional recognition, maternal–infant and father–infant bonding, general attachment style, altruism, empathy, perceived social support, impulse control, resilience, wellbeing, and spirituality in addition to microbiome, immune function, hormone regulation, stress responses, and other biological measures.

The evolving understanding of the effects of microbiota on stress, mood, emotion, and behavior provide novel perspectives on the role of our symbionts in the “trajectory of purpose” in human evolution. As microbes are so important for boosting host survival and thrift, stimulating reproductive fitness, promoting pro-social behaviors, and building social networks, we may well ask “who is driving this bus?”

### 9.5. Mindsets Matter: Utilizing the Power of Placebo

NelsonDavidIndependent early career researcher with an interest in mind-body medicine, clinical ecology and placebo studies. Woodstock, ON N4S 6Y9, Canada

The “the placebo effect” is measure of the power of mindsets, specifically the extent to which beliefs, expectations, and agency can influence virtually all aspects of health. Yet prevailing perspectives of “the placebo” rarely capture the true potential or significance of this effect as a therapeutic solution for health on all scales. Placebos are commonly considered as “a harmless, pill, medicine, or procedure prescribed more for the psychological benefit to the patient than for any physiological effect; a substance with no therapeutic value a measure designed merely to calm or please someone”, or “a bodily change due to the symbolic effects of a treatment or treatment situation and not due to its pharmacologic or physiologic properties”. While well recognized, “placebo effects” have been primarily used for “subtracting” these otherwise unexplained generic benefits of mere expectation from the impact of “active” test treatments in clinical trials. Outside complementary medicine, little attention has been paid to understanding or utilizing the psychological and physiological “power of the placebo” at the individual level. Even less attention has been given to the collective power of expectations and beliefs on communities and societies. This presentation explores why “mindsets matter” for both personal and planetary health, and why narrative approaches must be a key part of every solution.

Decades of research clearly demonstrate how attitudes, circumstances and associated narrative can influence numerous aspects of individual health, with implication for stress responses, inflammation, and aging. No less important is an understanding of the ways in which stories contribute to what ails person, place, and planet. It can be argued that “planetary mindsets”—which determine collective attitudes, values and behavior towards the environment—are also a crucial dimension of promoting planetary health. As such, the principles and practice of narrative medicine can be applied on a larger scale, to the grand interconnected challenges of our time one. Indeed, personal, community, and global health narratives are already melding with powerful narratives set by commercial entities which drive consumerism and downplay environmental damage and the wider implications for health. Providing a powerful counternarrative is as essential part of shifting the normative values that dominate the Anthropocene and undermine the health of person, place, and planet.

The success of planetary health as a new concept will be strengthened by attention to the ways in which storytelling can influence positive change. Before we can “exit the Anthropocene” and enter the Symbiocene we must think differently, we must change our mindset first.

**Suggested reading:** Susan L. Prescott and Alan C. Logan. Narrative Medicine Meets Planetary Health: Mindsets Matter in the Anthropocene Challenges 2019, 10(1), 17

David H. Nelson, Susan L. Prescott, Alan C. Logan and Jeffrey S. Bland. Clinical Ecology—Transforming 21st-Century Medicine with Planetary Health in Mind. Challenges 2019, 10(1), 15;

### 9.6. Promoting Pro-Environmental and Pro-Social Mindsets: How Young Can We Start?

GibsonLisa^1^SilvaDesiree^1^,^2^SobkoTanja^3^HagemannErika^1^DavisJacqueline^1^PrescottSusan^1^,^4^1The ORIGINS Project, Telethon Kids Institute, University of Western Australia, Perth, WA 6009, Australia2Joondalup Health Campus, Joondalup, WA 6027, Australia3The University of Hong Kong, Hong Kong, China4Perth Children’s Hospital, Perth, WA 6009, Australia

A significant shift towards less healthy childhood lifestyle behaviors is reflected in the recent rise in lifestyle diseases, such as obesity and overweight, now affecting one in four Australian children. There are many contributing unhealthy behaviors, including a lack of physical activity, screen time and other passive sedentary activities, poor nutrition, and insufficient quality and/or quantity of sleep. These have been driven by changes in the modern environment and patterns of leisure activities of families and young children. In particular, the shift away from active, outdoor nature-based activities is implicated as a foundational factor driving many of these any of these other risk factors, and increasingly implicated in the parallel rise in psychological and emotional disorders and chronic diseases across the life course.

There has been a striking decline in outdoor play among Australian children, despite the tradition Australian ‘outdoor culture’. This has fundamental implications for both individual and societal health. Environmental cues and time spent in nature are key determinants of health and health behaviors including food choices and physical activity. Nature contact also promotes emotional assets, environmental concern, and prosocial attitudes. It has implications for healthy microbial diversity and immune and metabolic health. Collectively, these converging pathways contribute to the beneficial effects of ‘nature play’ on the physical health and emotional wellbeing in children.

Given that health behaviors are established early in life, it is vital that interventions to promote healthy lifestyles start as early as possible. There is a growing body of evidence demonstrating the benefits of connectedness to nature on a child’s physical and emotional health. There is also preliminary evidence that interventions to induce connectedness to nature result in positive changes in the key areas of nutrition, physical activity, sedentary behavior, and emotional wellbeing in children and their caregivers.

The ORIGINS Project is a community-based interventional birth cohort (of 10,000 children and their families) with the primary goal of improving both the short and long-term health and wellbeing of young children. One of the proposed activities will be refining and testing a new intervention aimed at promoting healthy eating, physical activity, emotional wellbeing, and connectedness to nature in pre-school children (2–4 years). It is anticipated that this in turn will lead to the establishment of a healthy lifestyle early in life and better long term physical and emotional wellbeing for these children and their families.

## 10. An Experiential Workshop: Integrative Imperatives for Planetary Health Research and Reality

Our dynamic facilitators led interactive discussions for ‘action’ planning for personal and planetary change. This interactive workshop utilized arts-enabled methods to activate our senses and intellects. We explored the challenges in the spirit of building an inclusive vision for planetary health that leverages diverse communities and approaches to knowledge production and advocacy.

Facilitators:

Chris Buse (University of British Columbia, Vancouver, BC V6T 1Z4, Canada) investigates how to incorporate the ecological and social determinants of health into environmental assessment processes, and responses to the health impacts of environmental change).

Jordan (Sky) Oestreicher (Université du Québec à Montréal, Montréal, Quebec H3C 3J7, Canada) is a researcher and an artist, with interests in social-environmental justice, participatory development, and community health. She is an international consultant on climate change adaptation projects and land tenure strengthening programs across Latin America and the Caribbean.

Chris and Jordan led this exploratory, experiential learning module to address the “so what/now what” of planetary health research and practice. The goal was goal of fostering a dialogue about inclusivity in the Planetary Health realm, through small group activities.

We used a generative brainstorming excise to map the terrain frontiers/boundaries and opportunities for change. Each group developed a ‘tableau’—intended as a dramatization in the form of a ‘still life photo’—to speak inclusively and integratively to incorporating various diverse dimensions into planetary health research, advocacy, and action (e.g., environment, community, health; qualitative, quantitative; health of humans, animals and ecosystems).

Most groups extended this to create a series of dynamic stories with moving parts!

After the meeting our facilitators will synthesize key themes towards integrative imperatives for environmental health research in the age of the Anthropocene. Stay tuned!

**Suggested reading** (used to frame the synthesis and discussions): Oestreicher JS, Buse C, Brisbois B, Patrick R, Jenkins A, Kingsley J, Tevora R, Fatorelli L. Where ecosystems, people and health meet: Academic traditions and emerging fields for research and practice. *Sustainability in Debate*, 2018; 9(1), 45–65.

Buse CG, Oestereicher JS, Ellis N, Patrick R, Brisbois B, Jenkins AP, McKellar K, Kingsley JY, Gislason M, Galway LP, Macfarlane R, Walker J, Frumkin H, Parkes M. A public health guide to field developments linking ecosystems, environments and health in the Anthropocene. *Journal of Epidemiology and Community Health*, 2018; 72(5), 420–425.

## 11. Unravelling the Exposome: Applying the Biology of Complexity to Tailored Solutions for Person and Place

This session addressed the multi-dimensional complexities of the ‘total lived experience’ in determining human relationships with the environment and determinants of life long-health. It also included discussions of how to handle the complex data systems required to understand this, and the potential and pitfalls of personalized medicine in the 21st century. There were also examples of novel interventions for human and environmental health.

### 11.1. KEYNOTE: Next-Level ‘Total Exposome’ Research: Getting to the Root Problems for the Future of Personal and Planetary Health

Robert O. Wright is a pediatrician, medical toxicology and environmental epidemiologist; Founding Director of the Mount Sinai Institute for Exposomic Research; Ethel H Wise Chair of Environmental Medicine and Public Health, Icahn School of Medicine at Mount Sinai, New York, NY 10029, USA.

The promise of Precision Medicine brings new dimensions to the future of health and medicine. This has provided new dimensions of tailored ‘personalized’ therapies, including drug efficacy and toxicity (pharmacogenomics) based on genetic differences. However, it is becoming increasingly clear that genetics is only one piece of a much bigger puzzle, and many Precision Medicine programs have not considered the ‘personalized’ dimensions of the environment—the “exposome”.

The exposome is a unique complex interplay of variable and equally individualized exposures such as nutrition, microbiomes (and infections), chemicals, physical environments culture/society and a wide range of stressors. Understanding the personalized contribution of the exposome (in the context of genetics) is arguably of greater significance in the context of complex diseases such as ADHD, obesity, asthma, COPD, Parkinson’s disease, cancer, and many other chronic conditions. Yet, the “genocentric’ focus of Precision Medicine has placed lesser emphasis on conditions that have been perceived to be “environmental” conditions.

In reality all conditions have a genetic and environmental component, but there is an artificial and over-simplistic distinction based on perceived contribution of “Nature vs. Nurture”. When a genetic polymorphism is low in prevalence and an environmental factor high in prevalence the disease appears “genetic”. Conversely, if an environmental factor is at low prevalence and genetic polymorphisms are high in prevalence the disease appears “environmental”.

The oftentimes “genocentric” focus Precision Medicine appears to reflect the contrasting historical agendas of “medicine” and “environmental and public health”. Medicine is focused in the “individual” level diagnosis (certainty a problem exists), treatments, side effects and outcomes. Medicine starts after the person is sick, when there is less focus on “why” and more focus on “how” to treat the situation that already exists. On the other hand, public health starts before disease develops and is concerned with population level risk environmental factors (probability of illness) and more focused on discovering “why” and how to prevent in susceptible populations.

Exposome research offers an opportunity to provide a far more comprehensive approach to both health and disease, by applying the same precision approaches and technologies to understand environment impacts across the course of life, from presymptomatic risk trajectories, diagnosis, treatment variability, side effects and disease progression.

In the future, exposomics will enable larger and larger numbers of environmental factors to be measured and analyzed as a “mixture”. This is closer to “real life” and can be incorporated into precision medicine/health for both prevention and treatment—to improve precision. It also has the potential to go back in time and discover critical windows for early intervention and complete the complex disease puzzle.

### 11.2. The Secret World of Your Data: Using Latent Variables to Find Precision in the Complex

Alexandra Sitarik is a biostatistician primarily working in the fields of asthma, allergy, and obesity at Henry Ford Health System, Detroit, MI 48202, USA. Her specialities include the analysis of microbiome data, latent variable methods, longitudinal data analysis, network analysis, and mediation analysis.

Precision Medicine allows us to create tailored solutions for “person and place”. New technologies allow us to identify patterns within large numbers of variables (for example hundreds, or even thousands of different immunological or metabolic markers) that might contribute to a particular disease or phenotype (for example obesity or asthma). One way to think of these subgroups of patterns is as latent variables—essentially, we cannot directly measure these causal constructs, but we can infer them from other measurable variables (“observed/manifest variables”). By this model, individuals in these subgroups should be similar to one another, but distinct from individuals in other subgroups.

Latent variables can also take on several different forms and distributions, not necessarily just categorical subgroups. In essence, this is a model-based way to do data reduction. Latent variables originated in the social sciences, so it has classically been used to model human behavior (such as distilling characteristics to the big five personality traits). This approach has had wide application in psychology to understand leadership styles, in education to explore teaching styles, and in sociology to understand the complex determinants of socioeconomic status. However, it is applicable to many research questions and fields of study and is increasingly being applied to public health problems.

This can provide a more precise view and interpretation of data. Researchers often rely on variables that are imperfect approximations of the constructs they care about. Typical regression models treat all variables as if they were perfectly reliable (i.e., no measurement error), but this is rarely the case, and failing to account for measurement error in causal models can have potentially serious consequences for both loss of power and inflation of estimates. Latent variable methods allow us to build measurement error into our models.

The method is primarily a statistical tool built around structural equation modeling (SEM) which allows us to analyze the structural relationship between measured variables and latent constructs. The model is selected based on whether it does a good job of fitting the observed data. This is also based on the main assumption of local independence that the observed items are conditionally independent of each other given the latent variable, i.e., the latent variable explains why the observed items are related to another.

Other advantages include the capacity to accommodate missing data (assumes MAR), incorporate covariates, interactions (“measurement invariance”), weighting, and outcomes into modeling procedure. Subtyping asthma provides a good example. Clinically (phenotypically), this is already recognized not to be a single disease, but a heterogeneous umbrella term for multiple distinct diseases with different underlying mechanisms. Asthma phenotypes are subtypes which differ based on observable characteristics, whereas asthma endotypes are subtypes associated with underlying disease mechanisms. Identification of these subtypes may result in a more targeted approach to prevention and treatment. These are hypothetical constructs: latent variables that can be inferred from observed clinical and biological parameters.

In the Columbia Center for Children’s Environmental Health (CCCEH) longitudinal birth cohort study, Chen et al. (2012) applied these methods to identify four phenotypes of wheeze using latent class growth analysis (LCGA) from longitudinal questionnaire data. They found that maternal asthma, ethnicity, and child sex associated with wheeze phenotype, and that the effects of viral infections (the “flu season”) on wheezing depended upon wheeze phenotype.

Similarly, we previously identified atopic phenotypes in the WHEALS birth cohort study (Havstad et al. 2014). Although atopy is the most commonly reported risk factor for asthma, the “atopy” umbrella term may obscure more specific allergic disease subtypes. We applied LCA to 10 allergen-specific IgE measurements at age two and found that the resulting four atopic phenotypes identified through LCA were better at distinguishing atopic dermatitis, wheeze, and asthma, compared to the conventional definition of atopy.

We have also applied this to dietary patterns in the WHEALS birth cohort to explore how maternal and infant eating behaviors begin to take shape in utero and continue to develop throughout infancy. Breastfed children have been shown to be more open to trying new foods and accepting them in infancy, but little is known whether this affect persists into adolescence. We examined Food Frequency Questionnaires administered to children at 10 years of age and attempted to identify underlying dietary patterns (DPs), rather than focusing on any one particular food. Three dietary patterns were identified using LCA at age 10; we found that breastfed children were less likely to have a DP characterized by a high consumption of processed and energy dense foods and more likely to have a DP characterized by “healthy” foods.

In conclusion, latent variable modeling is a great way to reduce complex data, identify meaningful underlying patterns, and account for measurement error. There are many variations in modeling procedures that allow for a variety of data distributions and hypothesized latent variable distributions. These methods are particularly useful where tailored solutions are needed for prevention, diagnosis, or treatment response and management. I encourage you to think beyond the data you did collect, to the data you would have collected if you could have! There are probably latent variables “hiding” in your datasets right now that you have never considered, just waiting to be found.

### 11.3. Newborn Epigenetic Effects of L. Reuteri Probiotic in Pregnancy: Role of Epigenetics in Prediction and Prevention?

Maria Jenmalm is Professor of Experimental Allergology at Linköping University, 581 83 Linköping, Sweden. Her main interests are in childhood immune maturation and allergy development, including epigenetic regulation by maternal immunity and microbial exposure during pregnancy.

Anna Forsberg ^1,^*, Johanna Huoman ^1,^*, Simon Söderholm ^2^, Ratnesh Bhai Mehta ^1^, Lennart Nilsson ^3^, Thomas R. Abrahamsson ^4^, Jan Ernerudh ^5^, Mika Gustafsson ^6,†^ and Maria C. Jenmalm ^1,†^

^1^  Division of Neuro and Inflammation Sciences, Department of Clinical and Experimental Medicine, Linköping University, Linköping, Sweden
^2^  Division of Medical Microbiology, Department of Clinical and Experimental Medicine, Wallenberg Centre for Molecular Medicine, Linköping University, Linköping, Sweden
^3^  Allergy Centre, Department of Clinical and Experimental Medicine, Linköping University, Linköping, Sweden
^4^  Department of Paediatrics, Department of Clinical and Experimental Medicine, Linköping University, Linköping, Sweden
^5^  Department of Clinical Immunology and Transfusion Medicine, and Department of Clinical and Experimental Medicine, Linköping University, Linköping, Sweden
^6^  Bioinformatics, Department of Physics, Chemistry and Biology, Linköping University, Sweden
* shared first authorship
^†^  shared senior authorship.

The importance of early microbial exposure for immune development has been the basis for numerous probiotic supplementation studies aimed at reducing the risk of eczema. Collectively, these studies suggest differential effects of interventions in the prenatal versus the postnatal period, with stronger preventive effects of combined pre- and postnatal period, with stronger preventive effects of prenatal supplementation for eczema prevention. Data from 2757 mother–baby pairs (pooled data from 11 studies) show significantly lower risk of eczema (OR 0.54; 95% CI 0.50–0.59; *p* < 0.001) when probiotics were given prenatally (number needed to treat = 12) compared with data from 638 mother–baby pairs (pooled data from four studies). Studies that give only postnatal supplementation that failed to show a significant effect (OR 0.89; 95% CI 0.59–1.35; *p* = 0.59; number needed to treat = 50). This highlights the importance of the timing of allergy preventive immunomodulatory dietary interventions in the search for optimal strategies.

We speculate that antenatal exposure is important for anticipatory modulation of offspring immunity, and that this is mediated via epigenetic mechanisms in response to prenatal microbial immunomodulatory factors. We propose that postnatal administration in the absence of antenatal exposure creates a scenario of environmental “mismatch” (i.e., the anticipated environment is different from the encountered environment), and that this may contribute to immune dysregulation and increased predisposition to allergy.

We explored this by examining epigenetic effects of antenatal *Lactobacillus reuteri* supplementation using samples from a previous allergy prevention study. In the original randomized controlled trial, the effect of *L. reuteri* ATCC 55730 (10^8^ CFU/day in oil drops) given from given from 36 weeks gestation until 12 months of age (n = 95), was compared with a placebo (n = 93). Clinical follow up at 24 months showed reduced IgE associated eczema, but at 7 years there were no effects on asthma, rhinitis, or eczema. In this new analysis, we performed epigenetic analysis on blood samples collected at birth (cord blood), 12 and 24 months of age. We isolated CD4+ T cells from mononuclear cells, extracted DNA, and performed bioinformatic analyses on the global DNA methylation patterns (measured by Illumina Infinium 450K array).

We found differential methylation at birth in probiotic compared with placebo treated infants. Network analyses revealed immune-associated gene modules, including chemokine signalling pathways, TGFβ signalling, and PI3K-Akt and MAPK signalling. We performed visualization of the probiotic module network in relation to development of atopic manifestations to demonstrate the allergy predictive power of the probiotic module.

Our findings provide preliminary evidence that the protective effects of prenatal probiotic supplementation on eczema prevention may be partially mediated by epigenetic effects. This adds further evidence that antenatal microbial exposure is important as also suggested by the allergy protective effects of antenatal farm exposure in observational studies. Our intervention did not commence until later in gestation, underscoring the need to investigate the effects of much earlier interventions.

In conclusion, further studies are required to examine the potential of probiotics for allergy prevention, but at this stage, combined pre- and postnatal supplementation seem important. Maternal *L. reuteri* supplementation during pregnancy may alter DNA methylation, and patterns in CD4+ T cells towards enhanced immune activation at birth. New studies with probiotic supplementation earlier in pregnancy are now underway to explore the effects on immune maturation and allergy development.

**Suggested reading:** Jenmalm MC: The mother-offspring dyad: microbial transmission, immune interactions and allergy development. J Intern Med 2017; 82: 484–495

### 11.4. The Opportunities and Challenges of Early Life Interventions for Disease Prevention in Japan

Naoki Shimojo (Department of Pediatrics, Graduate School of Medicine, Chiba University, Chiba, 263-8522, Japan)

No abstract available.

### 11.5. Design and Application of a Multi-Strain Insect Probiotic to Address Global Honey Bee Population Decline

DaisleyBrendan A.^1^,^2^ChmielJohn A.^1^,^2^TrinderMark^1^,^2^PitekAndrew P.^3^ChernyshovaAnna M.^3^SumarahMark W.^3^,^4^BurtonJeremy P.^1^,^2^,^5^ThompsonGraham J.^3^ReidGregor^1^,^2^,^5^1Centre for Human Microbiome and Probiotic Research, Lawson Health Research Institute, London, N6C 2R5, Canada2Department of Microbiology and Immunology, The University of Western Ontario, London, N6A 5C1, Canada3Department of Biology, The University of Western Ontario, London, N6A 5C1, Canada4London Research and Development Center, Agriculture and Agri-Food Canada, London, N5V 3V3, Canada5Department of Surgery, The University of Western Ontario, London, N6A 4V2, Canada

Abstract (please use 350 words as a guide, but we are flexible)

Persistent global decline in honey bee (Apis mellifera) populations is a well-known societal quandary and a multifaceted phenomenon with loss of habitat, infection, and pesticide exposure representing the suspected causal factors. Here, we take a systems-level approach to addressing this issue by developing a honey bee-specific probiotic containing *Lactobacillus rhamnosus* GR-1, *Lactobacillus plantarum* Lp39, and *Lactobacillus kunkeei* BR-1. The rationale for this strategy is based on previous findings that demonstrate these specific lactobacilli to have pesticide detoxifying properties, immune boosting potential, and the capacity to improve host nutrient uptake, respectively. Results will be discussed from several field trials in which this triple-strain probiotic combination was supplemented to honey bees under a variety of experimental conditions and tested for its ability to A) vanquish *Paenibacillus* larvae (a nearly ubiquitous spore-forming bacterial pathogen and causal agent of American Foulbrood—the most serious brood disease of honey bees) from hives as a stand-alone supplement and as an adjunct treatment following guideline-recommended antibiotic administration, B) modulate microbiota diversity profiles of the eight to ten core phylotypes associated with nutrient partitioning in the adult honey bee gut, and C) alter gene expression of Cytochrome P450 enzymes involved in detoxification of environmentally-relevant pesticides. Altogether, this work suggests that probiotic lactobacilli can be used to simultaneously address multiple suspected causal factors implicated in honey bee decline and may represent a favorable alternative to current guideline-recommended treatment protocols. These findings provide a framework for the future study of how beneficial bacteria may mitigate population decline and improve productivity of this ecologically and economically important species.

### 11.6. Improving Health and Wealth with Affordable Bacterial Starter Cultures for Probiotic Yoghurt Production in Uganda

WesterikNieke^1^,^2^WacooAlex Paul^1^,^2^AnyimoEsther^3^MatovuWilliam^3^ReidGregor^4^,^5^KortRemco^1^,^2^,^6^SybesmaWilbert^1^1Yoba for Life foundation, 1079 WB Amsterdam, The Netherlands2Department of Molecular Cell Biology, VU University Amsterdam (VUA), 1081 HV, Amsterdam The Netherlands3Heifer Project International, Lule Rd, Kampala, Uganda4Canadian RandD Centre for Human Microbiome and Probiotics, Lawson Health Research Institute, London, ON N6C 2R5, Canada5Departments of Microbiology and Immunology, and Surgery, Western University, London, Ontario N6A 3K7, Canada6ARTIS-Micropia, 1018 CZ, Amsterdam, The Netherlands

In rural Africa, income generating activities of many households heavily depend on agricultural activities. In order to facilitate economic growth, there is a need for more income diversification through non-farm activities. Another need for African households relates to improving food safety and health, as can be concluded from the high incidence of bacterial infectious diseases and the presence of toxic contaminants in the food chain including aflatoxins. We present the results of a multi-year intervention whereby dairy farmers and small-scale entrepreneurs were taught to convert their milk into a probiotic yoghurt using an innovative bacterial starter culture and basic equipment. Demand was stimulated with an emphasis on pre-primary school-going children. This intervention creates additional sources of income and employment for people involved in the delivery of milk as well as production, distribution, and sales of yoghurt. Besides the economic benefits, the consumption of the probiotic yoghurt can contribute to reduction of the incidence and severity of diarrhea, respiratory tract infections, atopic diseases, alleviate the symptoms of stomach ulcers and decrease the uptake of aflatoxins in the body. With minimal external financial support, 116 communities or small entrepreneurs have been able to start, expand and maintain a business by production and sales of probiotic yoghurt. Applied business models and success rates of production units in terms of revenues and profitability varied per region and depended on location, culture, ownership structure, wealth status, and gender. As part of the intervention on the demand side, parents were encouraged to pay approximately 3 USD per school term of 3 months, for their child to take 125 ml of probiotic yoghurt daily, as produced by one of the local probiotic yoghurt producers. Preliminary results of this program are very promising, as in a period of 10 months already 6000 children enjoy probiotic yoghurt at school. A health impact study was recently conducted, following 584 children consuming yoghurt and 532 children consuming milk for 12 weeks (of which 3 weeks baseline). Study outcomes include the incidence of respiratory tract infections, skin diseases, as well as school attendance.

## 12. Discussion and Original Research Presentations

This session provided an opportunity for diverse discussion on a range of topics pertaining to human and/or environmental health. Original research was presented and discussed. All researchers were asked to explain how their work related to relevant global challenges.

### 12.1. Long-Chain Saturated Fatty Acids in Breast Milk are Associated with the Pathogenesis of Atopic Dermatitis via Induction of Inflammatory ILC3s

ShimojoNaoki^1^YamaideFumiya^1^NakanoTaiji^1^KongWeng-sheng^2^KannoMasamoto^2^1Department of Pediatrics, Graduate School of Medicine, Chiba University, Chiba, 263-8522, Japan2Department of Immunology, Hiroshima University, Hiroshima 739-0046, Japan

Breastfeeding influences development of the immune system in infants and may even affect various immunological responses later in life. Breast milk provides a rich source of early nutrition for infant growth and development. However, the presence of certain compounds in breast milk, related to an unhealthy lifestyle or the diet of lactating mothers, may negatively impact infants. Based on a birth cohort study, we found that the composition of mother’s milk containing high amounts of long-chain saturated fatty acids (LCSFAs) was related to a higher incidence of atopic dermatitis (AD) in children. Offspring breastfed by the mother taking high LCSFAs diet also developed AD-like eczema in a mouse model. We showed that LCSFAs are a type of damage-associated molecular patterns, which initiate a series of inflammatory events in the gut involving type 3 innate lymphoid cells (ILC3s). A remarkable increase in inflammatory ILC3s was observed in the gut, and it was suggested that the migration of these ILC3s to the skin contribute to the pathogenesis of eczema. Gene expression analysis of ILC3s isolated from the gut revealed upregulation of genes that increase ILC3s and chemokines, which may play a role in ILC migration to the skin. Rag1 knockout mice fed a high-LCSFA milk diet developed eczema, accompanied by increased gut ILC3s, indicating no pivotal role of adaptive immunity in this model.

### 12.2. Identification of MicroRNAs in Human Breast Milk Related to Atopic Dermatitis

Taiji Nakano (Department of Pediatrics, Graduate School of Medicine, Chiba University, Chiba, 263-8522, Japan)

**Background**: Breast milk is the optimal source of nutrition, protection, and developmental programming for infants. It consists of various bioactive components, including microRNAs, small non-coding RNAs regulating gene expression at the post-transcriptional level. The objective of this study was to describe the microRNA profile of breast milk related to atopic dermatitis (AD).

**Methods**: We collected breast milk on one month post-partum from the Chiba HIgh risk Birth cohort for Allergy Study (CHIBA study). Whole breast milk was fractionated by centrifugation to obtain three fractions including the cells, the lipid layer and skim milk. The profiles of microRNAs of lipid fraction and skim milk fraction were collected using 3D-Gene® Human miRNA Oligo chip. Differential expression of miRNA was assessed for AD group (n = 5) and non-atopic control group (n = 5). AD was diagnosed by a pediatrician at one year of age.

**Results:** hsa-miR-342-5p, hsa-miR-551b-5p and hsa-miR-3192-5p were downregulated in AD group compared to non-atopic group in lipid fraction. hsa-miR-551b-5p, hsa-miR-3192-5p and hsa-miR-4520-3p were downregulated in AD group compared to non-atopic group in skim milk fraction.

**Conclusion**: hsa-miR-342-5p, hsa-miR-551b-5p, hsa-miR-3192-5p and hsa-miR-4520-3p in breast milk might play role in development of AD at one year of age.

### 12.3. Extraction and Quantification of Breast Milk MicroRNAs with Potential Immune Regulating Properties

Emelie Ahlberg Lina Tingö, Maria Jenmalm (Institution of Clinical and Experimental Medicine, Linköping University, 581 83 Linköping Sweden)

MicroRNAs (miRNA) are short RNA sequences that inhibit protein translation by post-transcriptional modifications of the precursor mRNA. As such, miRNAs may be important regulators of immune-related pathways and epigenetic modifications in immune cells. miRNAs are found in various body fluids, including breast milk. Breast milk miRNA may be transferred from mother to infant and thereby potentially regulate the development of the baby’s immune system. In this study we have isolated miRNA from human breast milk and successfully quantified hsa-miR-148a-3p, evaluating seven different RNA extraction protocols and two different qPCR-based quantification methods. These include six commercially available spin-column based RNA extraction kits and a kit utilizing oligonucleotide-conjugated Dynal® magnetic beads. Breast milk miRNA isolates from 10 healthy lactating women four month post-delivery were subjected to single target qPCR with absolute quantification of hsa-miR-148a-3p (and cel-miR-39-3p as an exogenous spike-in). hsa-miR-148a-3p can be considered an important epigenetic regulator as one of its main targets is DNA methyltransferase 1, which for example is involved in promotor methylation of the *FOXP3* gene and hence regulatory T-cell (Treg) regulation. In addition, samples from four of the women, collected at the same time-point, were screened for 754 unique miRNA using TaqMan Advanced miRNA Array Cards A and B. Preliminary findings from this miRNA screening indicate that several potentially immune-related miRNAs are present in human breast milk samples four months after delivery, e.g., hsa-miR-24 and -27, known from previous research to be involved in T-helper (Th) type 2 cell differentiation. In addition, miRNAs that are important for regulation of Tregs, e.g., hsa-miR-126 and -142-3p, and proinflammatory cytokine production in macrophages and T-cells, such as hsa-miR-21-5p and -124-5p, were also expressed in the samples. This screening will be the basis for designing a customized miRNA array for relative quantification of immune-related human breast miRNAs in a randomized placebo-controlled allergy prevention trial, PROOM-3. The PROOM-3 trial is evaluating the effects of pre-and postnatal *Lactobacillus reuteri* and omega-3 polyunsaturated fatty acid supplementation from gestational week 20 through the first year of life in 480 women and their children.

### 12.4. Childhood Allergic Disorders: When and How do Urban Natural Spaces Matter?

SbihiHind^1^,^2^NesbittLorien^2^HystadPerry^3^BrookJeff R.^4^SearsMalcolm R.^5^SubbaraoPadmaja^6^MoraesTheo J.^6^MandhanePiush J.^7^BeckerAllan B.^8^AzadMeghan B.^8^van den BoschMatilda^2^TurveyStuart E.^1^,^2^1BC Children’s Hospital Institute, Vancouver, BC V5Z 4H4, Canada2University of British Columbia, Vancouver, BC V6T 1Z4, Canada3Oregon State University. Portland, Corvallis, OR 97331, USA4University of Toronto, Toronto, ON M5S 1X8, Canada5McMaster University. Hamilton, ON L8S 4L8, Canada6The Hospital for Sick Children, Toronto, ON M5G 1X8 Canada7University of Alberta, Edmonton, AB T6G 2R3, Canada8University of Manitoba, Winnipeg, MB R3T 2N2, Canada

**Background**: Urban vegetation plays an important role in reducing the adverse health effects incurred through the process of urbanization: it contributes to reducing harmful air pollutants and provides cooling during extreme heat events (1). Mounting evidence shows links to childhood allergic outcomes (2), but results are inconsistent. These contradictory results may be due to the varying methods chosen to assess urban greenness exposures. Here, we aim to evaluate different types of greenness exposures that reflect different pathways of influence in relation to risk of developing allergic rhinitis.

**Methods:** Using different data sources between 2008 and 2015, dates representing the period between conception and age five for most participants in the four-center Canadian Healthy Infant Longitudinal Development (CHILD) birth cohort, we derived and evaluated (i) three metrics measuring distinct aspect of greenness: a biomass measure using Normalized Difference Vegetation Index (NDVI) based on MODIS satellite images, a street-level measure using Google Street View (GSV), and a biodiversity measure using public tree census (Shannon diversity), and (ii) changes in greenness at the home addresses of all participants recruited in and followed up to age 5 years old, to pinpoint relevant time periods—in utero, first year of life, and lifetime cumulative exposure—for examining the effect of greenness exposure on allergic outcomes, including allergic rhinitis and sensitization, at 3 and 5 years of age.

**Results:** Each city showed distinct greenness patterns over time for assigned NDVI in a 250 m buffer around the residential addresses of CHILD participants. However, from 2010 onward increasing greenness (median NDVI) values were observed, except for Edmonton. For each year between 2008 and 2015, the spread of NDVI values varied distinctly for each CHILD study site, with Toronto and Winnipeg having the lowest interquartile ranges (Winnipeg: (0.05–0.09); Toronto: (0.04–0.06)). Similarly, GSV showed statistically significant differences across cities, with 20% and 18% of the images in Vancouver and Winnipeg being composed of trees. Preliminary results suggest differences between the greenness metrics on allergic rhinitis outcomes. For instance, children exposed to greenness in the first year of life were less likely to develop allergic rhinitis at age 5 years (adjusted OR = 0.63, 95% CI: (0.43–0.98) and aOR = 0.75 (0.53–1.04) using NDVI and GSV, respectively).

**Conclusion:** We find that greenness is protective against allergic rhinitis and discuss difference in effect estimates using different measures of greenness. These results warrant the following ongoing investigations: How do different time windows of exposure impact trajectories of disease? What underlying pathways do greenness metrics represent?

**Acknowledgement:** This work was supported by the Canadian Institutes of Health Research, the Allergy, Genes and Environment (AllerGen) Network of Centres of Excellence, and the Michael Smith Foundation for Health Research/AllerGen postdoctoral fellowships.

**Suggested Reading:** van den Bosch M, Ode Sang A. 2017. Urban natural environments as nature-based solutions for improved public health - A systematic review of reviews. Environ Res 158:373–384

Tischer C, Gascon M, Fernandez-Somoano A, Tardon A, Materola AL, Ibarluzea J, et al. Urban green and grey space in relation to respiratory health in children. European Respiratory Journal. 2017;49(6):1502112.

### 12.5. Asthma Endotypes Identified with Latent Class Analyses in Adolescents

HanXiaoxia^1^,^3^HavstadSuzanne L.^1^,^2^,^3^SitarikAlexandra R.^1^,^2^,^3^JohnsonChristine C.^1^,^2^,^3^Cassidy-BushrowAndrea E.^1^,^2^WegienkaGanesa R.^1^,^2^,^3^1Department of Public Health Sciences, Henry Ford Health System, Detroit, MI 48202, USA2Center for Urban Responses to Environmental Stressors, Wayne State University, Detroit, MI 48202, USA3inVIVO Planetary Health, Research Group of the Worldwide Universities Network (WUN), West New York, NJ 10704, USA

Asthma has been described as a syndrome or a “collection of several diseases with similar/same symptoms”. Endotypes, defined as similar phenotypes with different underlying pathobiology, have emerged recently as an approach to classification of disease entities. Defining these “asthma” endotypes may increase the likelihood of identifying risk factors that lead to more precisely defined adverse health states which, in turn, could lead to intervention strategies specific for these endotypes. The goal of this work was to identify “asthma” profiles using cross-sectional data from a racially diverse birth cohort of children ages 8–13 years in the Detroit, Michigan, USA area (the WHEALS cohort). These profiles were created with several latent indicators, including methacholine challenge (PC20), % predicted FEV1, % predicted FVC, FEF25-75, exhaled nitric oxide (eNO) and absolute levels of peripheral blood eosinophils and neutrophils—factors assumed to be involved in the disease process of asthma. By applying latent class analysis to these variables from 551 children in the WHEALS cohort, we identified four latent classes. Class types varied by latent indicators. Class 1 was characterized by low PC20, high eNO, low % predicted FEV1, low % predicted FVC, low FEF25-75 and low neutrophils. Class 2 was characterized by high PC20, low eNO, high % predicted FEV1, high % predicted FVC, high FEF25-75, and low eosinophils. Class 3 was characterized by low PC20, high eNO, normal % predicted FEV1, normal % normal % predicted FVC, and normal FEF25-75. Class 4 was characterized by high PC20, low eNO, low % predicted FEV1, and low FEF25-75.

The latent class membership was statistically significantly associated with several risk factors/health outcomes, including child’s atopic sensitization, BMI, % body fat, total IgE, doctor diagnosis of asthma, doctor diagnosis of atopic dermatitis/eczema at age 10, and prenatal vitamin D. Our study highlights the variability within the asthma syndrome and suggests a need for the refined assessment of disease classification using molecular-based allergy diagnostics and pulmonary function testing. Further, because the associations with risk factors differed between the profiles, this approach suggests that it may indeed improve identification of factors that could be used to tailor interventions for each particular endotype, as well as develop intervention strategies for all endotypes.

### 12.6. Associations between Environmental Factors and Skin Responsivities among Parent-Infant Pairs

ShinoharaMiwa^1^,^2^MatsumotoKenji^3^1Departments of Pediatrics, Juntendo University, Faculty of Medicine, Bunkyo City, Tokyo 113-0033, JAPAN2Departments of Pediatrics, Kochi University, Kochi, 780-8072, JAPAN3Department of Allergy and Clinical Immunology, National Research Institute for Child Health and Development, Setagaya City, Tokyo 157-0074, JAPAN

**Rationale**: Atopic dermatitis (AD) is a chronic recurrent inflammatory skin disorder caused by epidermal barrier dysfunctions and immune dysregulations induced by genetic and environmental factors. During infancy, the manifestations of AD vary according to age-specific stages; AD initially develops in the absence of allergic sensitization. We hypothesized that environmental factors initiate epidermal barrier dysfunction, thereby leading to AD development during infancy. To reveal the skin response-associated factors, we investigated the associations of skin responses to saline, histamine, purified protein derivative (PPD), atopic eczema/dermatitis syndrome (AEDS), and environmental factors during infancy.

**Methods:** In total, 256 infant–parent pairs were enrolled in this cross-sectional study. We administered self-written questionnaires about family size, infants’ daily yogurt intake, and physician-diagnosed AEDS. Skin prick tests (SPTs) for saline and histamine (1.0 mg/dL) were performed using bifurcated needles. SPT wheal sizes were recorded 15 min later. Skin responses to PPD were transcribed by the guardians using the maternal and child health handbooks.

**Results:** Skin responses to histamine, not to saline and PPD, significantly differed between infants with and without AEDS (4.4 ± 2.0 vs. 3.6 ± 1.9 mm; *p* = 0.04) and those with and without the yogurt intake (2.5 ± 1.4 vs. 3.8 ± 2.0 mm; *p* = 0.01). Conversely, skin responses to PPD, not to histamine and saline, were significantly positively associated with family size (*p =* 0.02) but not AEDS nor daily yogurt intake.

**Conclusions:** During infancy, AD may be modified by skin sensitivity to histamine and daily yogurt intakes but not by T cells reacting to microbiomes.

### 12.7. A Case for Meeting Micronutrient Needs of Children with ADHD

LeungBrenda^1^JohnstoneJeni^2^GraciousBarbara^3^,^4^PerezLeanna^3^TostGabriella^2^HatsuIrene^3^ArnoldL. Eugene^3^1University of Lethbridge, Lethbridge, Alberta T1K 3M4, Canada2Oregon Health and Science University, Portland, Oregon 97239, USA3The Ohio State University, Columbus, Ohio 43210, USA4Orange Park Medical Center, Orange Park, FL 32073, USA

The diagnosis of attention deficit hyperactivity disorder (ADHD) transcends age, gender, time, and ethnicity. Common risk factors include genetic, environmental, and social contributors. Growing evidence indicate vitamin and mineral deficiencies may be an important contributor. Several diet-related factors are proposed to influence ADHD symptoms, including nutrient insufficiencies, chronic inflammation, oxidative stress, mitochondrial dysfunction, and microbiome profile. Dietary quality has declined due to agricultural practices, soil erosion/depletion, and food processing over the past 100 years. While developing countries primarily experience deficiencies due to inadequate food intake, poor diet quality as a result of some micronutrients lacking plague high-income nations such as the United States and Canada. There is also growing evidence that nutrition plays a significant role in brain health. Studies have demonstrated improved cognition, mood, and behavior in both children and adults treated with micronutrient supplements. The purpose of this study was to investigate supplementation of broad spectrum micronutrients (vitamins and minerals) for children 6–12 years old with ADHD, irritable mood and oppositional behavior.

**Methods**: This study is an 8 week three-site randomized double-blind placebo-controlled trial (RCT) followed by eight weeks open label (OL). Measures include clinical assessment using the Child and Adolescent Symptom Inventory-5 (CASI-5), Patient-Reported Outcomes Measurement Information Systems (PROMIS), and biological samples: blood, hair, urine, stool, saliva. Preliminary analysis compared the OL data (weeks 8 to 16, following the RCT) on ADHD and irritability symptom counts, using a Wilcox Rank-Sum Test.

**Results**: A significant decrease in symptoms was found for inattention (*p* ≤ 0.001), hyperactivity (*p* ≤ 0.001), oppositionality (*p* ≤ 0.001), and dysregulated mood (*p* ≤ 0.001) (n = 27). Child anger, reported using the PROMIS by parent and child, improved in 79% and 74% of cases, respectively, during the OL phase.

**Conclusion**: Supplementation with micronutrients in children with ADHD and irritable mood showed a preliminary significant trend towards benefit for symptoms of ADHD, irritability, and anger in an open trial. These trends will later be tested with data from the RCT phase with full sample analyses.

### 12.8. Epigenetics of Neurodevelopmental Disorders Using Monozygotic Twins

MohandasNamitha^1^,^2^LokeYuk Jing^1^CromptonKylie^2^,^3^,^4^VadlamudiLata^5^,^6^CraigJeffrey M^1^,^2^,^7^1Environmental and Genetic Epidemiology Research, Murdoch Children’s Research Institute, Royal Children’s Hospital, Flemington Road, Parkville, Victoria, Australia2Department of Paediatrics, University of Melbourne, Flemington Road, Parkville, Victoria, Australia3Developmental Disability and Rehabilitation Research, Murdoch Children’s Research Institute, Flemington Road, Parkville, Victoria 3052, Australia4Neurodevelopment and Disability, The Royal Children’s Hospital, Flemington Road, Parkville, Victoria 3052, Australia5Centre for Clinical Research, Faculty of Medicine, The University of Queensland, Queensland, Australia6Royal Brisbane and Women’s Hospital, Queensland, Australia7Centre for Molecular and Medical Research, School of Medicine, Deakin University, Geelong, Victoria 3220, Australia

Neurodevelopmental disorders such as autism spectrum disorders (ASD), cerebral palsy (CP), and epilepsy are some of the most prevalent childhood neurological disorders caused by damage to the growth and development of the brain. Early life environments predispose children to later health outcomes. Epigenetics, which refers to modifications of DNA without change in DNA sequence, is one way by which environmental exposures may contribute to development of disease. DNA methylation, arguably the most highly studied epigenetic mark, has been correlated with early life environmental exposures and have implications in both disease mechanisms as well as clinical biomarkers of neurodevelopmental diseases. These modifications most likely originate in utero, in line with developmental origins of health and disease (DOHaD) hypothesis. The study of monozygotic (MZ) twins, in which genetics, age, sex, parental factors, and shared environment are controlled for, helps in distinguishing the extent of effect of genetics and environment. Discordance for neurodevelopmental disorders has been recorded in MZ twins indicating a potential role of non-shared environmental factors. The aim of this research was to utilize the discordant MZ twin model to understand epigenetic changes associated with neurodevelopmental disorders.

Genome-wide DNA methylation was measured within three MZ twin cohorts discordant for an ASD, CP, or epilepsy using Illumina’s Infinium HumanMethylation450 and EPIC arrays. Statistical and bioinformatics pipelines were used to analyze DNA methylation data.

DNA methylation analysis of CP-discordant twin pairs provides the first evidence that environmentally-mediated differential methylation in genes involved in known processes such as hypoxia and inflammation, and processes such as cell adhesion, may contribute to the development of CP. An epigenome-wide analysis of epilepsy discordant MZ twin pairs revealed distinct patterns of DNA methylation within subtypes of epilepsies of unknown cause. Differentially methylated genes within epilepsy subtypes included those with a role in metabolic pathways, voltage-gated channel signaling, and neurotransmitter processes.

This research paves the way for future larger studies, as understanding DNA methylation profiles associated with neurodevelopmental disorders, may facilitate biomarkers for earlier diagnosis. This could lead to possible intervention strategies for patients suffering from a broad spectrum of disorders. Analyzing epigenetic data from disease discordant twins provides an elegant study design and has the power to explore non-shared environmental factors that further refine models of disease mechanisms and biomarkers.

### 12.9. How Are Universities in the United States Using Innovations to Improve Sustainability in Their Operations?

Ruijia (Rose) Wang, Master student, Fletcher School, Tufts University, Medford 02155, USA

Roughly 4700 institutions of higher education in the United States collectively have 21 million students or 5.7 percent of the total population in America. Higher education institutions (HEI) play a crucial role in facilitating sustainable prosperity in universities. Campus operations serve one of the cornerstones among community outreach, research, curricula, and competences while engaging with university governance and sustainable assessment and reporting. It is vital for universities to not only focus on making campus sustainability commitments, but also integrating sustainable prosperity into concrete actions. Innovation for sustainability among campus operations is the central theme in this research. The goal is to provide a deeper understanding of how HEIs set goals for sustainability in their operations and the significance of using innovation to improve sustainability in universities. This research emphasis on the role of diffusion in the innovation process of campus operations among HEIs. Diffusion indicates communication mechanism and information exchange through uncertainty and newness.

Current sustainability assessment such as Sustainable Campus Index provided by the Association for the Advancement of Sustainability in Higher Education (AASHE) indicated some domains under operations, including air and climate, buildings, energy, food and dining, grounds, purchasing, transportation, waste, and water. Implementing effective campus operations for innovation for sustainability is essential to the integrity of knowledge generation. The University’s approach to sustainability has evolved into five interconnected pillars, including education, research, outreach, campus operations, and campus experiences. Campus operations as one of the five core elements among institutional framework require education and innovation. Research also indicates that campus operations have the highest sustainable development implementation outcomes among all five interconnected elements. Understanding the evolution of innovation process is also crucial to put diffusion into context, especially for the HEIs setting.

This paper aims to 1) highlight innovative sustainability initiatives among HEIs and demonstrate their impacts on campus operations and 2) discuss the process of utilizing innovation to improve sustainability in campus operations. Identifying needs and challenges in HEIs and developing a strategic framework can help in advancing sustainable prosperity in a meaningful way. This paper contributes to the literature by reflecting on how HEIs can promote sustainability, how innovation for sustainable prosperity is understood and can be improved in university operations in the United States.

### 12.10. Increasing Fracture Rate for Children in Western Australia between 2005 and 2015: A Matter of Concern?

RueterKristina^1^,^2^,^3^,^12^,^13^JenkinsMark^4^,^5^NimphiusSophia^4^,^5^HartNicolas H.^5^,^6^,^7^ChiversPaola^5^,^6^,^7^RantalainenTimo^5^,^6^,^7^,^8^BorlandMeredith L.^2^,^9^McIntyreFleur^5^,^9^StannageKatherine^1^,^10^SiafarikasAris^4^,^5^,^6^,^9^,^10^,^11^1Divison of Paediatrics, School of Medicine, The University of Western Australia, Nedlands, WA 6009, Australia2Emergency Department, Perth Children’s Hospital, Nedlands, WA 6009, Australia3Immunology Department, Perth Children’s Hospital, Nedlands, WA 6009, Australia4Centre for Exercise and Sports Science Research, Edith Cowan University, Perth, WA, Australia5Western Australian Bone Research Collaboration, Perth, WA 6009, Australia6Institute for Health Research, University of Notre Dame Australia, Fremantle WA 6160, Australia7Exercise Medicine Research Institute, Edith Cowan University, Joondalup WA 6027, Australia8Gerontology Research Center, University of Jyväskylä, 40014 Jyväskylän yliopisto, Finland9School of Health Sciences, University of Notre Dame Australia, Fremantle WA 6160, Australia10Department of Orthopaedic Surgery, Perth Children’s Hospital, Nedlands, WA 6009, Australia11Department of Endocrinology and Diabetes, Perth Children’s Hospital, Nedlands, WA 6009, Australia12Telethon Kids Institute for Child Health Research, Nedlands, WA 6009, Australia13inVIVO Planetary Health, Group of the Worldwide Universities Network (WUN), New York, USA

**Aim:** Western Australia is a state with unique geography and population distribution having only a single tertiary paediatric hospital (Princess Margaret/ Perth Children’s Hospital, PMH/ PCH in Perth) managing the majority of children and adolescents with fractures in the Emergency Department (ED). Fracture incidence in 0–16 year olds is known to be high and varies between countries with boys having a 1.5 fold higher fracture incidence than girls. There are no specific data for Australia. The aims of this study were to characterize presentations with upper and lower limb fractures to PMH-ED and compare trends in the incidence rate to population data.

**Methods**: This is a database audit of fracture presentations between 2005–2015 for fracture rates with a sub-analysis for gender, fracture site, and age, and a comparison to Perth Metropolitan and Western Australian population data.

**Results:** Analysis included a total of 31,340 presentations with fractures from 27,516 individual children (87.8%) comprising 24,480 children reporting one fracture (78.1%), and 3036 children reporting two or more fractures (9.7%). Fracture incidence, adjusted for the annual population size, increased from 0.63% in 2005 to 0.85% in 2015 (*p* < 0.001). The winter months had a higher incidence of fractures than the summer months. Males had a higher fracture incidence than females: 18,763 versus 12,577, ratio 1.5:1 (t = 4.589, *p* < 0.001), with upper limb fractures three times more common than lower limb fractures (*p* < 0.001). Fracture incidence increased with age until the early teenage years when a decline occurred.

**Conclusions:** Increased fracture incidence in children and adolescents in Western Australia between 2005 and 2015 identifies a concerning trend for bone health which cannot be explained by genetic factors. Further research is needed to identify potential lifestyle factors that impact bone health and fracture incidence in order to reverse fracture incidence trends seen in children and adolescents.

### 12.11. Does Vitamin D and Sunlight Exposure Influence Allergic Diseases in Early Childhood?

RueterKristina^1^,^2^,^3^,^4^JonesAnderson P^1^,^2^SiafarikasAris^1^,^2^,^3^LimEe-Mun^5^ClarkeMichael W^6^NoakesPaul S^2^PrescottSusan L^1^,^2^,^3^,^4^PalmerDebra J^1^,^2^1Divison of Paediatrics, School of Medicine, The University of Western Australia, Nedlands, WA 6009, Australia2Telethon Kids Institute for Child Health Research, Nedlands, WA 6009, Australia3Immunology Department, Perth Children’s Hospital, Nedlands, WA 6009, Australia4inVIVO Planetary Health, Group of the Worldwide Universities Network (WUN), New York, USA5Endocrinology Department, Sir Charles Gardiner Hospital, Nedlands, WA 6009, Australia6Metabolomics Australia, Centre for Microscopy, Characterisation and Analysis, University of Western Australia, Nedlands, WA 6009, Australia

**Background**: The incidence of childhood allergic disease has dramatically increased worldwide. Eczema is the earliest manifestation of allergic disease. Suboptimal vitamin D levels during critical periods of immune development have emerged as an explanation for higher rates of allergic diseases associated with industrialization and residing at higher latitudes. However, intervention trials investigating the effect of vitamin D supplementation during infancy as an allergy prevention strategy are lacking.

**Aims**: To determine the effects of early postnatal infant vitamin D supplementation and sunlight exposure on infant immune development and the development of eczema.

**Methods:** In this randomized controlled trial 195 high-risk infants were orally supplemented with either 400 IU vitamin D/day or placebo from birth to 6 months of age. Uniquely, a personal UV-dosimeter was worn to measure infant ultraviolet (UV) exposure during the first 3 months of life. Blood samples were collected at 3 and 6 months of age to determine relationships between oral vitamin D intake and UV-light exposure with blood 25(OH)D concentration, immune cell function responses to allergens and on the development of eczema by 6 months of age.

**Results:** At three (*p* < 0.001) and six (*p* = 0.023) months of age, vitamin D levels were higher for the vitamin D supplemented group than the placebo group, but there was no difference in eczema incidence between groups. Infants with eczema were found to have had less UV light exposure (median (IQR) 555 (322–1210) J/m2 compared to those without eczema (998 (676–1577) J/m2, *p* = 0.023). UV light exposure was also inversely correlated with IL-2, granulocyte-macrophage colony stimulating factor and eotaxin production to Toll-like receptor ligands.

**Conclusions:** This study is the first to demonstrate an association between higher direct UV light exposures in the first three months of life with lower incidence of eczema and pro-inflammatory immune markers by 6 months of age. Our six months findings indicate that UV light exposure appears more beneficial than vitamin D supplementation as an allergy prevention strategy in early life. The study is in progress and further results will be presented at the meeting.

## 13. Shaping New Norms: Pathways of Influence and Dimensions of Change (Session Summary and Abstracts)

This session focused on the strategies and pathways for change making, spanning from legal action, to social movements and specific community approaches.

### 13.1. KEYNOTE: Environmental Law—Litigation and Legal Channels in ‘Change Making’ for Social and Ecological Justice

Nicholas Schroeck is an attorney and Associate Professor of Law (University of Detroit Mercy School of Law Detroit, MI 48226, USA) where he directs the Environmental Law Clinic and has represented the Sierra Club, Natural Resources Defense Council, Environmental Law and Policy Center, Michigan Environmental Council, National Wildlife Federation and many others.

*No abstract available:* Nick Schroeck discussed the importance and effectiveness of legal challenges on the road to achieve environmental justice. In the United States, common law is a key path to changes when approaching vexing environmental problems. Under the ancient legal theory known as the Public Trust Doctrine, the state holds in trust for the benefit of its citizens the surface waters and submerged lands of the Great Lakes, and other resources subject to the trust. While each state’s public trust laws can vary, Schroeck discussed the doctrine as it has been applied in Michigan, with examples of legal successful challenges that have led directly to environmental change and remediation for the benefits of many communities. Schroeck also noted other areas where common law legal theories have been successful in litigation aimed at advancing environmental justice, particularly in the area of air quality.

### 13.2. A New Edge in Planetary Health? Sourcing Our Doing from a Different Quality of Being

PolandBlake^1^Mashford-PringleAngela^2^CohenRoxanne^3^BrisboisBen^4^CortinoisAndrea^1^PoggiAlexandra^5^1Dalla Lana School of Public Health, University of Toronto, Toronto, ON M5T 3M7, Canada2Waakebiness-Bryce Institute for Indigenous Health, DLSPH, University of Toronto, Toronto, ON M5T 3M7, Canada3Conscious Minds Co-operative, Toronto, ON M5T 3M7, Canada4Centre for Urban Health Solutions, St Michael’s Hospital, Toronto, Ontario M5B 1W8 Canada5Bloomalicious Life Coaching, St Colomban, Quebec, Canada

This paper explores the possibility that we are reaching the limits of what convention risk management, and even resilience thinking, are capable of, as the dominant prevailing responses to emerging planetary health challenges. Rather than trying to ‘solve’ or ‘fix’ problems with the same level of thinking that created them, we argue that new ways of seeing, thinking and being are required if change is to be truly transformational. Drawing on disparate sources spanning indigenous ways of knowing, Global South epistemologies, quantum physics and epigenetics, we paint a picture of an emerging landscape of possibilities as a new edge into which those engaged with Planetary Health issues could step to significantly ‘up their game’ as co-creators of a more beautiful world our hearts know is possible. A three-part process is envisaged: (a) deep, mindful, and conscious decolonization of our hearts and minds from the dominant world-views that keep us stuck in stress, reactivity, despair, and ineffectual action; (b) opening to time-honored animistic traditions that honor the sacredness and interconnectedness of all life as an enlivening alternative to the dominant mantra of separation, meaninglessness, survival-of-the-fittest competition and exploitation; and (c) drawing on the work of Bohm, Senge, Jayworski, Scharmer, and Dispenza, a process of stepping into a desired future with clear intention and elevated emotion, through the portal of deep presence. Along the way, we offer two modest examples of funded projects that we have undertaken along this path: Pedagogy for the Anthropocene (or P4A), and the Many Lenses Project. We conclude with a roadmap of where we think this could go next, and an invitation to co-create.

**Suggested Reading**: Redvers, N. (2018). The value of global Indigenous knowledge in planetary health. *Challenges*, *9*(2).

Harding, S. (2006). Animate Earth: Science, Intuition and Gaia. Chelsea Green.

Abram, D. (1996). *The Spell of the Sensuous*. Vintage Books.

Senge, P., Scharmer, C. O., Jaworski, J., & Flowers, B. S. (2004). *Presence: An Exploration of Profound Change in People, Organizations, and Society*. Currency/Doubleday/Random House.

Scharmer, C. O. (2009). Theory U: Leading From the Future as It Emerges. Berrett-Koehler.

Jaworski, J. (2012). Source: The Inner Path of Knowledge Creation. Berrett-Koehler.

Dispenza, J. (2017). Becoming Supernatural: How Common People are Doing the Uncommon. Hay House.

Barad, K. (2007). Meeting the Universe Halfway: Quantum Physics and the Entanglement of Matter and Meaning. Duke University Press.

Wendt, A. (2015). Quantum Mind and Social Science: Unifying Physical and Social Ontoloty. Cambridge University Press.

### 13.3. Engaging the Public with Concepts of Ecology and Mutualism: Micropia, the World’s Only Microbe Museum

Remco Kort (ARTIS-Micropia Chair, Department of Molecular Cell Biology, VU University Amsterdam, De Boelelaan 1108, 1081 HZ Amsterdam, The Netherlands)

The Anthropocene as the new geological era presents us with urgent questions concerning our relationship with nature. The royal zoological society Natura Artis Magistra (ARTIS), founded in 1838, seeks to answer such questions by renewing its outreach strategy to its public of 1.4 million visitors a year. This strategy includes educational tools for schools, programming in its zoological and botanical park, its planetarium, its lecture series known as the ARTIS academy and Micropia, the world’s only microbe museum. One way to demonstrate the interconnectedness of humans with nature is through the world of microbes. Insight in the presence and activity of these invisible, but ubiquitous life forms becomes more and more important in times of overpopulation and scarcity, as microbes play a crucial role in all nutrient cycles. Their importance is not only evident for the ecosystems of our planet, but also for our health. The metabolic activity of microbes in our body forms an often neglected, but intrinsic factor of human physiology. Moreover, there is an accumulating amount of evidence that the lack of exposure to a diversity of microbes, associated with our modern lifestyle in urban areas, contributes to the current epidemic of chronic inflammatory diseases. Micropia discloses microbial life and brings awareness for its aforementioned importance to our health and our planet through its science platform and its thematic interactive exhibits with over 200 living microbial species.

### 13.4. Thoughts into Interdisciplinary Action: A 12-Step Programme for Earth

ColeJennifer^1^FosterAlex^2^FarlowAndrew^3^PetrikovaIvica^4^CheemaKiran^1^1Department of Geography, Royal Holloway University of London, Egham TW20 0EX, UK2School of Anthropology and Museum Ethnography, Oxford University, Oxford OX2 6PE, UK3Oxford Martin School, Oxford University, Oxford OX1 3BD, UK4Department of Politics and International Relations, Royal Holloway University of London, Egham TW20 0EX, UK

The growing academic interest in Planetary Health as an approach to environmental and human health challenges has resulted in exploration of solutions that are often framed from within the PESTLE (political, economic, social, technological, legal, and environmental) approach popular in occupational theory or its variant STEEPLE: socio-demographic, technological, economic, environmental, political, legal, and ethical). The potential of a stronger ethical code as a solution to challenges has received less attention than other PESTLE/STEEPLE factors, however. To address this, authors of this paper drafted and published a draft code of ethics for Planetary Health and have developed a methodology that will enable wide consultation on the first draft.

The second draft of the ethics will be an iterative process which will require input from as many individuals, organizations, sectors, and geographic regions as possible. To enable this, a discussion forum has been created on the peer-to-peer news sharing and commentary platform reddit (www.reddit.com/r/planetaryhealthethics), open to academic and non-academic contributors. Our previous research has identified reddit as a suitable platform for such a consultation process.

As reddit requires users to have access to internet-enabled ICT devices, access to electricity, literacy, and ability to operate in written English, additional provision has been made for communities that may be excluded by such requirements, particularly those from the Global South, to be able to contribute through the development of focus group materials that can be delivered verbally in local languages.

The first focus group consultation to test the methodology was held in Likubula, in Liwonde National Park, Malawi in March 2019. The consultation suggests that the ethical code is broadly acceptable but may need to be mindful of wording that frames interaction with ecosystems as “looking backwards” to a world in which humans are more integrated with nature, especially in regions of the world that are lagging behind international targets for progress, if this can be taken to imply that progress might be stalled or impeded. Calling for ‘debate’ might also be less popular with communities that feel their voice is less likely to be heard over others. Ethical approaches stressing intergenerational equity and community consultation were particularly valued.

The focus group demonstrated that the materials provided are workable in low-income settings and suggests that a one-size-fits-all code, if it is possible to achieve at all, will need modification of the current draft through further iterations.

### 13.5. It Is Still “Think Globally, Act Locally”

HancockTrevor

The concept of thinking globally but acting locally has been an essential part of the environmental movement’s approach for decades. This phrase has been variously attributed to Buckminster Fuller, David Brower (founder of Friends of the Earth), and René Dubos—all calling for social and environmental responsibility, while underscoring the importance and power of local agency to make this a reality. Meanwhile, Americans, Canadians, and Australians use the equivalent of five planets’ worth of biocapacity annually. If the entire planet were to live the way we live, we would need four more Earths. Overall, high-income countries (HICs) on average used 3.6 times the available biocapacity, meaning we would need two or three more planets. However, despite the fantasies of Elon Musk and his followers, there are no other planets available to us—there is no Planet B. Therefore, we have to learn to live in a healthy way with an ecological footprint of just one planet. For HICs as a whole, this will require about a 70 percent reduction, while for the USA, Canada, and Australia it requires an 80 percent reduction in the footprint. This is a massive and unprecedented challenge.

However, while desirable, top-down change is unlikely, given the power, wealth, and other benefits that accrue to those at the top of the current socio-economic and political system, so change will mainly have to come from below, from the cities and towns where more than half the world’s population now lives, although as Fran Baum’s ‘nutcracker’ analogy suggests, a combination of bottom-up and top-down would be best. Hence the emerging concept of One Planet Living and One Planet Regions, which I sometimes frame as ‘Healthy Cities 2.0’. A ‘One Planet Region’ is a place that has a high quality of life and good health for all while living within the limits of this one small planet that is our home. I am working on this challenge with others in my own community—the Greater Victoria Region (GVR), capital of the Canadian province of British Columbia—to raise awareness and stimulate change across all sectors of our community.

**Suggested reading**: T. Hancock. Beyond Science and Technology: Creating Planetary Health Needs Not Just ‘Head Stuff’, but Social Engagement and ‘Heart, Gut and Spirit’ Stuff. Challenges 2019, 10(1), 31 (for full paper on this topic):

T. Hancock, A. Capon, M. Dooris, R, Patrick. One Planet Regions: Planetary Health at the Local Level. Lancet Planetary Health 2017, 1, e92–e93.

T. Hancock, Healthy cities 2.0: Transitioning towards ‘one planet’ cities (key challenges facing 21st century cities, part 3). Cities Health 2018, 2, 22–25.

## 14. Conclusions: Taking It Forward

Our goal is to create a collaborative community that not only creates new research opportunities, but that also provides advocacy and influence as we contribute to important global conversations. While our agenda is broad, we see the diverse topics presented here as different facets of a central challenge—living in the Anthropocene [3].

Our agenda focused not only on the challenges, but on the opportunities and potential solutions: the tools, the vision, and the pathways to get there. We recognize that this requires a deeper understanding of the biology of complexity and the ways in which all systems are interdependent and interconnected. This necessitates integrated perspectives for multilateral solutions that seek to minimize adversity and recognize the value and promote protective factors. It also requires a greater awareness of how to motivate change for individuals, communities, and societies at large. While building awareness of the threats and dangers is important, so is the hope that change is possible [4]. People need tangible pathways to engage in change and guidance on meaningful ways of making change. Most important of all, we need to believe our actions can make a difference [5,6].

This speaks to the vital role of local “grass roots” initiatives in each and every community—where change can be made, felt, and made most meaningful. Global change begins locally, and a personal commitment to planetary health is the most powerful change that we can make.

## Figures and Tables

**Figure 3:**
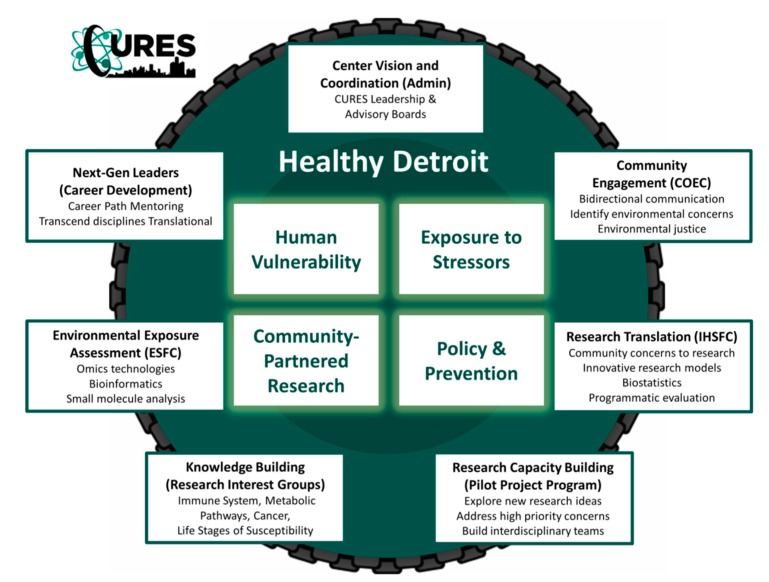
The Center for Urban Responses to Environmental Stressors (CURES)’ approaches to complex urban exposures and public health.
